# New Isoxazolidine-Conjugates of Quinazolinones—Synthesis, Antiviral and Cytostatic Activity

**DOI:** 10.3390/molecules21070959

**Published:** 2016-07-22

**Authors:** Dorota G. Piotrowska, Graciela Andrei, Dominique Schols, Robert Snoeck, Magdalena Grabkowska-Drużyc

**Affiliations:** 1 Bioorganic Chemistry Laboratory, Faculty of Pharmacy, Medical University of Lodz, Muszyńskiego 1, 90-151 Lodz, Poland; magdalena.grabkowska-druzyc@umed.lodz.pl; 2 Rega Institute for Medical Research, KU Leuven, Minderbroedersstraat 10, B-3000 Leuven, Belgium; Graciela.Andrei@rega.kleuven.be (G.A.); Dominique.Schols@rega.kleuven.be (D.S.); Robert.Snoeck@rega.kleuven.be (R.S.)

**Keywords:** phosphonate, isoxazolidine, quinazolinones, antiviral, cytostatic

## Abstract

A novel series of (3-diethoxyphosphoryl)isoxazolidines substituted at C5 with various quinazolinones have been synthesized by the 1,3-dipolar cycloaddition of *N*-methyl-C-(diethoxyphosphoryl)nitrone with *N*3-substitued 2-vinyl-3*H*-quinazolin-4-ones. All isoxazolidines were assessed for antiviral activity against a broad range of DNA and RNA viruses. Isoxazolidines *trans*-**11f**/*cis*-**11f** (90:10), *trans*-**11h** and *trans*-**11i**/*cis*-**11i** (97:3) showed weak activity (EC_50_ = 6.84, 15.29 and 9.44 μM) toward VZV (TK^+^ strain) which was only one order of magnitude lower than that of acyclovir used as a reference drug. Phosphonates *trans*-**11b**/*cis*-**11b** (90:10), *trans*-**11c**, *trans*-**11e**/*cis*-**11e** (90:10) and *trans*-**11g** appeared slightly active toward cytomegalovirus (EC_50_ = 27–45 μM). Compounds containing benzyl substituents at N3 in the quinazolinone skeleton exhibited slight antiproliferative activity towards the tested immortalized cells with IC_50_ in the 21–102 μM range.

## 1. Introduction

Nitrogen-containing heterocycles form the core of natural products (e.g., alkaloids) and they are also present in many pharmacophores as well as in numerous marketed drugs. Among them, quinazolines and quinazolinones have drawn special attention due to the broad spectrum of biological activities of their derivatives, including sedative [1,2,3], anticancer [4,5,6,7], antiviral [8,9,10,11,12], antibacterial [13,14,15], antifungal [15,16], anti-inflamatory [15,17,18,19] and antifibrotic [20,21] activities. Several reviews focused on the synthetic strategies and biological activities of these compounds have been published [22,23,24,25,26,27,28,29]. The significant impact of various functional groups installed into quinazoline/quinazolinone frameworks on pharmacological properties have been proven.

**Figure 1:**
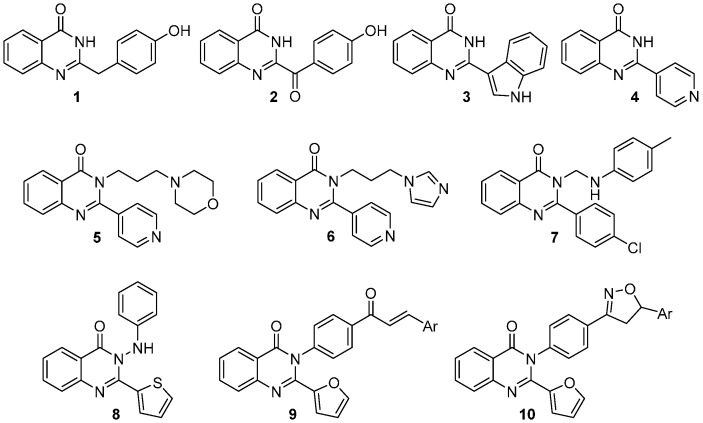
Examples of quinazolinones with antiviral and anticancer activity.

In the last decades several compounds containing the quinazolin-4-one framework, which exhibited promising anticancer as well as antiviral properties, have been obtained (Figure 1). Furthermore, some biologically active substituted quinazolin-4(3*H*)-ones were isolated from various fungi and bacteria species. For example, 2-(4-hydroxybenzyl)quinazolin-4(3*H*)-one (**1**) was found in an entomopathogenic fungus *Isaria farinosa* and its strong inhibitory properties on the replication of tobacco mosaic virus (TMV) [30] were recognised, whereas its 2-(4-hydroxybenzoyl) analogue **2** present in fungus from *Penicillium* genus appeared only slightly active toward TMV [30]. Moreover, compound **1** exhibited significant cytotoxicity toward various cancer cell lines [31,32]. Quinazolinone **3** isolated from *Streptomyces* sp. appeared cytotoxic against Vero cells [33]. Very recently synthetic pyridine-containing analogue **4** and its 3-substituted derivatives **5** and **6** have been obtained and their slight activity against influenza A virus was revealed [34]. On the other hand, various 2,3-disubstitued quinazolin-4(3*H*)-ones, including compounds **7**–**10**, have been found to possess antitumor activity [35].

**Scheme 1:**
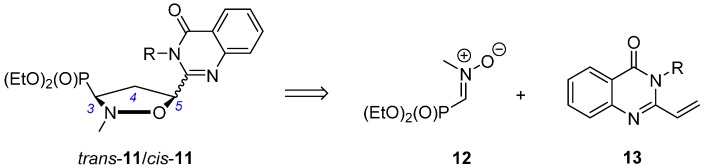
Retrosynthesis of (isoxazolidinyl) phosphonates *trans*-**11**/*cis*-**11**.

In continuation of our studies on antiviral and cytostatic activity of isoxazolidine analogues of C-nucleoside analogues, we designed a new series of compounds of the general formula **11** containing a substituted quinazolinone moiety as a false nucleobase at C5 in the isoxazolidine ring and the diethoxyphosphoryl function attached at C3. Our synthetic strategy to compounds *trans*-**11**/*cis*-**11** relies on the 1,3-dipolar cycloaddition of *N*-methyl-*C*-(diethoxyphosphoryl)nitrone **12** [36] with 2-vinyl-3*H*-quinazolin-4-ones **13** substituted at N3 (Scheme 1).

## 2. Results and Discussion

### 2.1. Chemistry

**Scheme 2:**
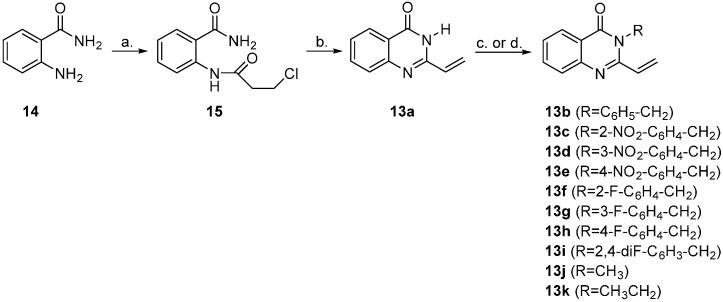
Synthesis of Compounds **13a**–**k**. *Reaction and conditions*: a. 3-chloropropionyl chloride, 1,4-dioxane, 0 °C; b. 5% NaOH-EtOH (2:1); c. RBr, KOH, CH_3_CN, 105 °C; d. MeI or EtI, KOH, CH_3_CN, 60 °C.

2-Vinyl-3*H*-quinazolin-4-ones **13** modified at N3 with substituted benzyl groups were synthesized from commercially available 2-aminobenzamide (**14**) by acylation with 3-chloro-propionyl chloride followed by cyclization and dehydrohalogenation to prepare 2-vinyl-3*H*-quinazolin-4-one (**13a**) as a key intermediate [37] and a subsequent reaction with substituted benzyl bromides **13b**–**i** [38] (Scheme 2). Moreover, compounds **13j** (R = Me) and **13k** (R = Et) were also obtained with intention to determine the influence of the benzyl substituent on biological activity of the designed isoxazolidines *trans*-**11**/*cis*-**11**. In the ^1^H-NMR spectra of compounds **13a**–**k** characteristic signals for vinyl protons were observed in the 6.94–5.59 ppm (three doublets of doublets).

**Scheme 3:**
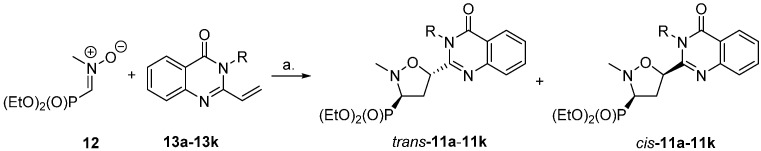
Synthesis of Isoxazolidines *cis*-**11a**–**k** and *trans*-**11a**–**k**. *Reaction and conditions*: a. toluene, 70 °C, 24 h.

The 1,3-dipolar cycloaddition of a nitrone **12** with 2-vinylquinazolinones **13a**–**k** led to the formation of diastereoisomeric mixtures of 5-substituted (3-diethoxyphosphoryl)isoxazolidines *trans*-**11** and *cis*-**11** with good (80%–88%) diastereoselectivities (Scheme 3, Table 1). Ratios of *cis/trans* diastereoisomers were calculated from ^31^P-NMR spectra of crude reaction mixtures and confirmed by the analysis of ^1^H-NMR spectral data. Crude mixtures of isoxazolidine cycloadducts were then subjected to purification on silica gel columns. However, attempts to isolate pure diastereoisomers were fruitful for *trans*-**11a** (R = H), *trans*-**11c** (R = 2-NO_2_-C_6_H_4_-CH_2_), *trans*-**11g** (R = 3-F-C_6_H_4_-CH_2_), *trans*-**11h** (R = 4-F-C_6_H_4_-CH_2_) and *trans*-**11j** (R = Me) only.

**Figure 2:**
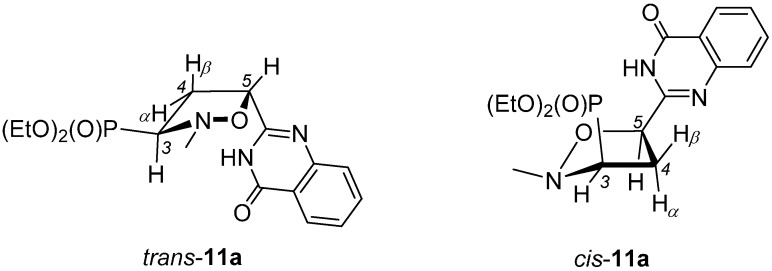
The preferred conformations of trans-isoxazolidine *trans*-**11a**.

The relative configurations of isoxazolidines *trans*-**11a** and *cis*-**11a** were established based on our previous studies on stereochemistry of cycloaddition of *N*-methyl-C-(diethoxyphosphoryl)nitrone (**12**) with various vinyl aryls [39,40] since similar ^1^H-NMR spectral patters for the respective series of *trans*- and *cis*-isoxazolidines were observed. Since for compound *trans*-**11a** all necessary coupling constants were successfully extracted from the ^1^H- and ^13^C-NMR spectra, detailed conformational analysis was performed based on these data {*J*_(H3-H4__α)_ = 9.3 Hz [41], *J*_(H3-H4__β)_ = 8.3 Hz, *J*_(H4__α-P)_ = 9.9 Hz [42,43], *J*_(H4__β-P)_ = 16.9 Hz, *J*_(H4__α-H5)_ = 6.2 Hz, *J*_(H4__β-H5)_ = 8.3 Hz, *J*_(CCCP)_ = 8.5 Hz [44,45]} and revealed that isoxazolidine ring in *trans*-**11a** adopts a _3_*E* conformation in which the diethoxyphosphoryl group resides in the equatorial position of the isoxazolidine ring while a quinazolinone substituent is located pseudoequatorially (Figure 2). On the other hand, cis configuration of the minor isomer was established from the corresponding couplings [*J*_(H3-H4__α)_ = 9.0 Hz, *J*_(H3-H4__β)_ = 6.5 Hz, *J*_(H4__α-P)_ = 11.2 Hz, *J*_(H4__β-P)_ = 20.0 Hz, *J*_(H4__α-H5)_ = 9.1 Hz, *J*_(H4__β-H5)_ = 3.9 Hz, *J*_(CCCP)_ = 7.3 Hz] indicating the _2_*E* conformation of the isoxazolidine ring (Figure 2). The additional arguments to support our assignments follow from shielding of the CH_3_CH_2_OP protons observed for the cis isomer (Δδ *ca.* 0.1 ppm) when compared with the *trans*-**11a**. Furthermore, it was found that on a ^1^H-NMR spectrum taken on the 83:17 mixture of *cis*- and *trans*-**11a**, the H-N proton in the quinazolinone ring of *cis*-**11a** was significantly deshielded (Δδ = 0.7 ppm) when compared with the trans isomer, highly likely, as a result of the hydrogen bond formation with the phosphoryl oxygen amide, a phenomenon spatially achievable in the cis isomer only.

Since introduction of various substituents at N3 of quinazolinone moiety has no influence on the stereochemical outcome of the cycloaddition therefore configuration of the all major isoxazolidines **11** were assigned as *trans*, thereby minor ones as *cis*.

### 2.2. Antiviral and Cytostatic Evaluation

#### 2.2.1. Antiviral Activity

All obtained phosphonates *trans*-**11a**, *trans*-**11c**, *trans*-**11g**, *trans*-**11h** and *trans*-**11j** as well as mixtures of diastereoisomeric phosphonates *trans*-**11b**/*cis*-**11b** (90:10), *trans*-**11d**/*cis*-**11d** (90:10), *trans*-**11e**/*cis*-**11e** (90:10), *trans*-**11f**/*cis*-**11f** (90:10), *trans*-**11i**/*cis*-**11i** (97:3) and *trans*-**11k**/*cis*-**11k** (97:3) were evaluated for inhibitory activity against a wide variety of DNA and RNA viruses, using the following cell-based assays: (a) human embryonic lung (HEL) cells: herpes simplex virus-1 (KOS), herpes simplex virus-2 (G), thymidine kinase deficient (acyclovir resistant) herpes simplex virus-1 (TK^−^ KOS ACV^r^), vaccinia virus, adeno virus-2, vesicular stomatitis virus, cytomegalovirus (AD-169 strain -1 and Davis strain), varicella-zoster virus (TK^+^ VZV Oka strain and TK^−^ VZV 07-1 strain); (b) HeLa cell cultures: vesicular stomatitis virus, Coxsackie virus B4 and respiratory syncytial virus; (c) Vero cell cultures: para-influenza-3 virus, reovirus-1, Sindbis virus, Coxsackie virus B4, Punta Toro virus, yellow fever virus; (e) CRFK cell cultures: feline corona virus (FIPV) and feline herpes virus (FHV) and (d) MDCK cell cultures: influenza A virus (H1N1 and H3N2 subtypes) and influenza B virus. Ganciclovir, cidofovir, acyclovir, brivudin, zalcitabine, zanamivir, alovudine, amantadine, rimantadine, ribavirin, dextran sulfate (molecular weight 10,000, DS-10000), mycophenolic acid, Hippeastrum hybrid agglutinin (HHA) and *Urtica dioica* agglutinin (UDA) were used as the reference compounds. The antiviral activity was expressed as the EC_50_: the compound concentration required to reduce virus plaque formation (VZV) by 50% or to reduce virus-induced cytopathogenicity by 50% (other viruses).

Several isoxazolidines *trans*-**11**/*cis*-**11** were able to weakly inhibit the replication of TK^+^ and TK^−^ VZV strains with EC_50_ values in the range of 6.84–100 μM (Table 2). Among them, phosphonates *trans*-**11f**/*cis*-**11f** (90:10) (R = 2-F-C_6_H_4_-CH_2_) (EC_50_ = 6.84 μM), *trans*-**11h** (R = 4-F-C_6_H_4_-CH_2_) (EC_50_ = 15.29 μM), *trans*-**11i**/*cis*-**11i** (97:3) (R = 2,4-diF-C_6_H_3_-CH_2_) (EC_50_ = 9.44 μM) were the most active toward TK^+^ VZV Oka strain, while exhibiting no activity toward TK^−^ VZV strain. The activity of these isoxazolidines *trans*-**11**/*cis*-**11** against TK^+^ VZV Oka strain was 8- to 22-folds lower than that of the reference drug acyclovir.

On the other hand, the EC_50_ values for the TK^−^ VZV 07-1 strain (which is an acyclovir resistant strain) of the phosphonates *trans*-**11e**/*cis*-**11e** (90:10) (R = 4-NO_2_-C_6_H_4_-CH_2_) (EC_50_ = 42.87 μM) and *trans*-**11k**/*cis*-**11k** (97:3) (R = Et) (EC_50_ = 41.57 μM) were comparable to that of acyclovir (EC_50_ = 39.69 μM). These derivatives showed similar EC_50_’s for TK^+^ and TK^−^ VZV strains and therefore their potency against TK^+^ VZV was approximately 50-fold lower compared to acyclovir.

Furthermore, compounds *trans*-**11b**/*cis*-**11b** (90:10) (R = C_6_H_5_-CH_2_), *trans*-**11c** (R = 2-NO_2_-C_6_H_4_-CH_2_), *trans*-**11e**/*cis*-**11e** (90:10) (R = 4-NO_2_-C_6_H_4_-CH_2_) and *trans*-**11g** (R = 3-F-C_6_H_4_-CH_2_) showed some activity against human cytomegalovirus (EC_50_ = 27–45 μM), although they were less active than ganciclovir and cidofovir used as the reference compounds (Table 3). None of the phosphonate derivatives here described showed activity against the other tested DNA and RNA viruses.

#### 2.2.2. Cytostatic Activity

The 50% cytostatic inhibitory concentration (IC_50_) causing a 50% decrease in cell proliferation was determined against murine leukemia L1210, human lymphocyte CEM, human cervix carcinoma HeLa and immortalized human dermal microvacsular endothelial cells (HMEC-1). Isoxazolidines *trans*-**11a** (R = H) and *trans*-**11j** (R = Me) did not inhibit cell proliferation at the highest tested concentration (i.e., 250 μM), whereas *trans*-**11k**/*cis*-**11k** (97:3) (R = Et) appeared slightly cytostatic towards the tested cell lines (IC_50_ = 85–101 μM). On the other hand (Table 4, entries **b** to **i**), compounds having benzyl substituents at N3 in the quinazolinone moiety showed lower IC_50_ values (IC_50_ = 21–102 μM) thereby indicating that installation of functionalized benzyl groups was profitable for inhibitory properties.

## 3. Experimental Section

### 3.1. General

^1^H-NMR spectra were taken in CDCl_3_ on the following spectrometers: Gemini 2000BB (200 MHz Varian, Palo Alto, CA, USA), and Avance III (600 MHz, Bruker Instruments, Karlsruhe, Germany) with TMS as internal standard. ^13^C-NMR spectra were recorded for CDCl_3_ solution on the Bruker Avance III at 151.0 MHz. ^31^P-NMR spectra were performed in CDCl_3_ solution on the Varian Gemini 2000 BB at 81.0 MHz or on Bruker Avance III at 243.0 MHz. IR spectra were measured on an Infinity MI-60 FT-IR spectrometer (ATI Instruments North America-Mattson, Madison, WI, USA). Melting points were determined on a Boetius apparatus (VEB Kombinat NAGEMA, Dresden, Germany) and are uncorrected. Elemental analyses were performed by the Microanalytical Laboratory of this Faculty on Perkin-Elmer PE 2400 CHNS analyzer (Perkin-Elmer Corp., Norwalk, CT, USA). The following adsorbents were used: column chromatography, Merck silica gel 60 (70–230 mesh); analytical TLC, Merck (Merck KGaA, Darmstadt, Germany) TLC plastic sheets silica gel 60 F_254_. *N*-methyl-*C*-(diethoxyphosphoryl)nitrone (**12**) [36], 2-vinyl-3*H*-quinazolin-4-one (**13a**) [37] and 3-methyl-2-vinyl-3*H*-quinazolin-4-one (**13j**) [38] were obtained according to the literature procedures.

^1^H-, ^13^C- and ^31^P-NMR spectra of all new synthesised compounds are provided in Supplementary Materials (Figures S1–S54).

### 3.2. General Procedure for the Synthesis of N*3*-Benzylated 2-Vinyl-3H-quinazolin-4-ones ***13b**–**i***

To the solution of 2-vinyl-3*H*-quinazolin-4-one (**13a**, 1.00 mmol) in acetonitrile (15 mL) potassium carbonate (3.00 mmol) was added. After 15 min the respective benzyl bromide (1.10 mmol) was added and the reaction mixture was stirred under reflux for 4 h. A solvent was removed and the residue was extracted with water (3 × 10 mL). An organic layer was dried (MgSO_4_), concentrated and the crude product was purified on a silica gel column with a methylene chloride: hexane mixture (7:3, *v*/*v*) followed by crystallisation (chloroform-petroleum ether) to give pure quinazolinones **13b**–**e** and **13g**–**i**.

*3-Benzyl-2-vinylquinazolin-4(3H)-one* (**13b**). White amorphous solid, m.p. = 85 °C–87 °C (reference [46]—colorless viscous oil). IR (KBr, cm^−1^) ν_max_: 3027, 2950, 2925, 1677, 1615, 1574, 1425, 1312, 1271, 952, 845, 778, 755, 585. ^1^H-NMR (200 MHz, CDCl_3_): δ = 8.20–8.19 (m, 1H), 7.92–7.88 (m, 1H), 7.83–7.78 (m, 1H), 7.58–7.49 (m, 3H), 7.46–7.36 (m, 3H), 6.94 (dd, ^3^*J* = 17.2 Hz, ^3^*J* = 10.1 Hz, 1H, C*H*=CH_2_), 6.73 (dd, ^3^*J* = 17.2 Hz, ^2^*J* = 2.3 Hz, 1H, CH=C*H*_2_), 5.80 (dd, ^3^*J* = 10.1 Hz, ^2^*J* = 2.3 Hz, 1H, CH=C*H*_2_), 5.69 (s, 2H, N-CH_2_). ^13^C-NMR (151 MHz, CDCl_3_): δ = 166.12 (s, C(O)), 159.97, 151.63, 137.17, 136.44, 133.57, 128.60, 128.28, 128.25, 127.67, 126.60, 123.81, 123.61, 115.59, 68.32 (s, N-CH_2_). Anal. Calcd. for C_17_H_14_N_2_O: C, 77.84; H, 5.38; N, 10.68. Found: C, 77.69; H, 5.21; N, 10.88.

*3-(2-Nitrobenzyl)-2-vinylquinazolin-4(3H)-one* (**13c**). Yellowish amorphous solid, m.p. = 88 °C–91 °C. IR (KBr, cm^−1^) ν_max_: 2952, 2851, 1615, 1574, 1525, 1493, 1396, 1312, 988, 1104, 988, 938, 780, 723, 664. ^1^H- NMR (600 MHz, CDCl_3_): δ = 8.24–8.22 (m, 1H), 8.18–8.16 (m, 1H), 7.96–7.94 (m, 1H), 7.87–7.82 (m, 2H), 7.69–7.67 (m, 1H), 7.59–7.53 (m, 2H), 6.91 (dd, ^3^*J* = 17.2 Hz, ^3^*J* = 10.4 Hz, 1H, C*H*=CH_2_), 6.64 (dd, ^3^*J* = 17.2 Hz, ^2^*J* = 1.2 Hz, 1H, CH=C*H*_2_), 6.14 (s, 2H, N-C*H*_2_), 5.78 (dd, ^3^*J* = 10.4 Hz, ^2^*J* = 1.2 Hz, 1H, CH=C*H*_2_). ^13^C-NMR (151 MHz, CDCl_3_): δ = 165.51 (s, C(O)), 159.83, 151.80, 147.86, 136.77, 133.78, 133.65, 132.77, 129.02, 128.67, 128.86, 127.86, 126.85, 124.97, 124.12, 123.56, 123.26, 115.26, 64.79 (s, N-CH_2_). Anal. Calcd. for C_17_H_13_N_3_O_3_: C, 66.44; H, 4.26; N, 13.67. Found: C, 66.23; H, 4.11; N, 13.38.

*3-(3-Nitrobenzyl)-2-vinylquinazolin-4(3H)-one* (**13d**). Yellowish amorphous solid, m.p. = 90 °C–93 °C. IR (KBr, cm^−1^) ν_max_: 3093, 3020, 2958, 2941, 1615, 1574, 1562, 1523, 1345, 1044, 991, 965, 801, 764, 687. ^1^H-NMR (600 MHz, CDCl_3_): δ = 8.46 (brs, 1H), 8.25–8.23 (m, 1H), 8.22–8.20 (m, 1H), 7.94–7.89 (m, 2H), 7.86–7.83 (m, 1H), 7.63–7.60 (m, 1H), 7.57–7.55 (m, 1H), 6.94 (dd, ^3^*J* = 17.2 Hz, ^3^*J* = 10.4 Hz, 1H, C*H*=CH_2_), 6.71 (dd, ^3^*J* = 17.2 Hz, ^2^*J* = 1.6 Hz, 1H, CH=C*H*_2_), 5.82 (dd, ^3^*J* = 10.4 Hz, ^2^*J* = 1.6 Hz, 1H, CH=C*H*_2_), 5.80 (s, 2H, N-CH_2_). ^13^C-NMR (151 MHz, CDCl_3_): δ = 165.61 (s, C(O)), 159.72, 151.74, 148.48, 138.58, 136.98, 133.89, 133.82, 129.61, 127.82, 126.88, 123.89, 123.33, 123.15, 122.98, 115.24, 66.83 (s, N-CH_2_). Anal. Calcd. for C_17_H_13_N_3_O_3_: C, 66.44; H, 4.26; N, 13.67. Found: C, 66.12; H, 3.97; N, 13.40.

*3-(4-Nitrobenzyl)-2-vinylquinazolin-4(3H)-one* (**13e**). Yellowish amorphous solid, m.p. = 131 °C–132 °C. IR (KBr, cm^−1^) ν_max_: 2944, 2849, 1608, 1570, 1514, 1488, 1338, 1162, 984, 854, 812, 704, 680. ^1^H-NMR (600 MHz, CDCl_3_): δ = 8.30–8.28 (m, 2H), 8.24–8.21 (m, 1H), 7.96–7.94 (m, 1H), 7.87–7.84 (m, 1H), 7.73–7.72 (m, 2H), 7.58–7.55 (m, 1H), 6.93 (dd, ^3^*J* = 17.2 Hz, ^3^*J* = 10.4 Hz, 1H, C*H*=CH_2_), 6.68 (dd, ^3^*J* = 17.2 Hz, ^2^*J* = 1.8 Hz, 1H, CH=C*H*_2_), 5.82 (s, 2H, N-CH_2_), 5.80 (dd, ^3^*J* = 10.4 Hz, ^2^*J* = 1.6 Hz, 1H, CH=C*H*_2_). ^13^C-NMR (151 MHz, CDCl_3_): δ = 165.59 (s, C(O)), 159.74, 151.78, 147.77, 143.81, 136.97, 133.87, 128.31, 127.89, 126.91 123.91, 123.85, 123.27, 115.25, 66.78 (s, N-CH_2_). Anal. Calcd. for C_17_H_13_N_3_O_3_: C, 66.44; H, 4.26; N, 13.67. Found: C, 66.23; H, 4.03; N, 13.33.

*3-(2-Fluorobenzyl)-2-vinylquinazolin-4(3H)-one* (**13f**). Colorless oil; IR (film, cm^−1^) ν_max_: 3061, 1676, 1618, 1496, 1422, 1349, 1104, 988, 941, 759, 680, 661. ^1^H-NMR (200 MHz, CDCl_3_): δ = 8.17–8.13 (m, 1H), 7.92–7.74 (m, 2H), 7.61–7.45 (m, 2H), 7.41–7.29 (m, 1H), 7.26–7.08 (m, 2H), 6.93 (dd, ^3^*J* = 17.2 Hz, ^3^*J* = 10.0 Hz, 1H, C*H*=CH_2_), 6.74 (dd, ^3^*J* = 17.2 Hz, ^2^*J* = 2.4 Hz, 1H, CH=C*H*_2_), 5.79 (dd, ^3^*J* = 10.0 Hz, ^2^*J* = 2.4 Hz, 1H, CH=C*H*_2_), 5.76 (s, 2H, N-CH_2_). ^13^C-NMR (151 MHz, CDCl_3_): δ = 165.95 (s, C(O)), 161.15 (d, ^1^*J*_(CF)_ = 249.1 Hz, C2), 159.93, 151.69, 137.12, 133.57, 130.54 (d, ^3^*J*_(CCCF)_ = 3.5 Hz, C4), 130.13 (d, ^3^*J*_(CCCF)_ = 8.5 Hz, C6), 127.71, 126.61, 124.17 (d, ^4^*J*_(CCCCF)_ = 3.4 Hz, C5), 123.85, 123.66 (d, ^2^*J*_(CCF)_ = 14.4 Hz, C3), 123.56, 115.54 (d, ^2^*J*_(CCF)_ = 21.1 Hz, C1), 115.51, 62.16 (d, ^3^*J*_(CCCF)_ = 4.4 Hz, N-CH_2_). Anal. Calcd. for C_17_H_13_FN_2_O: C, 72.85; H, 4.67; N, 9.99. Found: C, 72.78; H, 4.41; N, 9.80.

*3-(3-Fluorobenzyl)-2-vinylquinazolin-4(3H)-one* (**13g**). White amorphous solid, m.p. = 58 °C–59 °C. IR (KBr, cm^−1^) ν_max_: 3095, 3057, 3032, 1618, 1578, 1498, 1422, 1349, 1106, 988, 945, 681, 520. ^1^H-NMR (200 MHz, CDCl_3_): δ = 8.20–8.15 (m, 1H), 7.93–7.88 (m, 1H), 7.84–7.75 (m, 1H), 7.55–7.47 (m, 1H), 7.39–7.22 (m, 3H), 7.10–7.01 (m, 1H), 6.93 (dd, ^3^*J* = 17.2 Hz, ^3^*J* = 10.1 Hz, 1H, C*H*=CH_2_), 6.70 (dd, ^3^*J* = 17.2 Hz, ^2^*J* = 2.3 Hz, 1H, CH=C*H*_2_), 5.79 (dd, ^3^*J* = 10.1 Hz, ^2^*J* = 2.3 Hz, 1H, CH=C*H*_2_), 5.67 (s, 2H, N-CH_2_). ^13^C-NMR (151 MHz, CDCl_3_): δ = 165.88 (s, C(O)), 162.95 (d, ^1^*J*_(CF)_ = 246.6 Hz, C3), 159.88, 151.69, 138.97 (d, ^3^*J*_(CCCF)_ = 7.8 Hz, C5), 137.09, 133.67, 130.15 (d, ^3^*J*_(CCCF)_ = 8.8 Hz, C1), 127.75, 126.72, 123.84, 123.53 (d, ^4^*J*_(CCCCF)_ = 2.4 Hz, C6), 123.48, 115.45, 115.09 (d, ^2^*J*_(CCF)_ = 21.0 Hz, C4), 114.93 (d, ^2^*J*_(CCF)_ = 22.0 Hz, C2), 67.39 (s, N-CH_2_). Anal. Calcd. for C_17_H_13_FN_2_O: C, 72.85; H, 4.67; N, 9.99. Found: C, 72.66; H, 4.38; N, 9.95.

*3-(4-Fluorobenzyl)-2-vinylquinazolin-4(3H)-one* (**13h**). White amorphous solid, m.p. = 125 °C–127 °C. IR (KBr, cm^−1^) ν_max_: 3058, 1677, 1618, 1493, 1425, 1349, 1102, 992, 942, 757, 683, 658. ^1^H-NMR (200 MHz, CDCl_3_): δ = 8.12–8.07 (m, 1H), 7.87–7.82 (m, 1H), 7.78–7.73 (m, 1H), 7.51–7.40 (m, 3H), 7.10–6.97 (m, 2H), 6.88 (dd, ^3^*J* = 17.2 Hz, ^3^*J* = 10.1 Hz, 1H, C*H*=CH_2_), 6.66 (dd, ^3^*J* = 17.2 Hz, ^2^*J* = 2.3 Hz, 1H, CH=C*H*_2_), 5.74 (dd, ^3^*J* = 10.1 Hz, ^2^*J* = 2.3 Hz, 1H, CH=C*H*_2_), 5.59 (s, 2H, N-CH_2_). ^13^C-NMR (151 MHz, CDCl_3_): δ = 165.99 (s, C(O)), 162.71 (d, ^1^*J*_(CF)_ = 249.1 Hz, C4), 159.90, 151.69, 137.19, 133.59, 132.26 (d, ^4^*J*_(CCCCF)_ = 3.2 Hz, C1), 130.22 (d, ^3^*J*_(CCCF)_ = 7.7 Hz, C2, C6), 127.74, 126.62, 123.70, 123.49, 115.55 (d, ^2^*J*_(CCF)_ = 21.3 Hz, C3, C5), 67.58 (s, N-CH_2_). Anal. Calcd. for C_17_H_13_FN_2_O: C, 72.85; H, 4.67; N, 9.99. Found: C, 72.67; H, 4.30; N, 9.70.

*3-(2,4-Difluorobenzyl)-2-vinylquinazolin-4(3H)-one* (**13i**). Yellowish amorphous solid, m.p. = 67 °C–68 °C. IR (KBr, cm^−1^) ν_max_: 3076, 3015, 2929, 1681, 1606, 1574, 1505, 1351, 1279, 1100, 964, 851, 772, 666, 683. ^1^H-NMR (200 MHz, CDCl_3_): δ = 8.10–8.05 (m, 1H), 7.86–7.82 (m, 1H), 7.78–7.68 (m, 1H), 7.57–7.40 (m, 2H), 6.90–6.78 (m, 2H), 6.88 (dd, ^3^*J* = 17.2 Hz, ^3^*J* = 10.1 Hz, 1H, C*H*=CH_2_), 6.67 (dd, ^3^*J* = 17.2 Hz, ^2^*J* = 2.3 Hz, 1H, CH=C*H*_2_), 5.74 (dd, ^3^*J* = 10.1 Hz, ^2^*J* = 2.3 Hz, 1H, CH=C*H*_2_), 5.65 (s, 2H, N-CH_2_). ^13^C-NMR (151 MHz, CDCl_3_): δ = 165.84 (s, C(O)), 163.11 (dd, ^1^*J*_(CF)_ = 249.9 Hz, ^3^*J*_(CCCF)_ = 12.1 Hz, C2) 161.40 (dd, ^1^*J*_(CF)_ = 251.2 Hz, ^3^*J*_(CCCF)_ = 12.1 Hz, C4), 159.85, 151.69, 137.11, 133.64, 131.70 (dd, ^3^*J*_(CCCF)_ = 9.8 Hz, ^3^*J*_(CCCF)_ = 5.5 Hz, C6), 127.75, 126.66, 123.81, 123.46, 119.69 (dd, ^2^*J*_(CCF)_ = 14.6 Hz, ^4^*J*_(CCCCF)_ = 3.6 Hz, C1), 115.44, 111.40 (dd, ^2^*J*_(CCF)_ = 21.0 Hz, ^4^*J*_(CCCCF)_ = 3.4 Hz, C5), 104.07 (dd, ^2^*J*_(CCF)_ = 25.3 Hz, ^2^*J*_(CCF)_ = 25.0 Hz, C3), 61.61 (d, ^3^*J*_(CCCF)_ = 4.0 Hz, N-CH_2_). Anal. Calcd. for C_17_H_12_F_2_N_2_O: C, 68.45; H, 4.06; N, 9.39. Found: C, 68.19; H, 3.87; N, 9.45.

### 3.3. General Procedure for the Synthesis of N3-Alkylated 2-Vinyl-3H-quinazolin-4-ones ***13j*** and ***13k***

To the solution of 2-vinyl-3*H*-quinazolin-4-one (**13a**, 1.00 mmol) in acetonitrile (15 mL) potassium carbonate (3.00 mmol) was added. After 15 min. iodomethane (2.00 mmol) or iodoethane (1.10 mmol) was added and the reaction mixture was stirred at 60 °C for 5 h. The solvent was removed and a residue was extracted with water (3 × 10 mL). Organic layer was dried (MgSO_4_), concentrated and the crude product was purified on a silica gel column with methylene chloride:hexane mixture (7:3, *v*/*v*) followed by crystallization (chloroform : petroleum ether) to give pure quinazolinones **13j** [35] or **13k**.

*3-Methyl-2-vinylquinazolin-4(3H)-one* (**13j**). Amorphous solid, m.p. = 122 °C–124 °C (reference [35] m.p. = 123 °C–125 °C).

*3-Ethyl-2-vinylquinazolin-4(3H)-one* (**13k**). Yellowish oil; IR (film, cm^−1^) ν_max_: 3066, 2979, 2927, 2855, 1684, 1621, 1576, 1423, 1381, 1163, 962, 768, 681. ^1^H-NMR (200 MHz, CDCl_3_): δ = 8.16–8.11 (m, 1H), 7.90–7.85 (m, 1H), 7.81–7.73 (m, 1H), 7.52–7.44 (m, 1H), 6.91 (dd, ^3^*J* = 17.2 Hz, ^3^*J* = 10.1 Hz, 1H, C*H*=CH_2_), 6.69 (dd, ^3^*J* = 17.2 Hz, ^2^*J* = 2.3 Hz, 1H, CH=C*H*_2_), 5.76 (dd, ^3^*J* = 10.1 Hz, ^2^*J* = 2.3 Hz, 1H, CH=C*H*_2_), 4.68 (q, ^3^*J* = 7.1 Hz, 2H, C*H*_2_CH_3_), 1.52 (t, ^3^*J* = 7.1 Hz, 3H, CH_2_C*H*_3_). ^13^C-NMR (151 MHz, CDCl_3_): δ = 166.31 (s, C(O)), 160.09, 151.51, 137.29, 133.35, 127.62, 126.39, 123.55, 115.66, 62.70 (s, *C*H_2_CH_3_), 14.34 (s, CH_2_*C*H_3_). Anal. Calcd. for C_12_H_12_N_2_O × 0.25 H_2_O: C, 70.40; H, 6.15; N, 13.68. Found: C, 70.73; H, 5.92; N, 13.80.

### 3.4. General Procedure for the Synthesis of Isoxazolidines Trans-**11** and Cis-**11**

A solution of the nitrone **12** (1.0 mmol) and the respective vinyl quinazolinone (1.0 mmol) in toluene (2 mL) was stirred at 70 °C until the disappearance (TLC) of the starting nitrone. All volatiles were removed *in vacuo* and crude products were subjected to chromatography on silica gel columns with a chloroform/methanol (100:1, 50:1, 20:1, *v*/*v*) mixtures as eluents.

*Diethyl trans-(2-methyl-5-(4-oxo-3,4-dihydroquinazolin-2-yl)isoxazolidin-3-yl)phosphonate* (*trans-***11a**). Yellowish oil; IR (film, cm^−1^) ν_max_: 3085, 2980, 2929, 2782, 1687, 1610, 1469, 1331, 1132, 1098, 1052, 968, 775. ^1^H-NMR (600 MHz, CDCl_3_): δ = 10.13 (s, 1H, NH), 8.31–8.29 (m, 1H), 7.80–7.78 (m, 1H), 7.71–7.69 (m, 1H), 7.53–7.50 (m, 1H), 5.04 (dd, ^3^*J*_(H5–H4β)_ = 8.3 Hz, ^3^*J*_(H5–H4α)_ = 6.2 Hz, 1H, *H*C5), 4.29–4.20 (m, 4H, 2 × CH_2_OP), 3.28–3.24 (m, 1H, HC3), 3.13 (dddd, ^3^*J*_(H4β–P)_ = 16.9 Hz, ^2^*J*_(H4β–H4α)_ = 12.8 Hz, ^3^*J*_(H4β–H3)_ = 8.3 Hz, ^3^*J*_(H4β–H5)_ = 8.3 Hz, 1H, H_β_C4), 3.05 (s, 3H, CH_3_N), 2.96 (dddd, ^2^*J*_(H4α–H4β)_ = 12.8 Hz, ^3^*J*_(H4α–P)_ = 9.9 Hz, ^3^*J*_(H4α–H3)_ = 9.3 Hz, ^3^*J*_(H4α–H5)_ = 6.2 Hz, 1H, H_α_C4), 1.40 (t, ^3^*J* = 7.1 Hz, 3H, C*H*_3_CH_2_OP), 1.38 (t, ^3^*J* = 7.2 Hz, 3H, C*H*_3_CH_2_OP). ^13^C-NMR (151 MHz, CDCl_3_): δ = 161.75 (s, C(O)), 153.76, 148.61, 134.82, 127.45, 127.15, 126.60, 121.61, 75.82 (d, ^3^*J*_(CCCP)_ = 8.5 Hz, C5), 63.76 (d, ^1^*J*_(CP)_ = 170.0 Hz, C3), 63.36 (d, ^2^*J*_(COP)_ = 6.5 Hz, CH_2_OP), 62.59 (d, ^2^*J*_(COP)_ = 7.3 Hz, CH_2_OP), 46.33 (s, CH_3_N), 37.77 (d, ^2^*J*_(CCP)_ = 1.9 Hz, C4), 16.53 (d, ^3^*J*_(CCOP)_ = 5.7 Hz, *C*H_3_CH_2_OP), 16.46 (d, ^3^*J*_(CCOP)_ = 5.6 Hz, *C*H_3_CH_2_OP). ^31^P-NMR (243 MHz, CDCl_3_): δ = 20.64. Anal. Calcd. for C_16_H_22_N_3_O_5_P × 0.25 H_2_O: C, 51.68; H, 6.10; N, 11.30. Found: C, 51.63; H, 6.09; N, 11.32.

*Diethyl cis-(2-methyl-5-(4-oxo-3,4-dihydroquinazolin-2-yl)isoxazolidin-3-yl)phosphonate* (*cis*-**11a**). Data noted below correspond to a 17:83 mixture of *trans*-**11a** and *cis*-**11a**. Yellowish oil; IR (film, cm^−1^) ν_max_: 3202, 3086, 2980, 2926, 2791, 1688, 1610, 1567, 1469, 1392, 1330, 1231, 1163, 1100, 1024, 973, 776, 573. (signals of *cis*-**11a** were extracted from the spectra of a 17:83 mixture of *trans*-**11a** and *cis*-**11a**); ^1^H-NMR (600 MHz, CDCl_3_): δ = 10.48 (s, 1H, NH), 8.31–8.29 (m, 1H), 7.77–7.75 (m, 1H), 7.67–7.65 (m, 1H), 7.50–7.47 (m, 1H), 5.10 (dd, ^3^*J*_(H5–H4α)_ = 9.1 Hz, ^3^*J*_(H5–H4β)_ = 3.9 Hz, 1H, HC5), 4.27–4.11 (m, 4H, 2 × CH_2_OP), 3.21 (dddd, ^3^*J*_(H4α–P)_ = 11.2 Hz, ^2^*J*_(H4β–H4α)_ = 12.6 Hz, ^3^*J*_(H4α–H5)_ = 9.1 Hz, ^3^*J*_(H4α–H3)_ = 9.0 Hz, 1H, H_α_C4), 3.10 (ddd, ^3^*J*_(H3–H4α)_ = 9.0 Hz, ^3^*J*_(H3–H4β)_ = 6.5 Hz, ^2^*J*_(H3-P)_ = 3.9 Hz, 1H, HC3), 3.01 (s, 3H, CH_3_N), 2.85 (dddd, ^3^*J*_(H4β–P)_ = 20.0 Hz, ^2^*J*_(H4α–H4β)_ = 12.6 Hz, ^3^*J*_(H4β–H3)_ = 6.5 Hz, ^3^*J*_(H4β–H5)_ = 3.9 Hz, 1H, H_β_C4), 1.31 (t, ^3^*J* = 7.5 Hz, 3H, C*H*_3_CH_2_OP), 1.25 (t, ^3^*J* = 6.5 Hz, 3H, C*H*_3_CH_2_OP). ^13^C-NMR (151 MHz, CDCl_3_): δ = 161.52 (s, C(O)), 156.11, 148.53, 134.53, 127.14, 126.88, 126.67, 121.83, 75.51 (d, ^3^*J*_(CCCP)_ = 7.3 Hz, C5), 63.66 (d, ^1^*J*_(CP)_ = 168.5 Hz, C3), 63.23 (d, ^2^*J*_(COP)_ = 6.6 Hz, CH_2_OP), 63.03 (d, ^2^*J*_(COP)_ = 6.7 Hz, CH_2_OP), 45.62 (d, ^3^*J*_(CNCP)_ = 5.4 Hz, CH_3_N), 37.96 (s, C4), 16.38 (d, ^3^*J*_(CCOP)_ = 5.5 Hz, *C*H_3_CH_2_OP), 16.35 (d, ^3^*J*_(CCOP)_ = 5.5 Hz, *C*H_3_CH_2_OP). ^31^P-NMR (243 MHz, CDCl_3_): δ = 20.87. Anal. Calcd. for C_16_H_22_N_3_O_5_P × 0.25 H_2_O: C, 51.68; H, 6.10; N, 11.30. Found: C, 51.63; H, 6.05; N, 11.32 (obtained on a 90:10 mixture of *trans*-**11a** and *cis*-**11a**).

*Diethyl trans-(5-(3-benzyl-4-oxo-3,4-dihydroquinazolin-2-yl)-2-methylisoxazolidin-3-yl)phosphonate* (*trans*-**11b**). Yellowish oil; IR (film, cm^−1^) ν_max_: 3065, 3033, 2981, 2915, 2779, 1687, 1620, 1574, 1498, 1455, 1347, 1239, 1104, 1026, 966, 774, 683. ^1^H-NMR (600 MHz, CDCl_3_): δ = 8.22–8.20 (m, 1H), 7.97–7.95 (m, 1H), 7.84–7.82 (m, 1H), 7.57–7.54 (m, 1H), 7.43–7.41 (m, 1H), 7.38–7.36 (m, 1H), 5.67 (s, 2H, N-CH_2_), 5.28 (dd, ^3^*J*_(H5–H4β)_ = 7.4 Hz, ^3^*J*_(H5–H4α)_ = 6.5 Hz, 1H, HC5), 4.33–4.20 (m, 4H, 2 × CH_2_OP), 3.45–3.42 (m, 1H, HC3), 3.06 (s, 3H, CH_3_N), 3.07–2.97 (m, 2H, H_α_C4, H_β_C4), 1.42 (t, ^3^*J* = 7.0 Hz, 3H, C*H*_3_CH_2_OP), 1.39 (t, ^3^*J* = 7.1 Hz, 3H, C*H*_3_CH_2_OP). ^13^C-NMR (151 MHz, CDCl_3_): δ = 167.04 (s, C(O)), 162.94, 151.27, 136.14, 133.71, 128.59, 128.30, 128.24, 127.86, 127.12, 123.50, 115.60, 80.30 (d, ^3^*J*_(CCCP)_ = 8.6 Hz, C5), 68.73 (s, CH_2_N), 64.44 (d, ^1^*J*_(CP)_ = 168.2 Hz, C3), 63.25 (d, ^2^*J*_(COP)_ = 6.5 Hz, CH_2_OP), 62.55 (d, ^2^*J*_(COP)_ = 6.9 Hz, CH_2_OP), 46.63 (s, CH_3_N), 37.95 (s, C4), 16.56 (d, ^3^*J*_(CCOP)_ = 5.6 Hz, *C*H_3_CH_2_OP), 16.51 (d, ^3^*J*_(CCOP)_ = 5.6 Hz, *C*H_3_CH_2_OP). ^31^P-NMR (243 MHz, CDCl_3_): δ = 22.19. Anal. Calcd. for C_23_H_28_N_3_O_5_P: C, 60.39; H, 6.17; N, 9.19. Found: C, 59.99; H, 6.21; N, 9.15.

*Diethyl trans-(2-methyl-5-(3-(2-nitrobenzyl)-4-oxo-3,4-dihydroquinazolin-2-yl)isoxazolidin-3-yl)-phosphonate* (*trans*-**11c**). Yellowish oil; IR (film, cm^−1^) ν_max_: 3067, 2973, 2918, 1620, 1576, 1526, 1347, 1337, 1264, 1050, 1018, 964, 790, 733, 584, 571. ^1^H-NMR (200 MHz, CDCl_3_): δ = 8.18–8.07 (m, 2H), 7.93–7.69 (m, 3H), 7.60–7.42 (m, 3H), 6.01 (s, 2H, N-CH_2_), 5.15 (dd, ^3^*J*_(H5–H4β)_ = 7.8 Hz, ^3^*J*_(H5–H4α)_ = 6.1 Hz, 1H, HC5), 4.29–4.07 (m, 4H, 2 × CH_2_OP), 3.28 (ddd, ^3^*J*_(H3–H4α)_ = 10.6 Hz, ^3^*J*_(H3–H4β)_ = 8.5 Hz, ^2^*J*_(H3-P)_ = 1.2 Hz, 1H, HC3), 2.93 (s, 3H, CH_3_N), 2.99–2.82 (m, 1H, H_β_C4), 2.76 (dddd, ^3^*J*_(H4α–P)_ = 12.5 Hz, ^2^*J*_(H4α–H4β)_ = 12.5 Hz, ^3^*J*_(H4α–H3)_ = 10.6 Hz, ^3^*J*_(H4α–H5)_ = 6.1 Hz, 1H, H_α_C4), 1.33 (t, ^3^*J* = 7.0 Hz, 3H, C*H*_3_CH_2_OP), 1.31 (t, ^3^*J* = 7.0 Hz, 3H, C*H*_3_CH_2_OP). ^13^C-NMR (151 MHz, CDCl_3_): δ = 166.51 (s, C(O)), 162.99, 151.40, 147.85, 133.98, 133.68, 132.46, 129.10, 128.82, 128.08, 127.44, 125.06, 123.19, 115.30, 80.23 (d, ^3^*J*_(CCCP)_ = 8.4 Hz, C5), 65.34 (s, N-CH_2_), 64.33 (d, ^1^*J*_(CP)_ = 168.4 Hz, C3), 63.19 (d, ^2^*J*_(COP)_ = 6.5 Hz, CH_2_OP), 62.44 (d, ^2^*J*_(COP)_ = 7.1 Hz, CH_2_OP), 46.58 (d, ^3^*J*_(CNCP)_ = 3.8 Hz, CH_3_N), 37.98 (s, C4), 16.55 (d, ^3^*J*_(CCOP)_ = 6.4 Hz, *C*H_3_CH_2_OP), 16.49 (d, ^3^*J*_(CCOP)_ = 5.7 Hz, *C*H_3_CH_2_OP). ^31^P-NMR (81 MHz, CDCl_3_): δ = 22.85. Anal. Calcd. for C_23_H_27_N_4_O_7_P × 0.25 H_2_O: C, 54.49; H, 5.47; N, 11.05. Found: C, 54.42; H, 5.28; N, 10.89.

*Diethyl trans-(2-methyl-5-(3-(3-nitrobenzyl)-4-oxo-3,4-dihydroquinazolin-2-yl)isoxazolidin-3-yl)-phosphonate* (*trans*-**11d**). Data noted below correspond to a 92:8 mixture of *trans*-**11d** and *cis*-**11d**. A yellowish oil; IR (film, cm^−1^) ν_max_: 3070, 2982, 2930, 2910, 1620, 1574, 1531, 1497, 1415, 1298, 1103, 1025, 774, 733, 682. (signals of *trans*-**11d** were extracted from the spectra of a 92:8 mixture of *trans*-**11d** and *cis*-**11d**); ^1^H-NMR (600 MHz, CDCl_3_): δ = 8.46–8.44 (m, 1H), 8.25–8.22 (m, 2H), 8.00–7.98 (m, 1H), 7.91–7.88 (m, 2H), 7.63–7.60 (m, 2H), 5.78 (s, 2H, N-CH_2_), 5.28 (dd, ^3^*J*_(H5–H4β)_ = 6.8 Hz, ^3^*J*_(H5–H4α)_ = 6.8 Hz, 1H, HC5), 4.33–4.20 (m, 4H, 2 × CH_2_OP), 3.40–3.38 (m, 1H, HC3), 3.07–3.00 (m, 1H, *H*_β_C4), 3.05 (s, 3H, CH_3_N), 2.98 (dddd, ^3^*J*_(H4α–P)_ = 12.2 Hz, ^2^*J*_(H4α–H4β)_ = 12.2 Hz, ^3^*J*_(H4α–H3)_ = 9.8 Hz, ^3^*J*_(H4α–H5)_ = 6.8 Hz, 1H, H_α_C4), 1.42 (t, ^3^*J* = 7.1 Hz, 3H, C*H*_3_CH_2_OP), 1.40 (t, ^3^*J* = 6.7 Hz, 3H, C*H*_3_CH_2_OP). ^13^C-NMR (151 MHz, CDCl_3_): δ = 166.54 (s, C(O)), 162.63, 151.38, 148.50, 138.29, 134.06, 133.97, 129.65, 128.02, 127.45, 123.25, 123.00, 115.30, 80.14 (d, ^3^*J*_(CCCP)_ = 8.4 Hz, C5), 67.21 (s, N-CH_2_), 64.42 (d, ^1^*J*_(CP)_ = 168.5 Hz, C3), 63.24 (d, ^2^*J*_(COP)_ = 6.5 Hz, CH_2_OP), 62.44 (d, ^2^*J*_(COP)_ = 7.2 Hz, CH_2_OP), 46.33 (s, CH_3_N), 37.84 (s, C4), 16.56 (d, ^3^*J*_(CCOP)_ = 5.6 Hz, *C*H_3_CH_2_OP), 16.46 (d, ^3^*J*_(CCOP)_ = 4.2 Hz, *C*H_3_CH_2_OP). ^31^P-NMR (243 MHz, CDCl_3_): δ = 22.07. Anal. Calcd. for C_23_H_27_N_4_O_7_P: C, 54.98; H, 5.42; N, 11.15. Found: C, 54.62; H, 5.24; N, 11.14 (obtained on a 92:8 mixture of *trans*-**11d** and *cis*-**11d**).

*Diethyl trans-(2-methyl-5-(3-(4-nitrobenzyl)-4-oxo-3,4-dihydroquinazolin-2-yl)isoxazolidin-3-yl)-phosphonate* (*trans*-**11e**). Data noted below correspond to a 90:10 mixture of *trans*-**11e** and *cis*-**11e**. A yellowish oil; IR (film, cm^−1^) ν_max_: 3073, 2982, 2929, 2781, 1687, 1608, 1523, 1498, 1342, 1106, 968, 851, 739. (signals of *trans*-**11e** were extracted from the spectra of a 90:10 mixture of *trans*-**11e** and *cis*-**11e**); ^1^H-NMR (600 MHz, CDCl_3_): δ = 8.29–8.27 (m, 2H), 8.23–8.21 (m, 1H), 8.00–7.98 (m, 1H), 7.89–7.86 (m, 1H), 7.72–7.70 (m, 2H), 7.62–7.60 (m, 1H), 5.77 (s, 2H, N-CH_2_), 5.26 (dd, ^3^*J*_(H5–H4β)_ = 6.8 Hz, ^3^*J*_(H5–H4α)_ = 6.8 Hz, 1H, HC5), 4.33–4.19 (m, 4H, 2 × CH_2_OP), 3.38–3.35 (m, 1H, HC3), 3.05 (s, 3H, CH_3_N), 3.04–2.91 (m, 2H, H_α_C4, H_β_C4), 1.41 (t, ^3^*J* = 7.2 Hz, 3H, C*H*_3_CH_2_OP), 1.39 (t, ^3^*J* = 7.2 Hz, 3H, C*H*_3_CH_2_OP). ^13^C-NMR (151 MHz, CDCl_3_): δ = 166.51 (s, C(O)), 162.65, 151.35, 147.82, 143.45, 134.04, 128.90, 128.45, 128.07, 127.48, 123.84, 123.19, 115.29, 80.10 (d, ^3^*J*_(CCCP)_ = 8.8 Hz, C5), 67.18 (s, N-*C*H_2_), 64.42 (d, ^1^*J*_(CP)_ = 168.6 Hz, C3), 63.25 (d, ^2^*J*_(COP)_ = 5.3 Hz, CH_2_OP), 62.46 (d, ^2^*J*_(COP)_ = 6.9 Hz, CH_2_OP), 46.58 (s, CH_3_N), 37.84 (s, C4), 16.55 (d, ^3^*J*_(CCOP)_ = 5.6 Hz, *C*H_3_CH_2_OP), 16.50 (d, ^3^*J*_(CCOP)_ = 5.7 Hz, *C*H_3_CH_2_OP). ^31^P-NMR (243 MHz, CDCl_3_): δ = 22.05. Anal. Calcd. for C_23_H_27_N_4_O_7_P × 0.5 H_2_O: C, 54.01; H, 5.52; N, 10.95. Found: C, 53.91; H, 5.24; N, 10.95 (obtained on a 90:10 mixture of *trans*-**11e** and *cis*-**11e**).

*Diethyl trans-(5-(3-(2-fluorobenzyl)-4-oxo-3,4-dihydroquinazolin-2-yl)-2-methylisoxazolidin-3-yl)-phosphonate* (*trans-***11f**). Data noted below correspond to a 90:10 mixture of *trans*-**11f** and *cis*-**11f**. A colorless oil; IR (film, cm^−1^) ν_max_: 3067, 2981, 2930, 2909, 1620, 1574, 1498, 1457, 1418, 1346, 1298, 1162, 1101, 1025, 965, 770, 682. (signals of *trans*-**11f** were extracted from the spectra of a 90:10 mixture of *trans*-**11f** and *cis*-**11f**); ^1^H-NMR (600 MHz, CDCl_3_): δ = 8.17–8.15 (m, 1H), 7.95–7.93 (m, 1H), 7.83–7.80 (m, 1H), 7.58–7.52 (m, 2H), 7.36–7.32 (m, 1H), 7.17–7.15 (m, 1H), 7.13–7.10 (m, 1H), 5.73 (AB, ^2^*J*_AB_ = 12.6 Hz, 1H, N-CH_2b_), 5.70 (AB, ^2^*J*_AB_ = 12.6 Hz, 1H, N-CH_2a_), 5.26 (dd, ^3^*J*_(H5–H4β)_ = 7.3 Hz, ^3^*J*_(H5–H4α)_ = 6.7 Hz, 1H, HC5), 4.32–4.18 (m, 4H, 2 × CH_2_OP), 3.43–3.40 (m, 1H, HC3), 3.04 (s, 3H, CH_3_N), 3.03–2.93 (m, 2H, H_α_C4, H_β_C4), 1.40 (t, ^3^*J* = 7.0 Hz, 3H, C*H*_3_CH_2_OP), 1.38 (t, ^3^*J* = 7.1 Hz, 3H, C*H*_3_CH_2_OP). ^13^C-NMR (151.0 MHz, CDCl_3_): δ = 166.86 (s, C(O)), 162.84, 161.08 (d, ^1^*J*_(CF)_ = 248.6 Hz, C2’), 151.19, 133.76, 130.60 (d, ^3^*J*_(CCCF)_ = 3.4 Hz, C4’), 130.26 (d, ^3^*J*_(CCCF)_ = 8.6 Hz, C6’), 127.85, 127.17, 124.17 (d, ^4^*J*_(CCCCF)_ = 3.3 Hz, C5’), 123.45, 123.31 (d, ^2^*J*_(CCF)_ = 14.3 Hz, C3’), 115.54 (d, ^2^*J*_(CCF)_ = 21.4 Hz, C1’), 115.49, 80.27 (dd, ^3^*J*_(CCCP)_ = 8.7 Hz, C5), 64.41 (d, ^1^*J*_(CP)_ = 168.3 Hz, C3), 63.23 (d, ^2^*J*_(COP)_ = 6.3 Hz, CH_2_OP), 62.60 (d, ^3^*J* = 4.3 Hz, CH_2_N), 62.39 (d, ^2^*J*_(COP)_ = 6.8 Hz, CH_2_OP), 46.61 (s, CH_3_N), 37.94 (s, C4), 16.56 (d, ^3^*J*_(CCOP)_ = 5.6 Hz, *C*H_3_CH_2_OP), 16.50 (d, ^3^*J*_(CCOP)_ = 5.6 Hz, *C*H_3_CH_2_OP). ^31^P-NMR (243 MHz, CDCl_3_): δ = 22.16. Anal. Calcd. for C_23_H_27_FN_3_O_5_P × 0.5 H_2_O: C, 57.02; H, 5.83; N, 8.67. Found: C, 57.30; H, 5.58; N, 8.62 (obtained on a 90:10 mixture of *trans*-**11f** and *cis*-**11f**).

*Diethyl trans-(5-(3-(3-fluorobenzyl)-4-oxo-3,4-dihydroquinazolin-2-yl)-2-methylisoxazolidin-3-yl)-phosphonate* (*trans*-**11g**). Colorless oil; IR (film, cm^−1^) ν_max_: 3066, 2982, 2909, 1620, 1575, 1497, 1452, 1416, 1343, 1299, 1163, 1104, 966, 775, 682. ^1^H-NMR (600 MHz, CDCl_3_): δ = 8.22–8.20 (m, 1H), 7.98–7.96 (m, 1H), 7.86–7.84 (m, 1H), 7.59–7.57 (m, 1H), 7.40–7.37 (m, 1H), 7.31–7.28 (m, 2H), 7.08–7.06 (m, 1H), 5.66 (s, 2H, N-C*H*_2_), 5.27 (dd, ^3^*J*_(H5–H4β)_ = 7.4 Hz, ^3^*J*_(H5–H4α)_ = 6.7 Hz, 1H, HC5), 4.33–4.21 (m, 4H, 2 × CH_2_OP), 3.42–3.40 (m,1H, HC3), 3.05 (s, 3H, CH_3_N), 3.04–2.94 (m, 2H, H_α_C4, H_β_C4) 1.42 (t, ^3^*J* = 7.1 Hz, 3H, C*H*_3_CH_2_OP), 1.39 (t, ^3^*J* = 7.0 Hz, 3H, C*H*_3_CH_2_OP). ^13^C-NMR (151 MHz, CDCl_3_): δ = 166.80 (s, C(O)), 162.92 (d, ^1^*J*_(CF)_ = 246.6 Hz, C3’), 162.80, 151.25, 138.65 (d, ^3^*J*_(CCCF)_ = 7.6 Hz, C5’), 133.84, 130.17 (d, *J* = 8.7 Hz, C1’), 127.94, 127.27, 123.60 (d, ^4^*J*_(CCCCF)_ = 2.6 Hz, C6’), 123.39, 115.47, 115.18 (d, ^2^*J*_(CCF)_ = 21.0 Hz, C4’), 115.00 (d, ^2^*J*_(CCF)_ = 22.0 Hz, C2’), 80.22 (dd, ^3^*J*_(CCCP)_ = 7.9 Hz, C5), 67.80 (d, ^3^*J* = 1.6 Hz, CH_2_N), 64.43 (d, ^1^*J*_(CP)_ = 168.3 Hz, C3), 63.26 (d, ^2^*J*_(COP)_ = 6.7 Hz, CH_2_OP), 62.41 (d, ^2^*J*_(COP)_ = 6.4 Hz, CH_2_OP), 46.59 (s, CH_3_N), 37.92 (s, C4), 16.56 (d, ^3^*J*_(CCOP)_ = 5.6 Hz, *C*H_3_CH_2_OP), 16.50 (d, ^3^*J*_(CCOP)_ = 5.6 Hz, *C*H_3_CH_2_OP). ^31^P-NMR (243 MHz, CDCl_3_): δ = 22.14. Anal. Calcd. for C_23_H_27_FN_3_O_5_P × 0.5 H_2_O: C, 57.02; H, 5.83; N, 8.67. Found: C, 57.30; H, 5.66; N, 8.74.

*Diethyl trans-(5-(3-(4-fluorobenzyl)-4-oxo-3,4-dihydroquinazolin-2-yl)-2-methylisoxazolidin-3-yl)-phosphonate* (*trans*-**11h**). Colorless oil; IR (film, cm^−1^) ν_max_: 3069, 2982, 2930, 2910, 1620, 1574, 1512, 1498, 1226, 1104, 966, 823, 774, 683. ^1^H-NMR (600 MHz, CDCl_3_): δ = 8.17–8.15 (m, 1H), 7.96–7.94 (m, 1H), 7.84–7.81 (m, 1H), 7.56–7.51 (m, 3H), 7.11–7.08 (m, 2H), 5.62 (s, 2H, N-CH_2_), 5.27 (dd, ^3^*J*_(H5–H4β)_ = 6.7 Hz, ^3^*J*_(H5–H4α)_ = 6.7 Hz, 1H, HC5), 4.32–4.20 (m, 4H, 2 × CH_2_OP), 3.43–3.40 (m, 1H, HC3), 3.07–2.99 (m, 1H, H_β_C4), 3.06 (s, 3H, CH_3_N), 2.99 (dddd, ^3^*J*_(H4α–P)_ = 12.5 Hz, ^2^*J*_(H4α–H4β)_ = 12.5 Hz, ^3^*J*_(H4α–H3)_ = 10.3 Hz, ^3^*J*_(H4α–H5)_ = 6.7 Hz, 1H, H_α_C4), 1.41 (t, ^3^*J* = 7.1 Hz, 3H, C*H*_3_CH_2_OP), 1.39 (t, ^3^*J* = 6.4 Hz, 3H, C*H*_3_CH_2_OP). ^13^C-NMR (151 MHz, CDCl_3_): δ = 166. 90 (s, C(O)), 162.84, 162.73 (d, ^1^*J*_(CF)_ = 246.9 Hz, C4’), 151.21, 133.76, 131.93 (d, ^4^*J*_(CCCCF)_ = 3.2 Hz, C1’), 130.28 (d, ^3^*J*_(CCCF)_ = 8.0 Hz, C2’, C6’), 127.89, 127.18, 123.41, 115.53 (d, ^2^*J*_(CCF)_ = 21.4 Hz, C3’, C5’), 115.52, 80.23 (d, ^3^*J*_(CCP)_ = 8.1 Hz, C5), 68.02 (s, N-CH_2_), 64.45 (d, ^1^*J*_(CP)_ = 168.1 Hz, C3), 63.25 (d, ^2^*J*_(COP)_ = 6.4 Hz, CH_2_OP), 62.39 (d, ^2^*J*_(COP)_ = 6.9 Hz, CH_2_OP), 46.62 (s, CH_3_N), 37.96 (s, C4), 16.56 (d, ^3^*J*_(CCOP)_ = 5.6 Hz, *C*H_3_CH_2_OP), 16.50 (d, ^3^*J*_(CCOP)_ = 5.5 Hz, *C*H_3_CH_2_OP). ^31^P-NMR (243 MHz, CDCl_3_): δ = 22.13. Anal. Calcd. for C_23_H_27_FN_3_O_5_P: C, 58.10; H, 5.72; N, 8.84. Found: C, 57.74; H, 5.70; N, 8.77.

*Diethyl trans-(5-(3-(2,4-difluorobenzyl)-4-oxo-3,4-dihydroquinazolin-2-yl)-2-methylisoxazolidin-3-yl)-phosphonate* (*trans*-**11i**). Data noted below correspond to a 92:8 mixture of *trans*-**11i** and *cis*-**11i**. A colorless oil; IR (film, cm^−1^) ν_max_: 3068, 2982, 2930, 2910, 1621, 1574, 1498, 1416, 1162, 1102, 1025, 961, 773, 682. (signals of *trans*-**11i** were extracted from the spectra of a 92:8 mixture of *trans*-**11i** and *cis*-**11i**); ^1^H-NMR (600 MHz, CDCl_3_): δ = 8.18–8.16 (m, 1H), 7.98–7.96 (m, 1H), 7.86–7.84 (m, 1H), 7.62–7.56 (m, 2H), 6.95–6.89 (m, 2H), 5.71 (AB, ^2^*J*_AB_ = 12.4 Hz, 1H, N-CH_2b_), 5.71 (AB, ^2^*J*_AB_ = 12.4 Hz, 1H, N-CH_2a_), 5.29 (dd, ^3^*J*_(H5–H4β)_ = 6.6 Hz, ^3^*J*_(H5–H4α)_ = 6.2 Hz, 1H, HC5), 4.34–4.22 (m, 4H, 2 × CH_2_OP), 3.44–3.41 (m, 1H, C3), 3.07 (s, 3H, CH_3_-N), 3.04–3.02 (m, 1H, H_β_C4), 3.01 (dddd, ^3^*J*_(H4α–P)_ = 12.4 Hz, ^2^*J*_(H4α–H4β)_ = 12.4 Hz, ^3^*J*_(H4α–H3)_ = 10.1 Hz, ^3^*J*_(H4α–H5)_ = 6.2 Hz, 1H, H_α_C4), 1.43 (t, ^3^*J* = 7.1 Hz, 3H, C*H*_3_CH_2_OP), 1.41 (t, ^3^*J* = 7.0 Hz, 3H, C*H*_3_CH_2_OP). ^13^C-NMR (151 MHz, CDCl_3_): δ = 165.94 (s, C(O)), 163.05 (dd, ^1^*J*_(CF)_ = 265.3 Hz, ^3^*J*_(CCCF)_ = 12.1 Hz, C2’), 162.71, 161.44 (dd, ^1^*J*_(CF)_ = 266.1 Hz, ^3^*J*_(CCCF)_ = 14.4 Hz, C4’), 151.19, 133.76, 131.87 (dd, ^3^*J*_(CCCF)_ = 9.9 Hz, ^3^*J*_(CCCF)_ = 5.5 Hz, C6’), 127.85, 127.18, 123.33, 119.34 (dd, ^2^*J*_(CCF)_ = 14.5 Hz, ^4^*J*_(CCCCF)_ = 3.3 Hz, C1’), 115.38, 111.35 (dd, ^2^*J*_(CCF)_ = 21.0 Hz, ^4^*J*_(CCCCF)_ = 3.3 Hz, C5’), 104.02 (dd, ^2^*J*_(CCF)_ = 25.3 Hz, ^2^*J*_(CCF)_ = 25.3 Hz, C3’), 80.20 (dd, ^3^*J*_(CCCP)_ = 8.7 Hz, ^3^*J*_(CCCF)_ = 2.3 Hz, C5), 64.41 (d, ^1^*J*_(CP)_ = 168.2 Hz, C3), 63.19 (d, ^2^*J*_(COP)_ = 6.1 Hz, CH_2_OP), 62.35 (d, ^2^*J*_(COP)_ = 7.3 Hz, CH_2_OP), 62.04 (d, ^3^*J*_(CCCF)_ = 3.2 Hz, CH_2_N), 46.45 (d, ^3^*J*_(CNCP)_ = 2.6 Hz, CH_3_N), 37.89 (d, ^2^*J*_(CCP)_ = 1.7 Hz, C4), 16.52 (d, ^3^*J*_(CCOP)_ = 5.5 Hz, *C*H_3_CH_2_OP), 16.45 (d, ^3^*J*_(CCOP)_ = 5.5 Hz, *C*H_3_CH_2_OP). ^31^P-NMR (243 MHz, CDCl_3_): δ = 22.13. Anal. Calcd. for C_23_H_26_F_2_N_3_O_5_P × 0.5 H_2_O: C, 54.98; H, 5.42; N, 8.36. Found: C, 55.08; H, 5.14; N, 8.26 (obtained on a 92:8 mixture of *trans*-**11i** and *cis*-**11i**).

*Diethyl* *trans-(2-methyl-5-(3-methyl-4-oxo-3,4-dihydroquinazolin-2-yl)isoxazolidin-3-yl)phosphonate* (*trans*-**11j**). Colorless oil; IR (film, cm^−1^) ν_max_: 2983, 2955, 2924, 2854, 1679, 1601, 1474, 1435, 1340, 1299, 1241, 1053, 1022, 945, 781, 695, 653, 600, 559. ^1^H-NMR (600 MHz, CDCl_3_): δ = 8.28–8.25 (m, 1H), 7.75–7.72 (m, 1H), 7.68–7.66 (m, 1H), 7.50–7.47 (m, 1H), 5.18 (dd, ^3^*J*_(H5–H4β)_ = 7.7 Hz, ^3^*J*_(H5–H4α)_ = 5.6 Hz, 1H, HC5), 4.30–4.22 (m, 4H, 2 × CH_2_OP), 3.75 (dddd, ^3^*J*_(H4α–P)_ = 14.0 Hz, ^2^*J*_(H4α–H4β)_ = 12.7 Hz, ^3^*J*_(H4α–H3)_ = 9.1 Hz, ^3^*J*_(H4α–H5)_ = 5.6 Hz, 1H, H_β_C4), 3.74 (s, 3H, C*H*_3_N), 3.37 (ddd, ^3^*J*_(H3–H4α)_ = 9.1 Hz, ^3^*J*_(H3–H4β)_ = 7.7 Hz, ^2^*J*_(H3-P)_ = 2.7 Hz, 1H, HC3), 2.79 (dddd, ^3^*J*_(H4β–P)_ = 15.4 Hz, ^2^*J*_(H4β–H4α)_ = 12.7 Hz, ^3^*J*_(H4β–H3)_ = 7.7 Hz, ^3^*J*_(H4β–H5)_ = 7.7 Hz, 1H, H_β_C4), 1.40 (t, ^3^*J* = 7.1 Hz, 3H, C*H*_3_CH_2_OP), 1.39 (t, ^3^*J* = 7.1 Hz, 3H, C*H*_3_CH_2_OP). ^13^C-NMR (151 MHz, CDCl_3_): δ = 162.50 (s, C(O)), 152.24, 146.52, 134.08, 127.62, 127.41, 126.79, 120.98, 76.37 (d, ^3^*J*_(CCCP)_ = 7.8 Hz, C5), 64.38 (d, ^1^*J*_(CP)_ = 170.6 Hz, C3), 63.02 (d, ^2^*J*_(COP)_ = 6.6 Hz, CH_2_OP), 62.70 (d, ^2^*J*_(COP)_ = 6.9 Hz, CH_2_OP), 47.21 (d, ^3^*J*_(CNCP)_ = 6.3 Hz, CH_3_N), 34.41 (s, C4), 30.77 (s, CH_3_N) 16.56 (d, ^3^*J*_(CCOP)_ = 5.0 Hz, *C*H_3_CH_2_OP), 16.53 (d, ^3^*J*_(CCOP)_ = 4.9 Hz, *C*H_3_CH_2_OP). ^31^P-NMR (243 MHz, CDCl_3_): δ = 22.09. Anal. Calcd. for C_17_H_24_N_3_O_5_P × H_2_O: C, 51.13; H, 6.56; N, 10.52. Found: C, 51.23; H, 6.17; N, 10.49.

*Diethyl* *trans-(5-(3-ethyl-4-oxo-3,4-dihydroquinazolin-2-yl)-2-methylisoxazolidin-3-yl)phosphonate* (*trans*-**11k**). Data noted below correspond to a 94:6 mixture of *trans*-**11k** and *cis*-**11k**. Yellowish oil; IR (film, cm^−1^) ν_max_: 3068, 2981, 2930, 2870, 1620, 1574, 1501, 1424, 1382, 1340, 1240, 1164, 1105, 1053, 1023, 967, 774, 684, 581. (signals of *trans*-**11k** were extracted from the spectra of a 94:6 mixture of *trans*-**11k** and *cis*-**11k**); ^1^H-NMR (600 MHz, CDCl_3_): δ = 8.19–8.17 (m, 1H), 7.96–7.94 (m, 1H), 7.84–7.82 (m, 1H), 7.58–7.55 (m, 1H), 5.26 (dd, ^3^*J*_(H5–H4β)_ = 7.4 Hz, ^3^*J*_(H5–H4α)_ = 6.4 Hz, 1H, HC5), 4.69 (q, ^3^*J* = 7.1 Hz, 2H, CH_3_C*H*_2_), 4.34–4.21 (m, 4H, 2 × CH_2_OP), 3.49–3.46 (m, 1H, HC3), 3.08 (s, 3H, C*H*_3_N), 3.10–2.96 (m, 2H, *H*_α_C4, H_β_C4), 1.55 (t, ^3^*J* = 7.1 Hz, 3H, C*H*_3_CH_2_), 1.42 (t, ^3^*J* = 7.0 Hz, 3H, C*H*_3_CH_2_OP), 1.39 (t, ^3^*J* = 7.1 Hz, 3H, C*H*_3_CH_2_OP). ^13^C-NMR (151 MHz, CDCl_3_): δ =167.25 (s, C(O)), 163.16, 151.03, 133.51, 127.77, 126.92, 123.44, 115.60, 80.36 (d, ^3^*J*_(CCCP)_ = 8.3 Hz, C5), 64.38 (d, ^1^*J*_(CP)_ = 168.2 Hz, C3), 63.23 (d, ^2^*J*_(COP)_ = 6.4 Hz, CH_2_OP), 62.36 (d, ^2^*J*_(COP)_ = 6.7 Hz, CH_2_OP), 47.21 (d, ^3^*J*_(CNCP)_ = 6.3 Hz, CH_3_N), 34.41 (s, C4), 29.64 (s, CH_3_*C*H_2_) 16.53 (d, ^3^*J*_(CCOP)_ = 5.7 Hz, *C*H_3_CH_2_OP), 16.47 (d, ^3^*J*_(CCOP)_ = 5.8 Hz, *C*H_3_CH_2_OP), 14.29 (s, *C*H_3_CH_2_). ^31^P-NMR (243 MHz, CDCl_3_): δ = 22.23. Anal. Calcd. for C_18_H_26_N_3_O_5_P: C, 54.68; H, 6.63; N, 10.63. Found: C, 54.93; H, 6.51; N, 10.21 (obtained on a 94:6 mixture of *trans*-**11k** and *cis*-**11k**).

### 3.5. Antiviral Activity Assays

The compounds were evaluated against different herpesviruses, including herpes simplex virus type 1 (HSV-1) strain KOS, thymidine kinase-deficient (TK^−^) HSV-1 KOS strain resistant to ACV (ACV^r^), herpes simplex virus type 2 (HSV-2) strain G, varicella-zoster virus (VZV) strain Oka, TK^−^ VZV strain 07-1, human cytomegalovirus (HCMV) strains AD-169 and Davis as well as feline herpes virus (FHV), the poxvirus vaccinia virus (Lederle strain), para-influenza-3 virus, reovirus-1, Sindbis virus, Coxsackie virus B4, Punta Toro virus, respiratory syncytial virus (RSV), feline coronovirus (FIPV) and influenza A virus subtypes H1N1 (A/PR/8), H3N2 (A/HK/7/87) and influenza B virus (B/HK/5/72) and human immune deficiency virus (5HVV-1 and HIV-2). The antiviral assays, other than HIV, were based on inhibition of virus-induced cytopathicity or plaque formation in human embryonic lung (HEL) fibroblasts, African green monkey kidney cells (Vero), human epithelial cervix carcinoma cells (HeLa), Crandell-Rees feline kidney cells (CRFK), or Madin Darby canine kidney cells (MDCK). Confluent cell cultures in microtiter 96-well plates were inoculated with 100 CCID_50_ of virus (1 CCID_50_ being the virus dose to infect 50% of the cell cultures) or with 20 plaque forming units (PFU) and the cell cultures were incubated in the presence of varying concentrations of the test compounds. Viral cytopathicity or plaque formation (VZV) was recorded as soon as it reached completion in the control virus-infected cell cultures that were not treated with the test compounds. Antiviral activity was expressed as the EC_50_ or compound concentration required to reduce virus-induced cytopathicity or viral plaque formation by 50%. Cytotoxicity of the test compounds was expressed as the minimum cytotoxic concentration (MCC) or the compound concentration that caused a microscopically detectable alteration of cell morphology.

### 3.6. Cytostatic Activity against Immortalized Cell Lines

Murine leukemia (L1210), human T-lymphocyte (CEM), human cervix carcinoma (HeLa) and immortalized human dermal microvascular endothelial cells (HMEC-1) were suspended at 300,000–500,000 cells/mL of culture medium, and 100 μL of a cell suspension was added to 100 μL of an appropriate dilution of the test compounds in 200 μL-wells of 96-well microtiter plates. After incubation at 37 °C for two (L1210), three (CEM) or four (HeLa) days, the cell number was determined using a Coulter counter. The IC_50_ was defined as the compound concentration required to inhibit cell proliferation by 50%.

## 4. Conclusions

A series of isoxazolidine-containing quinazolinones *trans*-**11** and *cis*-**11** have been synthesised from *N*-methyl-*C*-diethoxyphosphorylnitrone (**12**) and the respective N3-substituted 2-vinyl-quinazolin-ones **13** via the 1,3-dipolar cycloaddition. The obtained isoxazolidine phosphonates *trans*-**11** or the respective mixtures of *trans*-**11**/*cis*-**11** were evaluated against a variety of DNA and RNA viruses. Among all tested compounds, isoxazolidines *trans*-**11f**/*cis*-**11f** (90:10), *trans*-**11h** and *trans*-**11i**/*cis*-**11i** were slightly active toward TK^+^VZV strain (EC_50_ = 6.84, 15.29 and 9.44 μM) without exhibiting cytotoxicity toward uninfected cells at concentration up to 100 μM. On the other hand, phosphonates *trans*-**11b**/*cis*-**11b** (90:10), *trans*-**11c**, *trans*-**11e**/*cis*-**11e** (90:10) and *trans*-**11g** showed weak antiviral properties against cytomegalovirus (EC_50_ = 27–45 μM).

Compounds equipped with benzyl substituents at N3 in the quinazolinone skeleton exhibited some antiproliferative activity toward the tested immortalized cells (IC_50_ = 21–102 μM), while analogues lacking substituent or having alkyl group at N3 were inactive. These results encourage us to continue the search for more active compounds with special focus on modification the quinazoline-4-one unit especially at N3.

## Figures and Tables

**Table 1:** Isoxazolidines *trans*-**11** and *cis*-**11** obtained according to Scheme 3.

Entry	Quinazolinone 13	Ratio of *Trans*-11:*Cis*-11	Yield (%)
	R		
**a**	H	92:8	*trans*-**11a** (23) ^a^ + *trans*-**11a** and *cis*-**11a** (70) ^b^
**b**	C_6_H_5_-CH_2_	90:10	*trans*-**11b** and *cis*-**11b** (84) ^b^
**c**	2-NO_2_-C_6_H_4_-CH_2_	90:10	*trans*-**11c** (20) ^a^ + *trans*-**11c** and *cis*-**11c** (73) ^b^
**d**	3-NO_2_-C_6_H_4_-CH_2_	92:8	*trans*-**11d** and *cis*-**11d** (98) ^b^
**e**	4-NO_2_-C_6_H_4_-CH_2_	90:10	*trans*-**11e** and *cis*-**11e** (91) ^b^
**f**	2-F-C_6_H_4_-CH_2_	90:10	*trans*-**11f** and *cis*-**11f** (94) ^b^
**g**	3-F-C_6_H_4_-CH_2_	90:10	*trans*-**11g** (7) ^a^ + *trans*-**11g** and *cis*-**11g** (89) ^b^
**h**	4-F-C_6_H_4_-CH_2_	90:10	*trans*-**11h** (22) ^a^ + *trans*-**11h** and *cis*-**11h** (70) ^b^
**i**	2,4-diF-C_6_H_3_-CH_2_	92:8	*trans*-**11i** and *cis*-**11i** (94) ^b^
**j**	Me	94:6	*trans*-**11j** (19) ^a^ + *trans*-**11j** and *cis*-**11j** (78) ^b^
**k**	Et	92:8	*trans*-**11k** and *cis*-**11k** (86) ^b^

^a^ yield of pure isomer; ^b^ yield of pure mixture of *cis*- and *trans*-isomers.

**Table 2:** Cytotoxicity and antiviral activity against varicella-zoster virus (VZV) in HEL cell cultures.

Compound	R	Antiviral Activity EC_50_ (μM) ^a^		Cytotoxicity (μM)
		TK^+^ VZV Strain	TK^−^ VZV Strain	Cell Morphology MCC ^b^
*trans*-**11c**	2-NO_2_-C_6_H_4_-CH_2_	46.47	100	>100
*trans*-**11e**/*cis*-**11e** (90:10)	4-NO_2_-C_6_H_4_-CH_2_	34.20	42.87	100
*trans*-**11f**/*cis*-**11f** (90:10)	2-F-C_6_H_4_-CH_2_	6.84	>20	100
*trans*-**11h**	4-F-C_6_H_4_-CH_2_	15.29	>20	100
*trans*-**11i**/*cis*-**11i** (97:3)	2,4-diF-C_6_H_3_-CH_2_	9.44	>20	100
*trans*-**11k**	CH_3_CH_2_	38.80	41.57	>100
Acyclovir		0.71	39.69	>100
Brivudin		0.019	25.59	>100

^a^ Effective concentration required to reduce virus plaque formation by 50%. Virus input was 100 plaque forming units (PFU); ^b^ Minimum cytotoxic concentration that causes a microscopically detectable alternation of cell morphology.

**Table 3:** Antiviral activity and cytotoxicity against human cytomegalovirus in HEL cell cultures.

Compound	R	Antiviral Activity EC_50_ (μM) ^a^		Cytotoxicity (μM)
		AD-169 Strain	Davis Strain	Cell Morphology MCC ^b^
*trans*-**11b**/*cis*-**11b** (90:10)	C_6_H_5_-CH_2_	44.72	>20	≥100
*trans*-**11c**	2-NO_2_-C_6_H_4_-CH_2_	>100	44.72	≥20
*trans*-**11e**/*cis*-**11e** (90:10)	4-NO_2_-C_6_H_4_-CH_2_	44.72	20	>100
*trans*-**11g**	3-F-C_6_H_4_-CH_2_	>100	27.59	100
Ganciclovir		10.52	0.63	>350
Cidofovir		1.49	0.23	>300

^a^ Effective concentration required to reduce virus plaque formation by 50%. Virus input was 100 plaque forming units (PFU); ^b^ Minimum cytotoxic concentration that causes a microscopically detectable alternation of cell morphology.

**Table 4:** Inhibitory effect of the tested compounds against the proliferation of murine leukemia (L1210), human T-lymphocyte (CEM), human cervix carcinoma (HeLa) and immortalized human dermal microvascular endothelial cells (HMEC-1).

Compound	R	IC_50_^a^ (μM)			
		L1210	CEM	HeLa	HMEC-1
*trans*-**11a**	H	>250	>250	>250	>250
*trans*-**11b**/*cis*-**11b** (90:10)	C_6_H_5_-CH_2_	49 ± 23	28 ± 4	82 ± 5	83 ± 16
*trans*-**11c**	2-NO_2_-C_6_H_4_-CH_2_	87 ± 22	76 ± 9	97 ± 0	92 ± 1
*trans*-**11d**/*cis*-**11d** (90:10)	3-NO_2_-C_6_H_4_-CH_2_	28 ± 11	21 ± 4	50 ± 4	58 ± 0
*trans*-**11e**/*cis*-**11e** (90:10)	4-NO_2_-C_6_H_4_-CH_2_	59 ± 32	34 ± 12	76 ± 4	102 ± 3
*trans*-**11f**/*cis*-**11f** (90:10)	2-F-C_6_H_4_-CH_2_	33 ± 7	29 ± 13	76 ± 11	77 ± 0
*trans*-**11g**	3-F-C_6_H_4_-CH_2_	35 ± 12	26 ± 6	62 ± 9	74 ± 3
*trans*-**11h**	4-F-C_6_H_4_-CH_2_	26 ± 1	30 ± 12	58 ± 5	78 ± 1
*trans*-**11i**/*cis*-**11i** (97:3)	2,4-diF-C_6_H_3_-CH_2_	26 ± 2	24 ± 8	55 ± 10	67 ± 4
*trans*-**11j**	Me	>250	>250	>250	>250
*trans*-**11k**/*cis*-**11k** (97:3)	Et	101 ± 17	85 ± 13	97 ± 10	86 ± 11
5-Fluorouracil		0.33 ± 0.17	18 ± 5	0.54 ± 0.12	n.d.

^a^ 50% Inhibitory concentration or compound concentration required to inhibit tumor cell proliferation by 50%; n.d.—not determined.

## Supplementary Materials

Supplementary materials can be accessed at: http://www.mdpi.com/1420-3049/21/7/959/s1.
