# Supplementary material for: New Isoxazolidine-Conjugates of Quinazolinones—Synthesis, Antiviral and Cytostatic Activity
Source: Molecules. 2016 Jul 22;21(7):959. doi: 10.3390/molecules21070959 (PMC6273226; doi:10.3390/molecules21070959)
Supplement: Supplementary file 1 [file molecules-21-00959-s001.pdf]

Molecules **2016**, *21*, 959; doi:10.3390/molecules21070959

Supplementary Materials: New Isoxazolidine-Conjugates of Quinazolinones—Synthesis, Antiviral and Cytostatic activity

Dorota G. Piotrowska, Graciela Andrei, Dominique Schols, Robert Snoeck and Magdalena Grabkowska-Drużyc

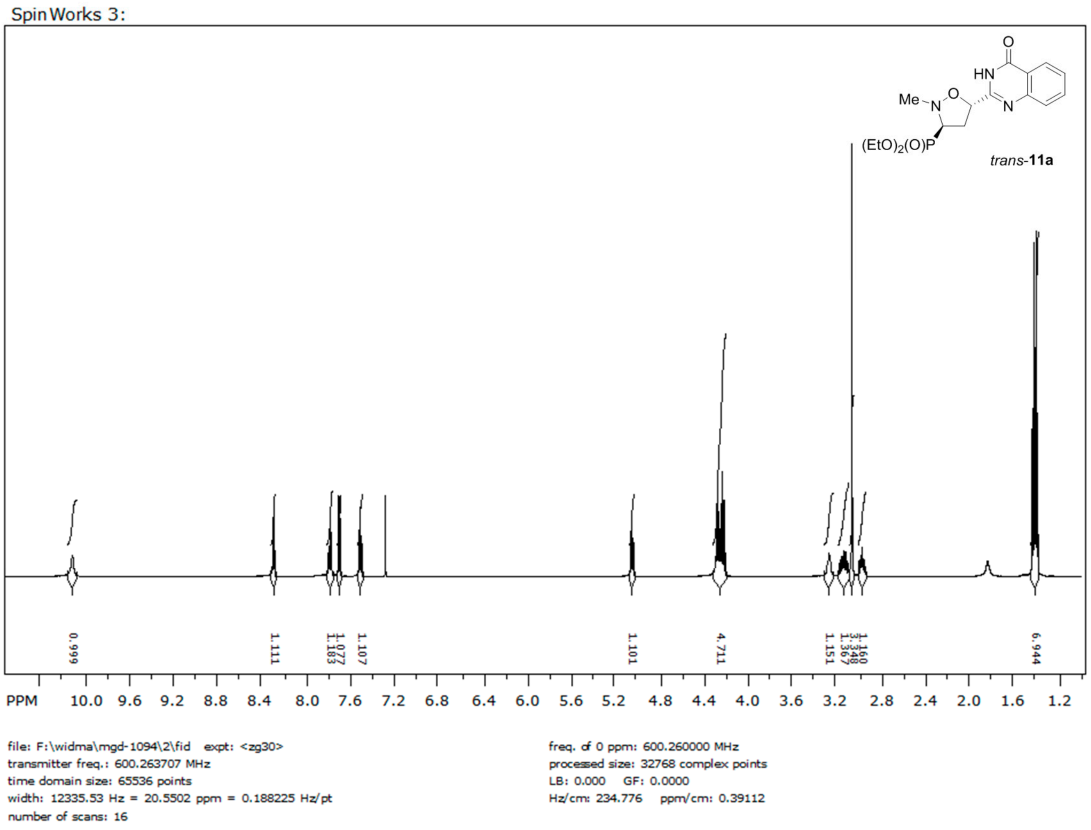

Figure S1. The ^{1}H-NMR spectrum of isoxazolidine *trans*-**11a**.

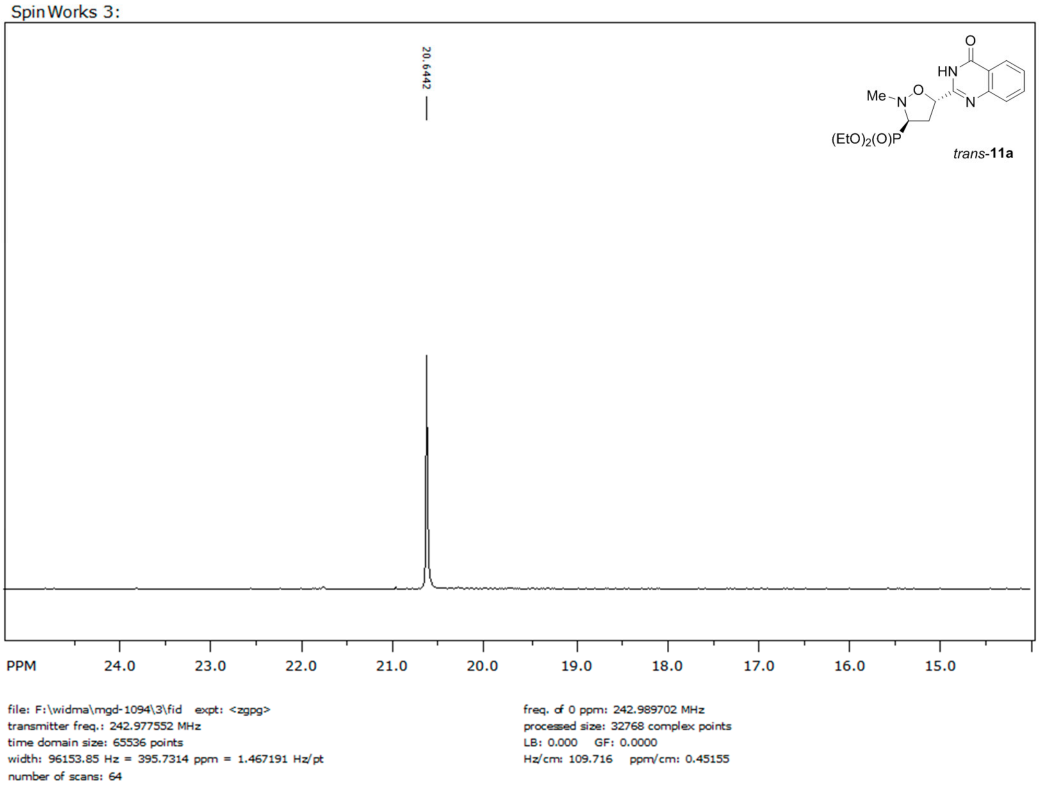

Figure S2. The ^{31}P-NMR spectrum of isoxazolidine *trans*-**11a**.

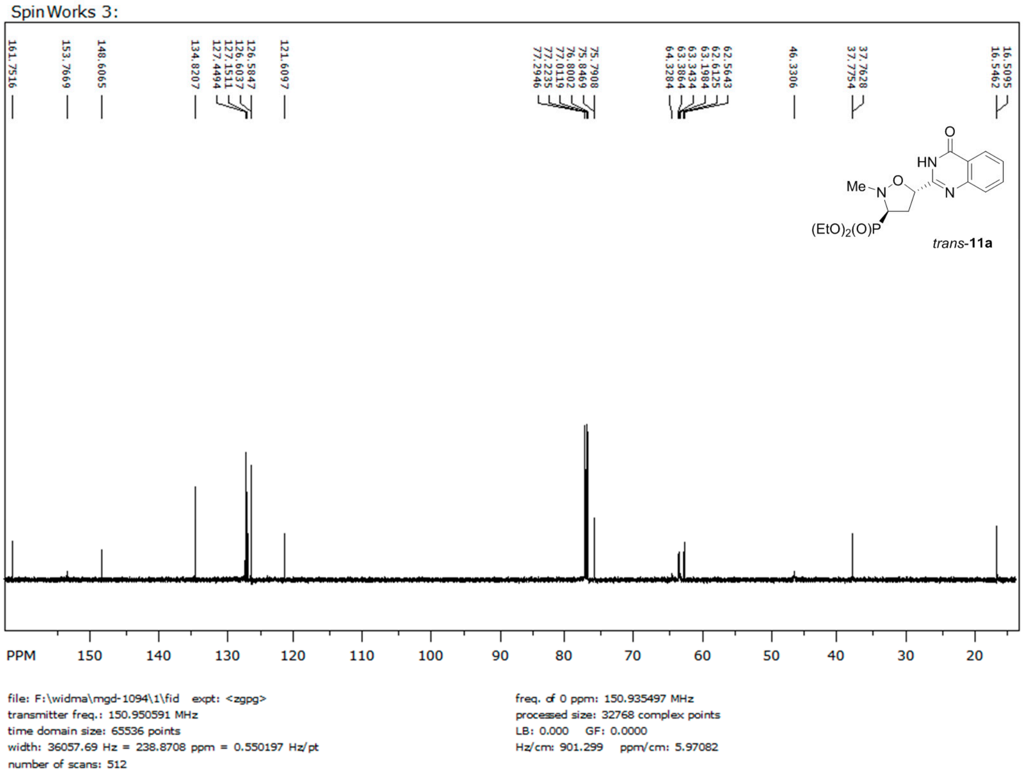

Figure S3. The ^{13}C-NMR spectrum of isoxazolidine *trans*-**11a**.

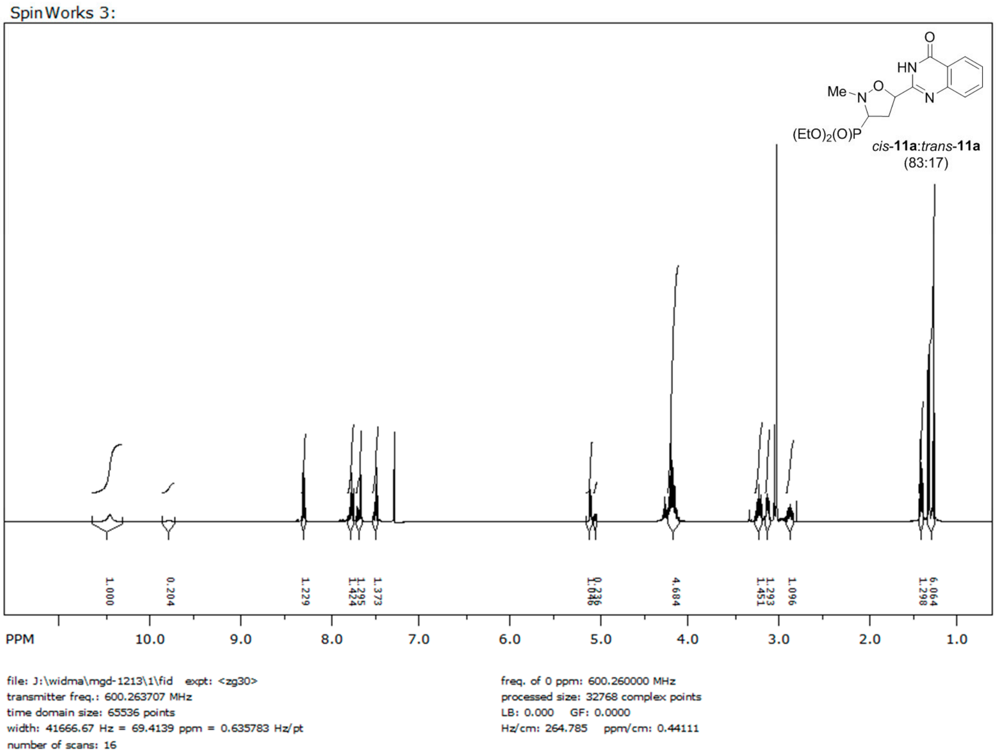

Figure S4. The ^{1}H-NMR spectrum of a 83:17 mixture of isoxazolidines *cis*-**11a** and *trans*-**11a**.

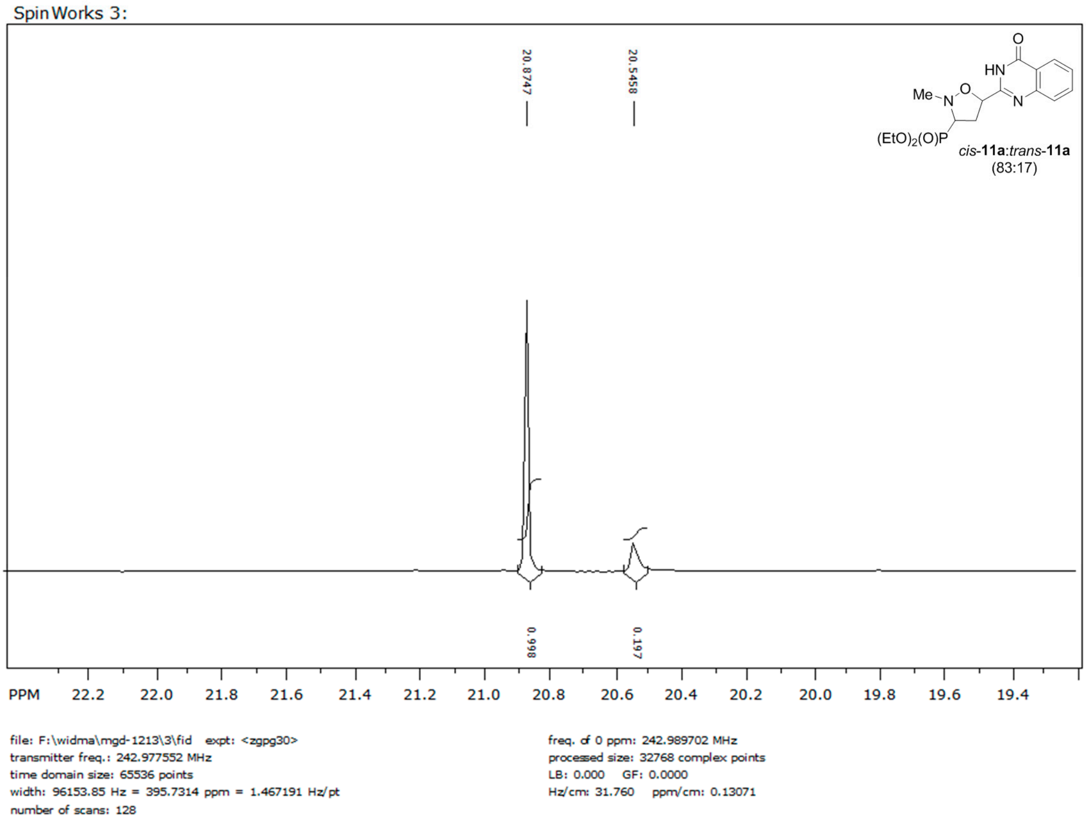

Figure S5. The ^{31}P-NMR spectrum of a 83:17 mixture of isoxazolidines *cis*-**11a** and *trans*-**11a**.

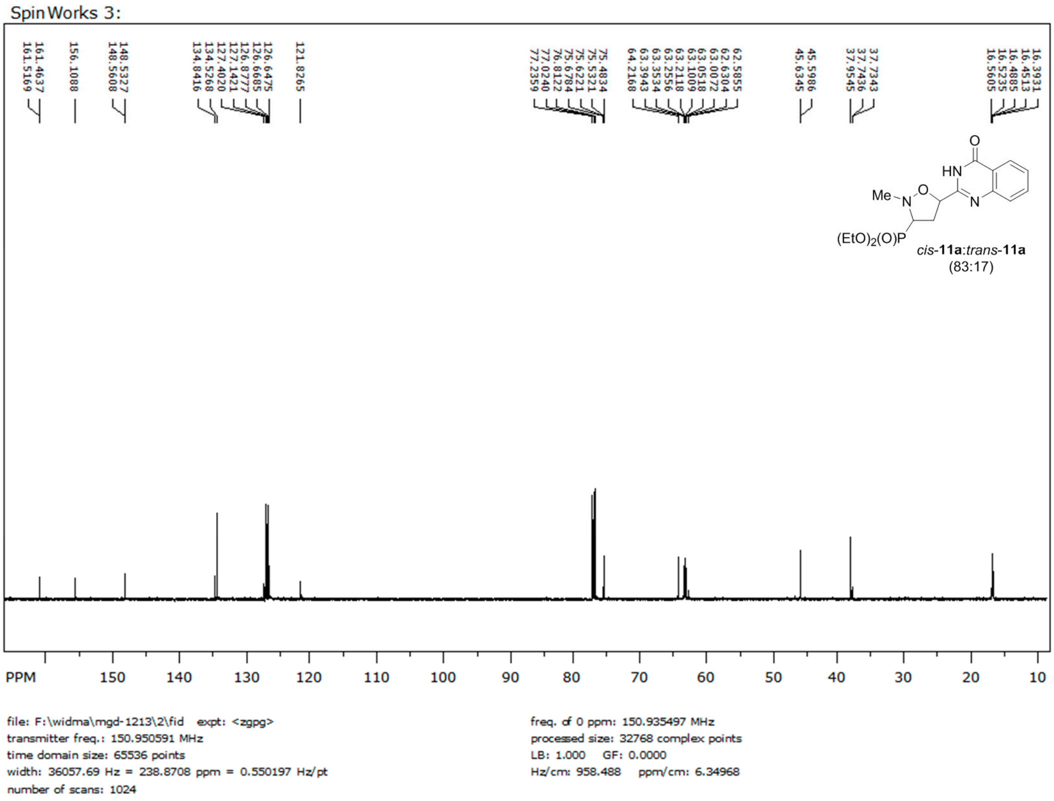

Figure S6. The ^{13}C-NMR spectrum of a 83:17 mixture of isoxazolidines *cis*-**11a** and *trans*-**11a**.

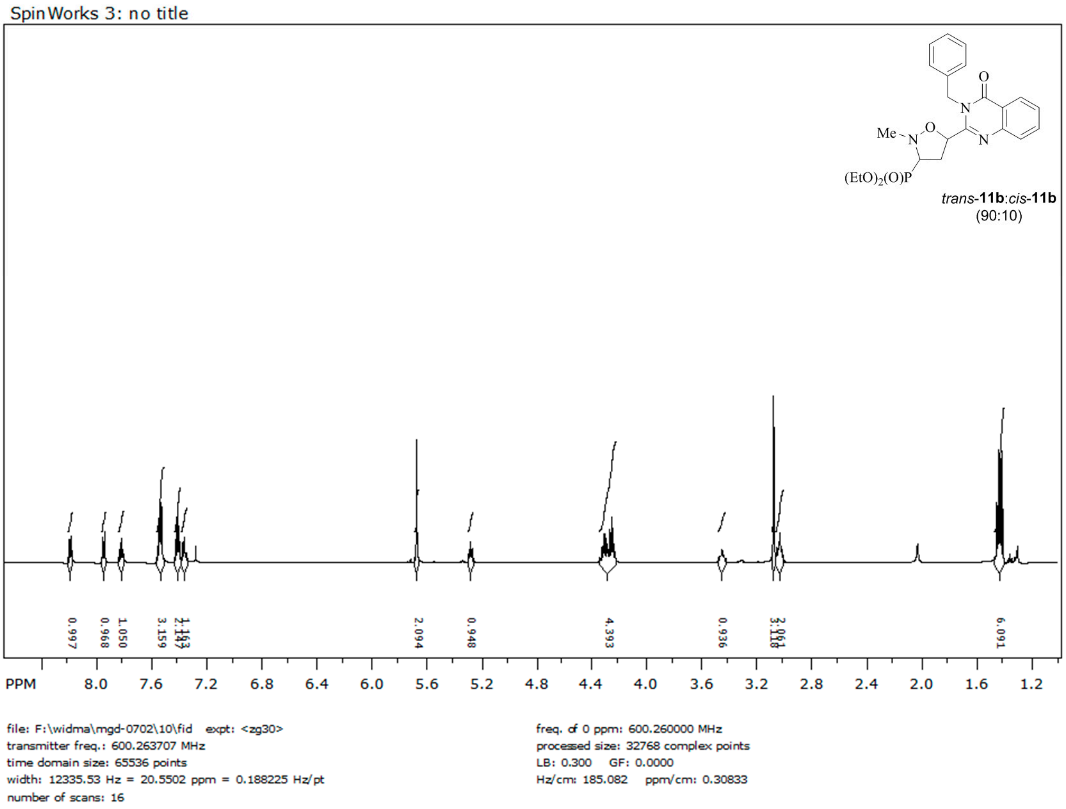

Figure S7. The ^{1}H-NMR spectrum of a 90:10 mixture of isoxazolidines *trans*-**11b** and *cis*-**11b**.

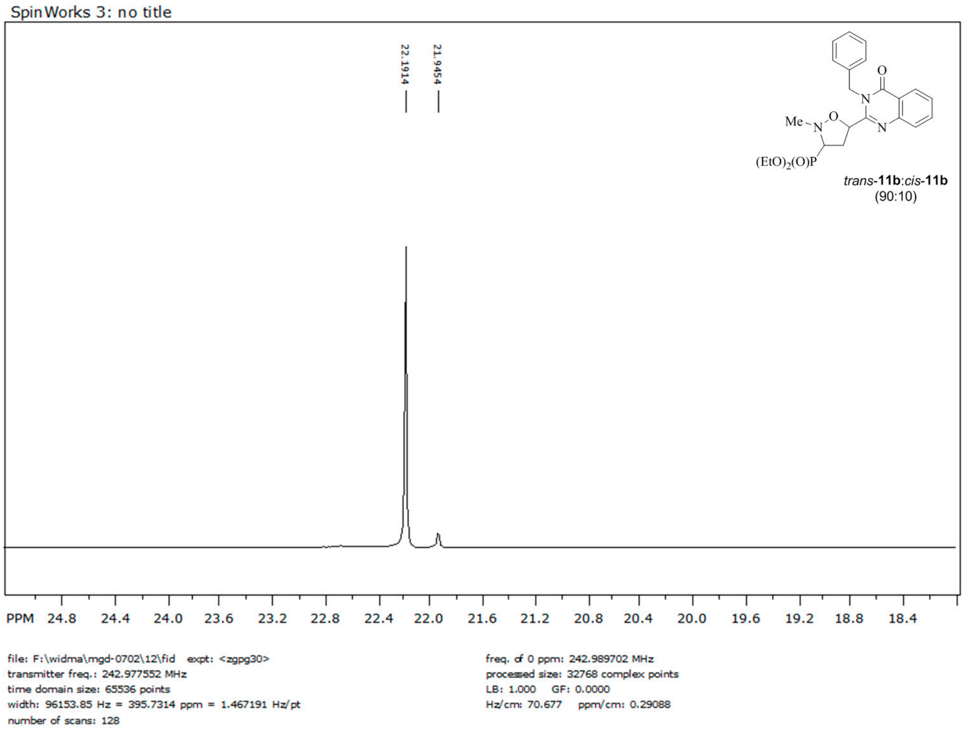

Figure S8. The ^{31}P-NMR spectrum of a 90:10 mixture of isoxazolidines *trans*-**11b** and *cis*-**11b**.

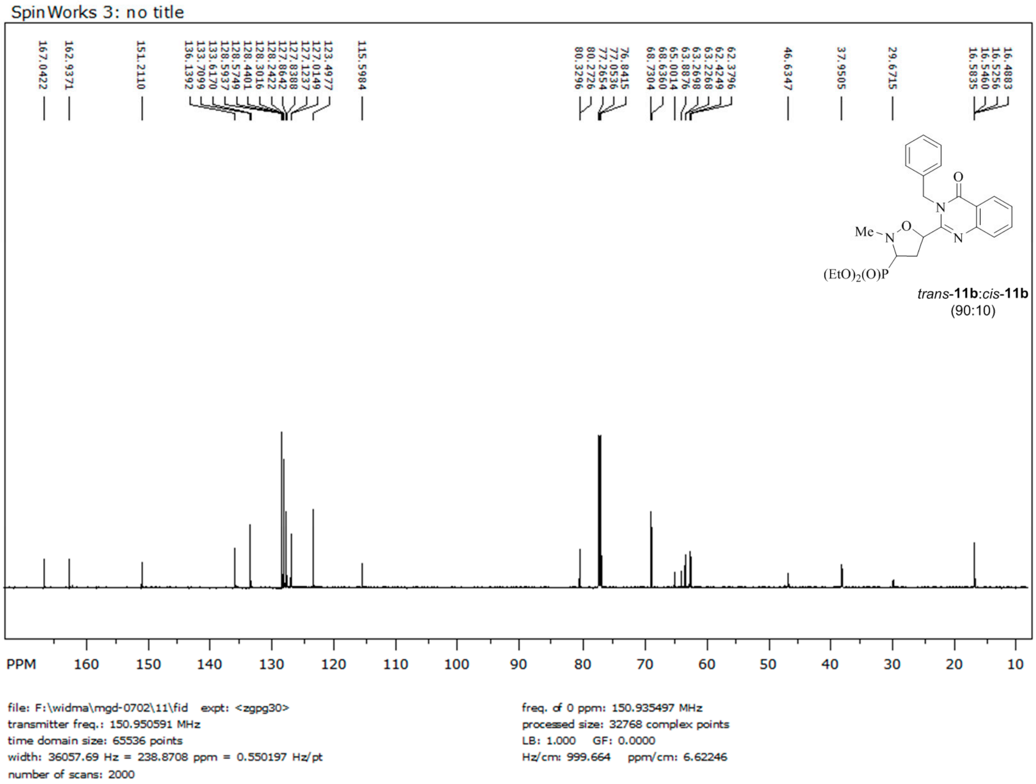

Figure S9. The ^{13}C-NMR spectrum of a 90:10 mixture of isoxazolidines *trans*-**11b** and *cis*-**11b**.

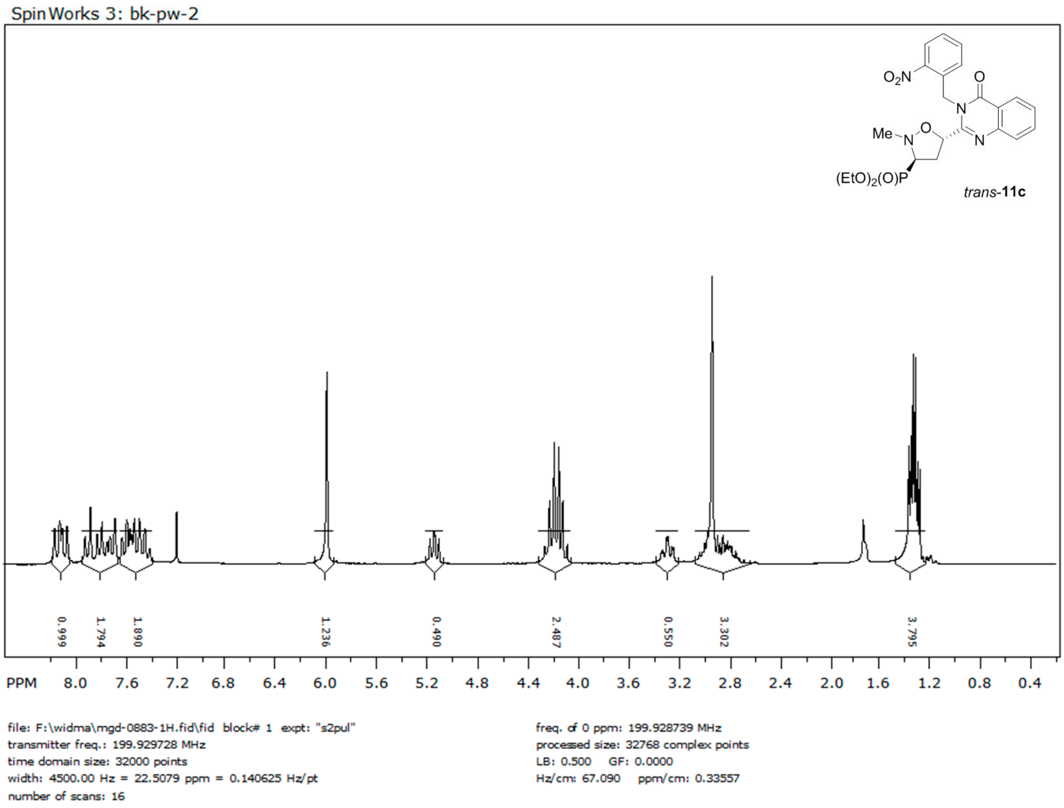

Figure S10. The ^{1}H-NMR spectrum of isoxazolidine *trans*-**11c**.

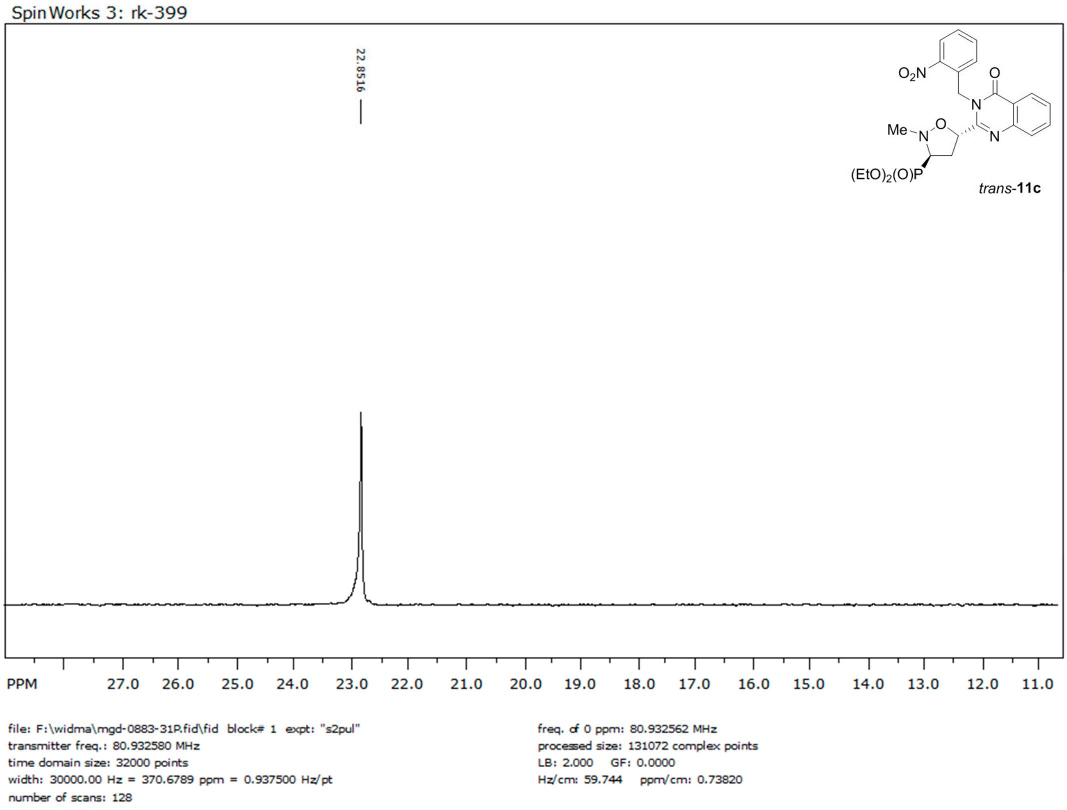

Figure S11. The ^{31}P-NMR spectrum of isoxazolidine *trans*-**11c**.

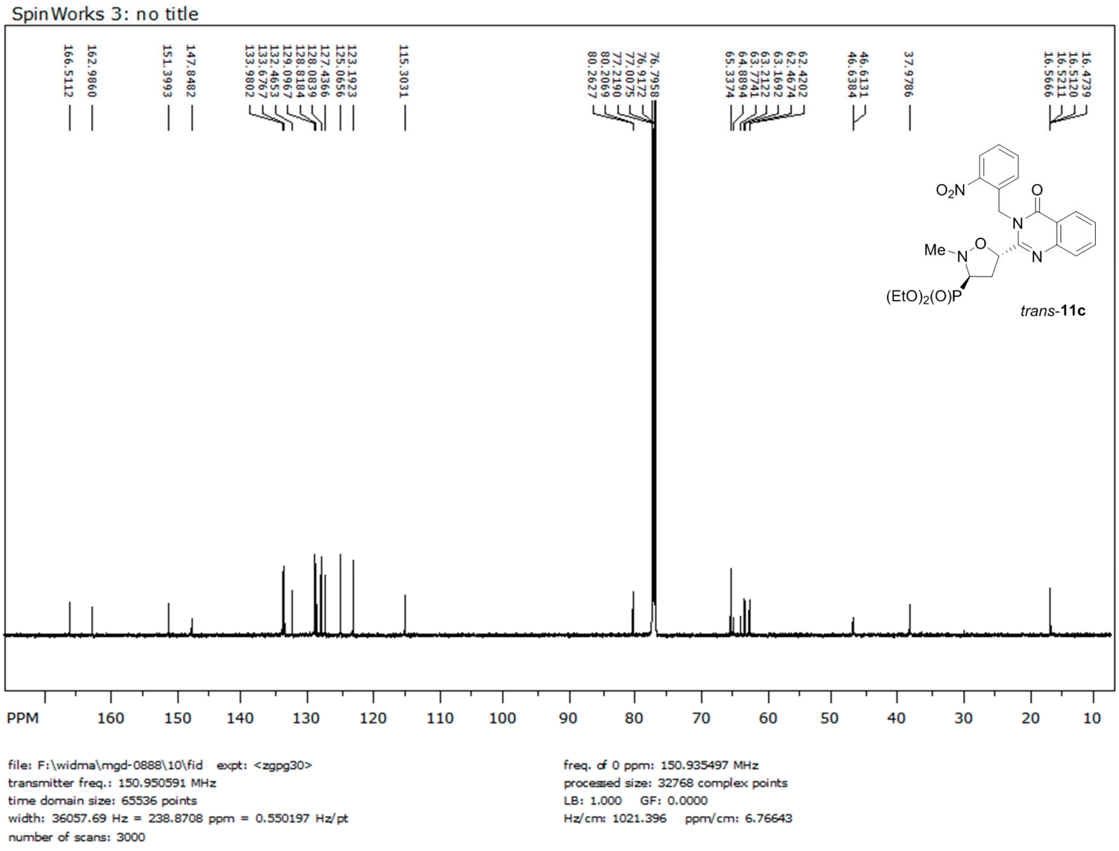

Figure S12. The ^{13}C-NMR spectrum of isoxazolidine *trans*-**11c**.

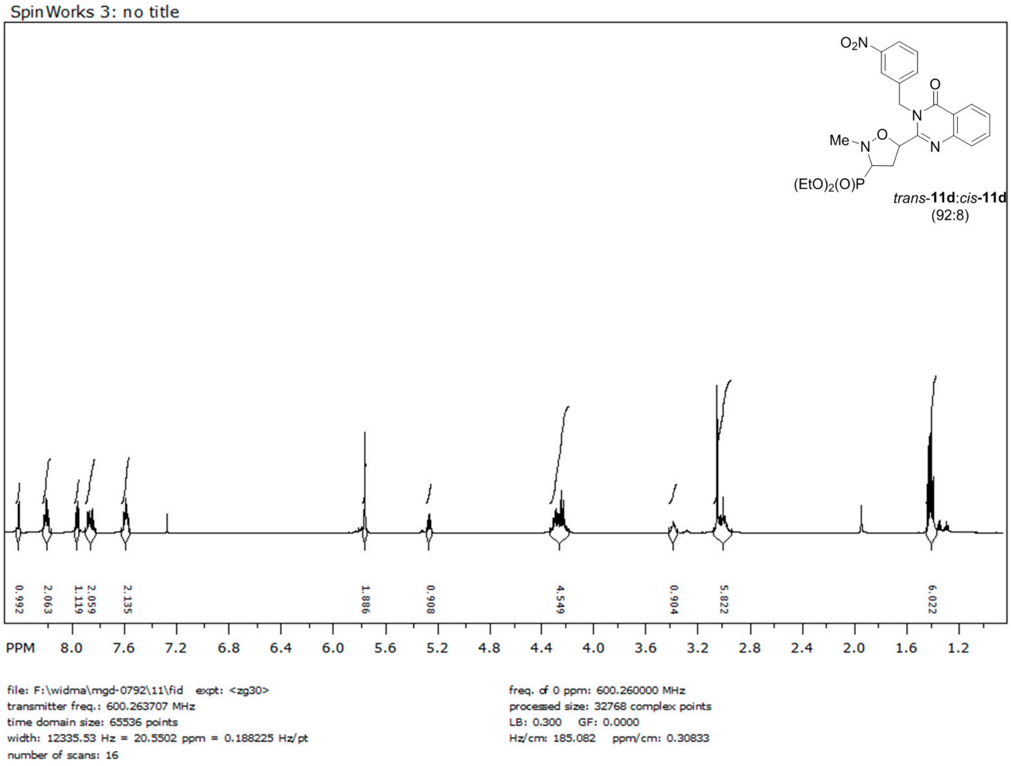

Figure S13. The ^{1}H-NMR spectrum of a 92:8 mixture of isoxazolidines *trans*-**11d** and *cis*-**11d**.

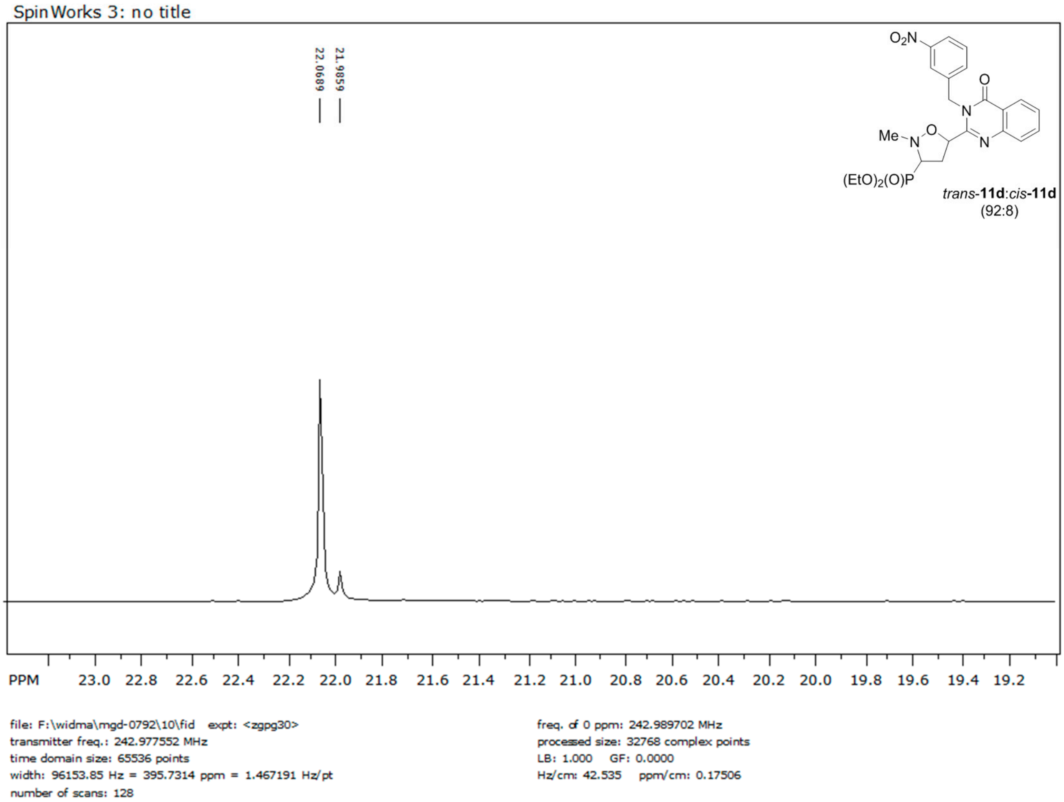

Figure S14. The ^{31}P-NMR spectrum of a 92:8 mixture of isoxazolidines *trans*-**11d** and *cis*-**11d**.

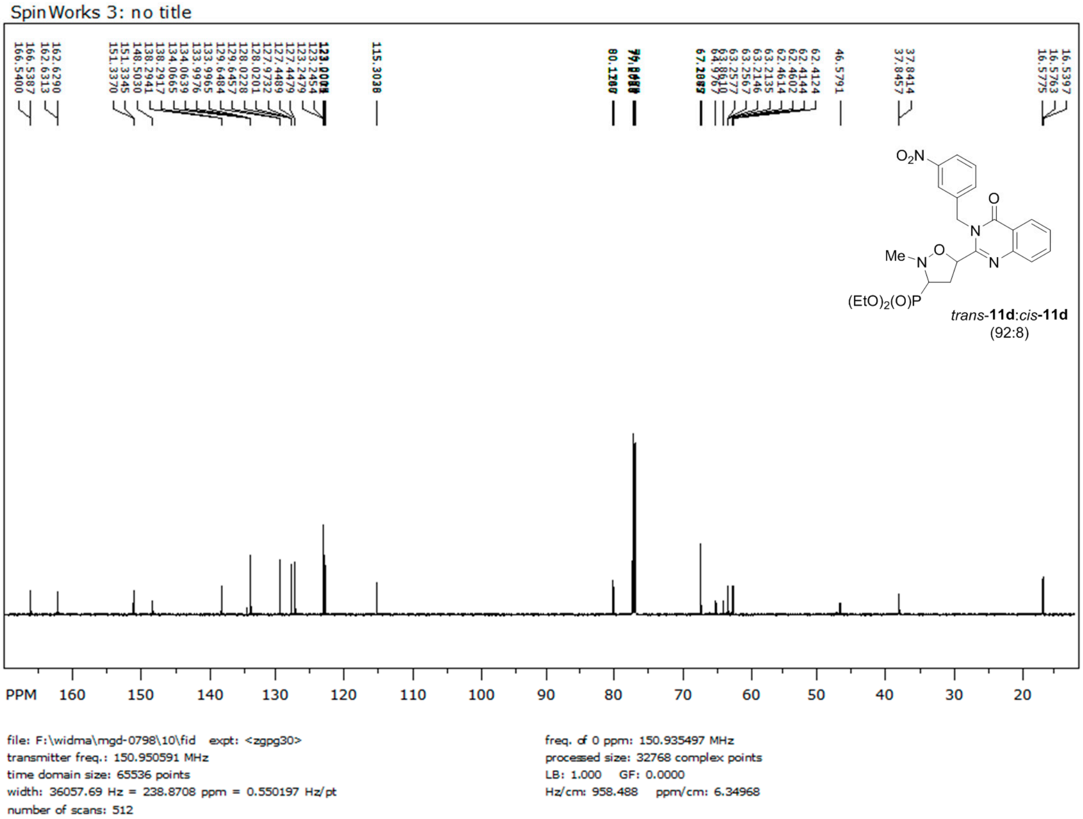

Figure S15. The ^{13}C-NMR spectrum of a 92:8 mixture of isoxazolidines *trans*-**11d** and *cis*-**11d**.

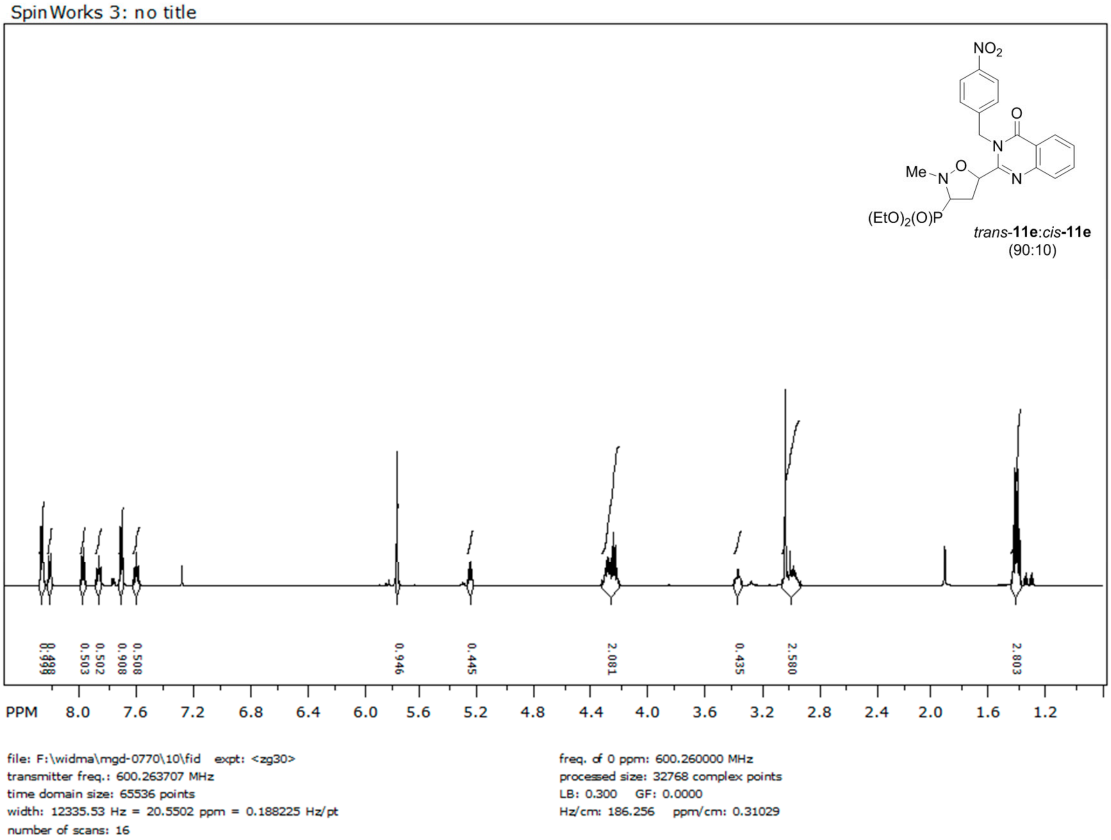

Figure S16. The ^{1}H-NMR spectrum of a 90:10 mixture of isoxazolidines *trans*-**11e** and *cis*-**11e**.

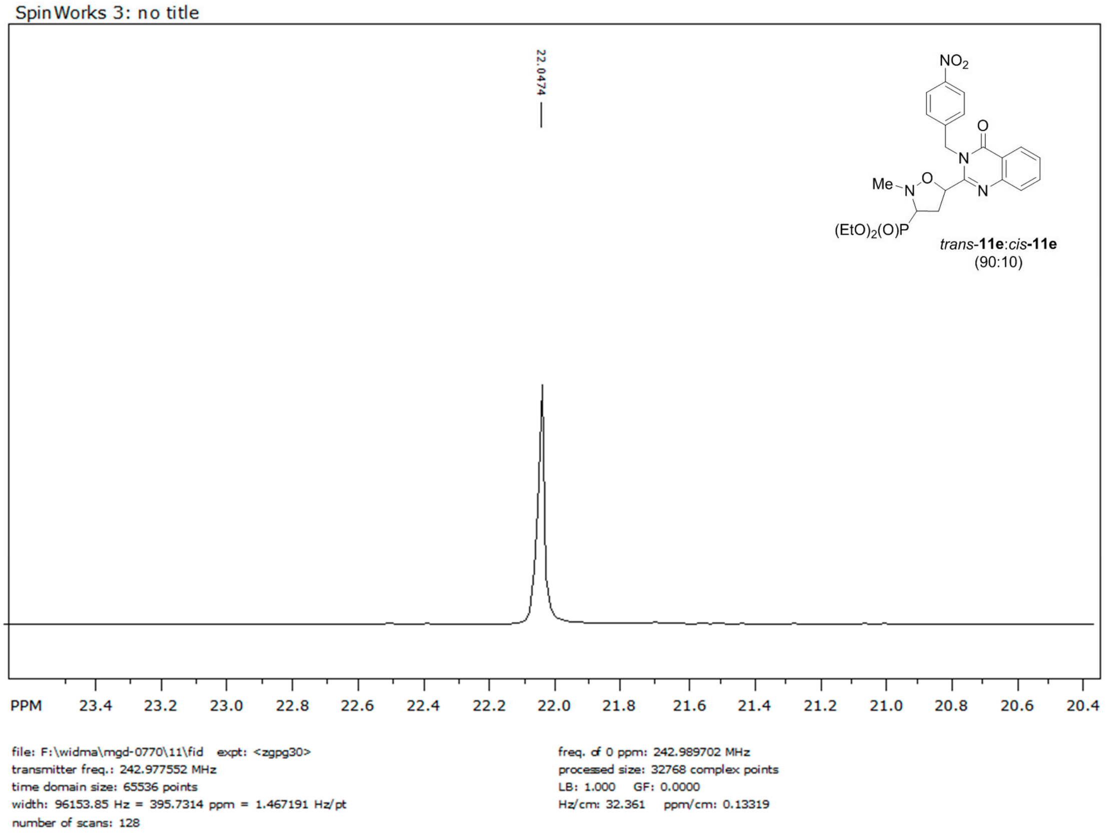

Figure S17. The ^{31}P-NMR spectrum of a 90:10 mixture of isoxazolidines *trans*-**11e** and *cis*-**11e**.

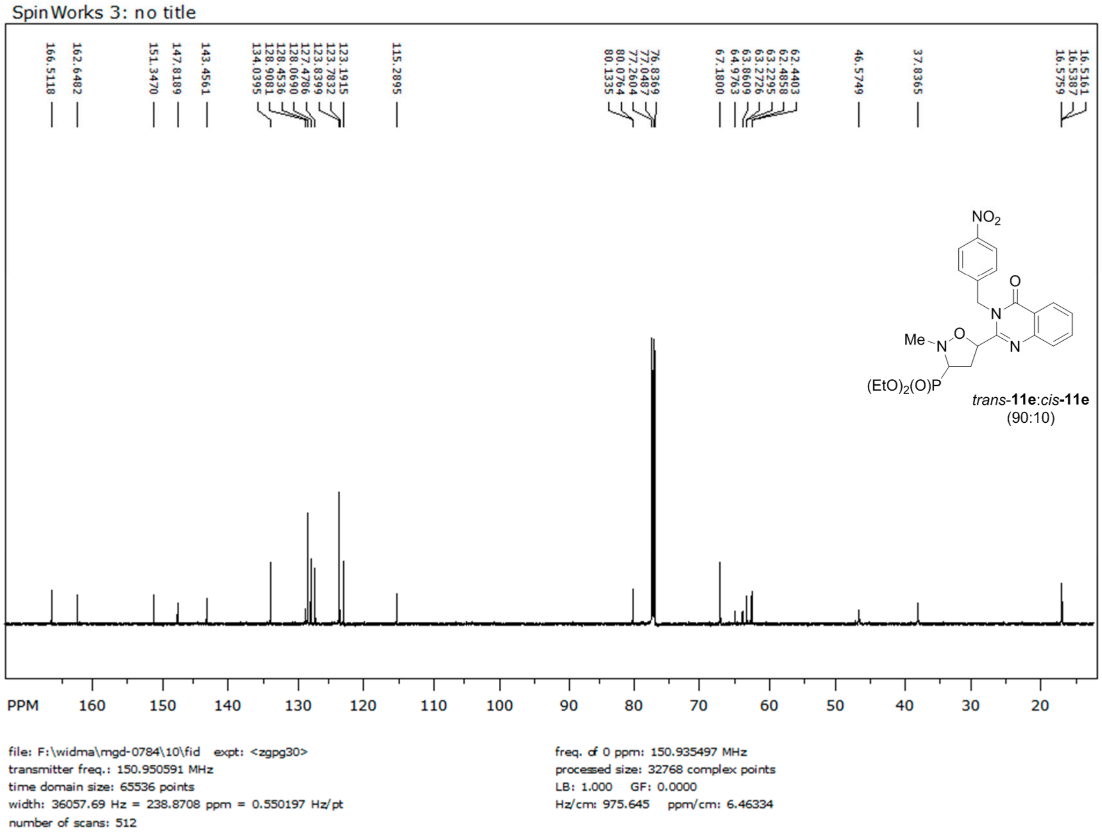

Figure S18. The ^{13}C-NMR spectrum of a 90:10 mixture of isoxazolidines *trans*-**11e** and *cis*-**11e**.

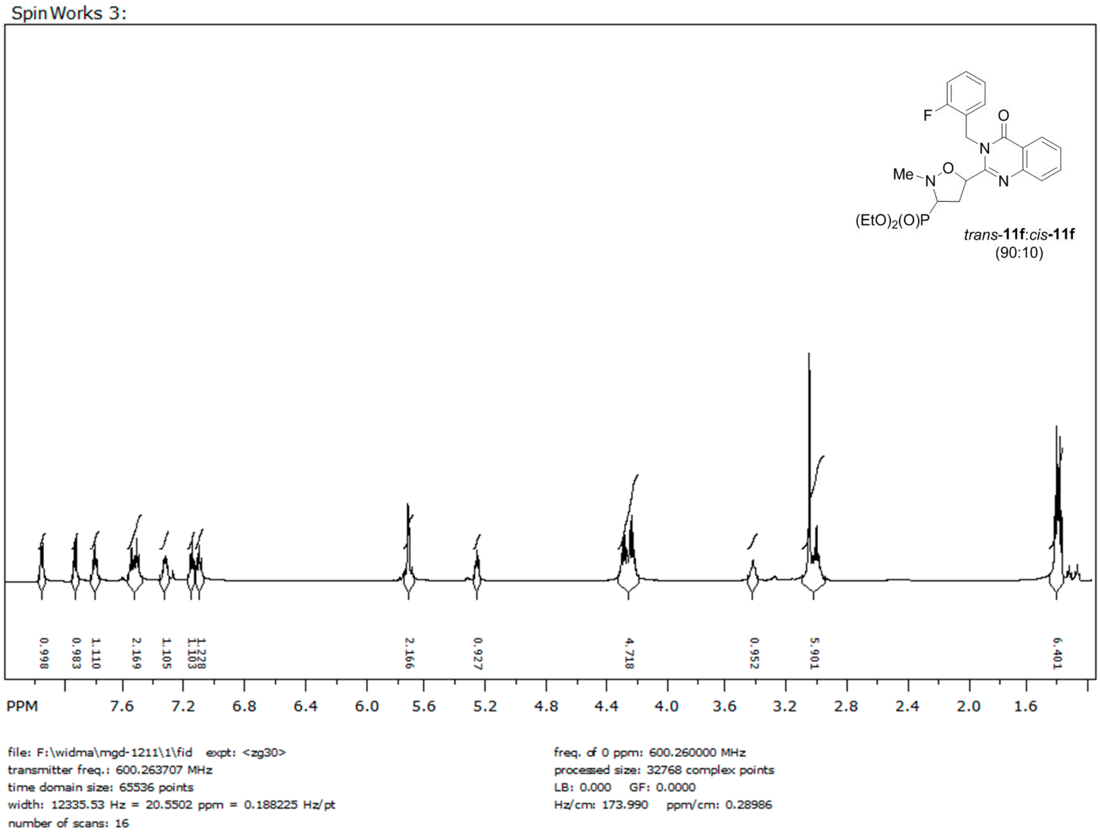

Figure S19. The ^{1}H-NMR spectrum of a 90:10 mixture of isoxazolidines *trans*-**11f** and *cis*-**11f**.

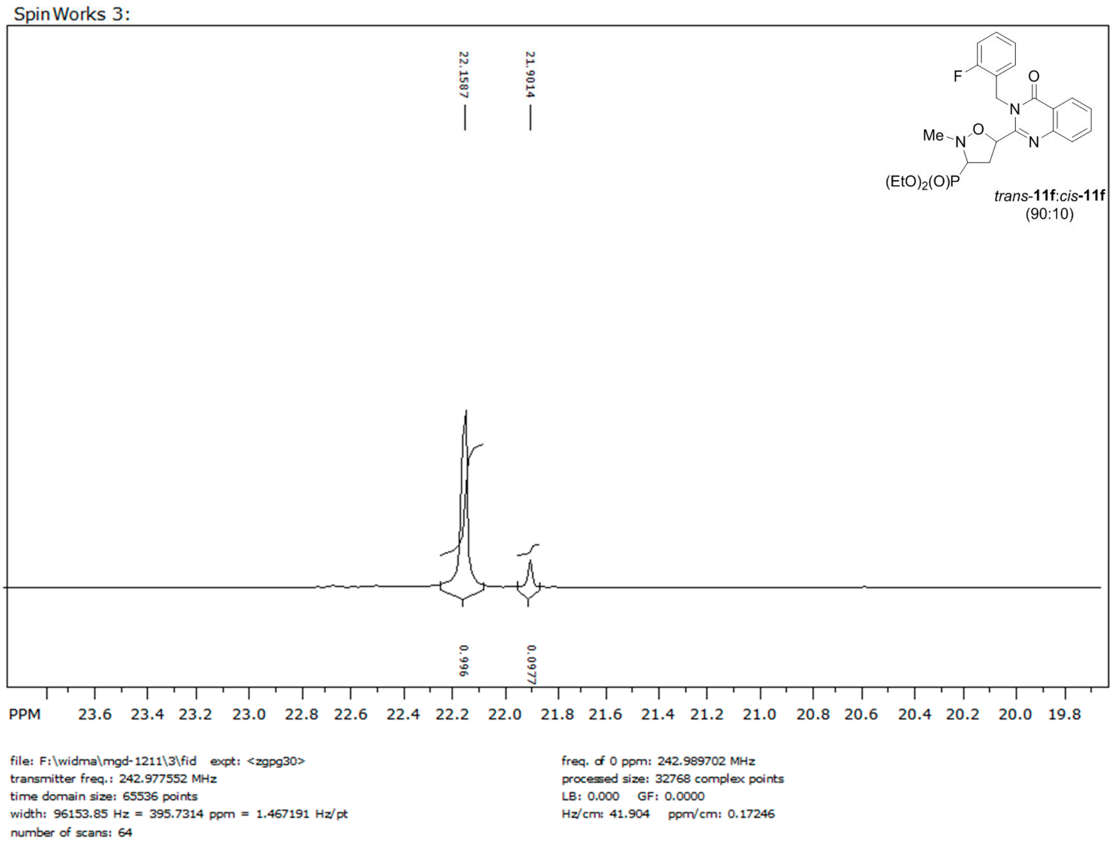

Figure S20. The ^{31}P-NMR spectrum of a 90:10 mixture of isoxazolidines *trans*-**11f** and *cis*-**11f**.

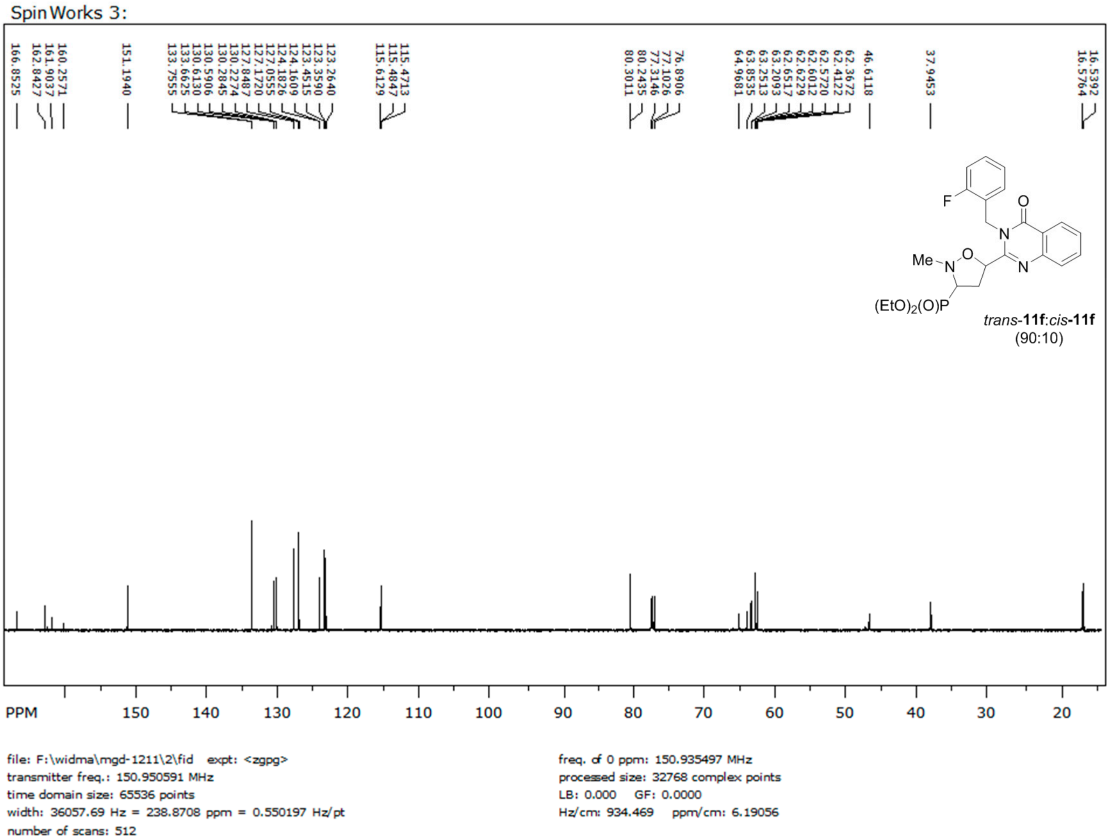

Figure S21. The ^{13}C-NMR spectrum of a 90:10 mixture of isoxazolidines *trans*-**11f** and *cis*-**11f**.

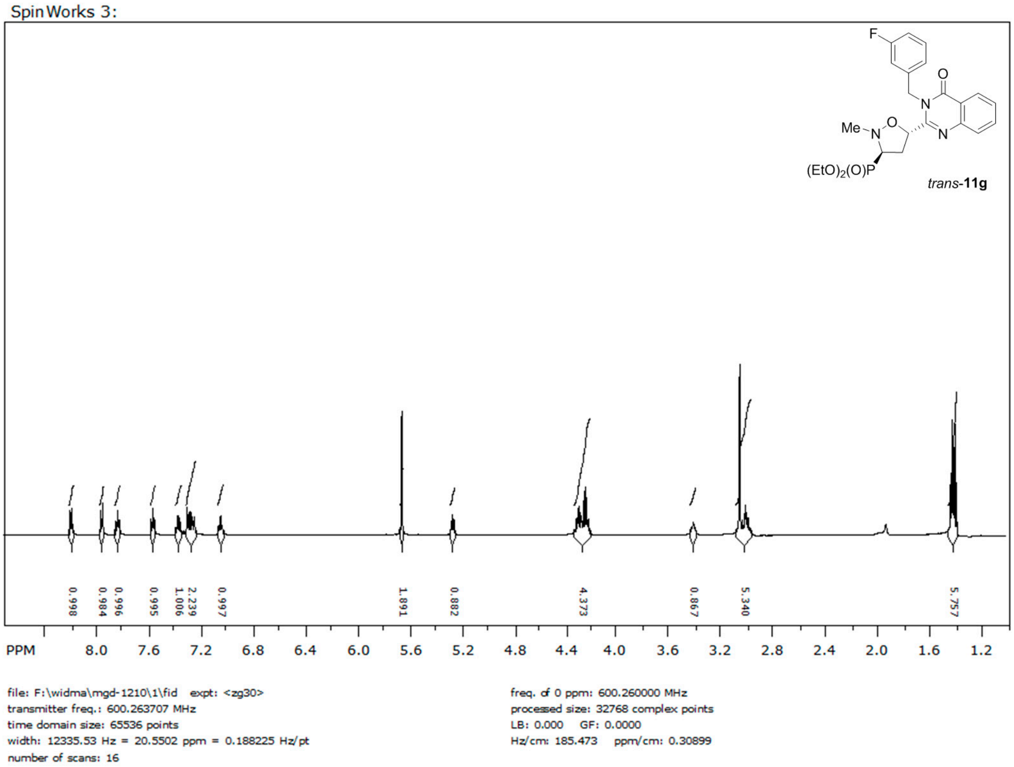

Figure S22. The ^{1}H-NMR spectrum of isoxazolidine *trans*-**11g**.

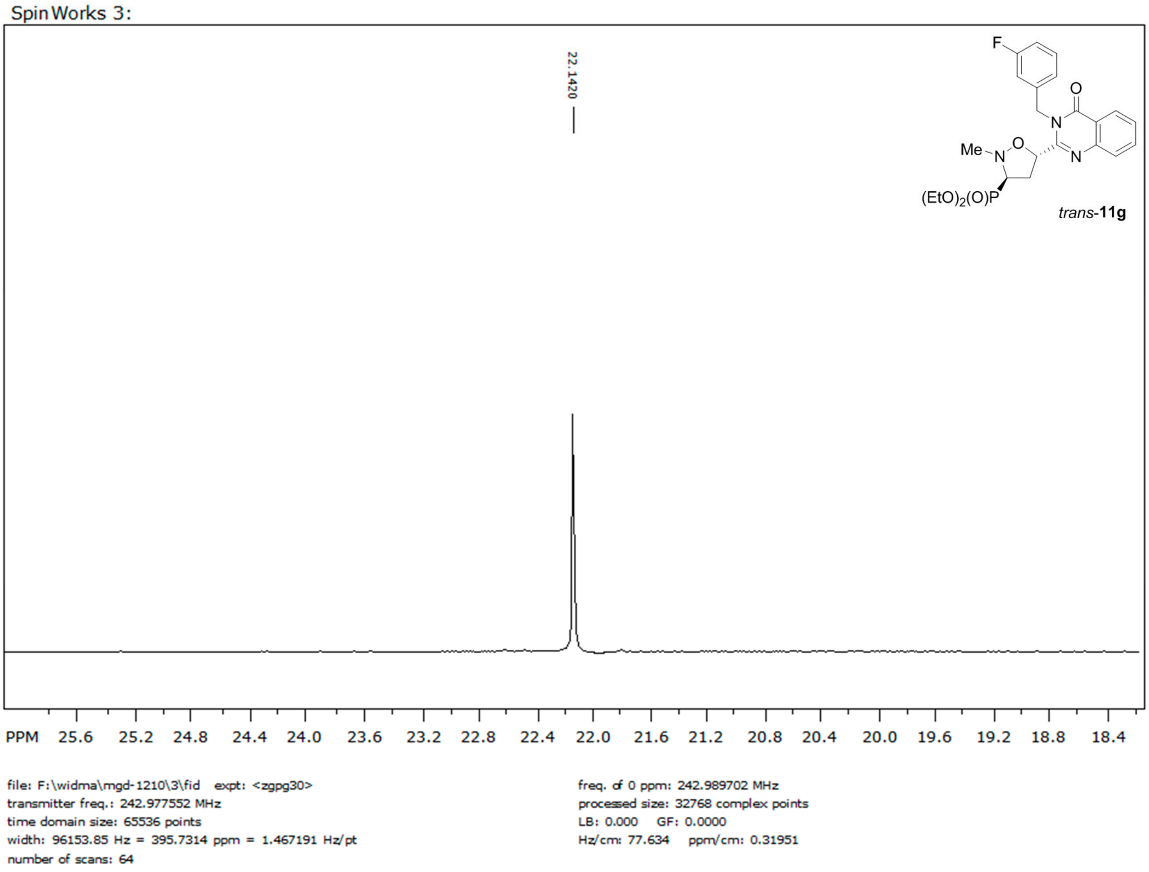

Figure S23. The ^{31}P-NMR spectrum of isoxazolidine *trans*-**11g**.

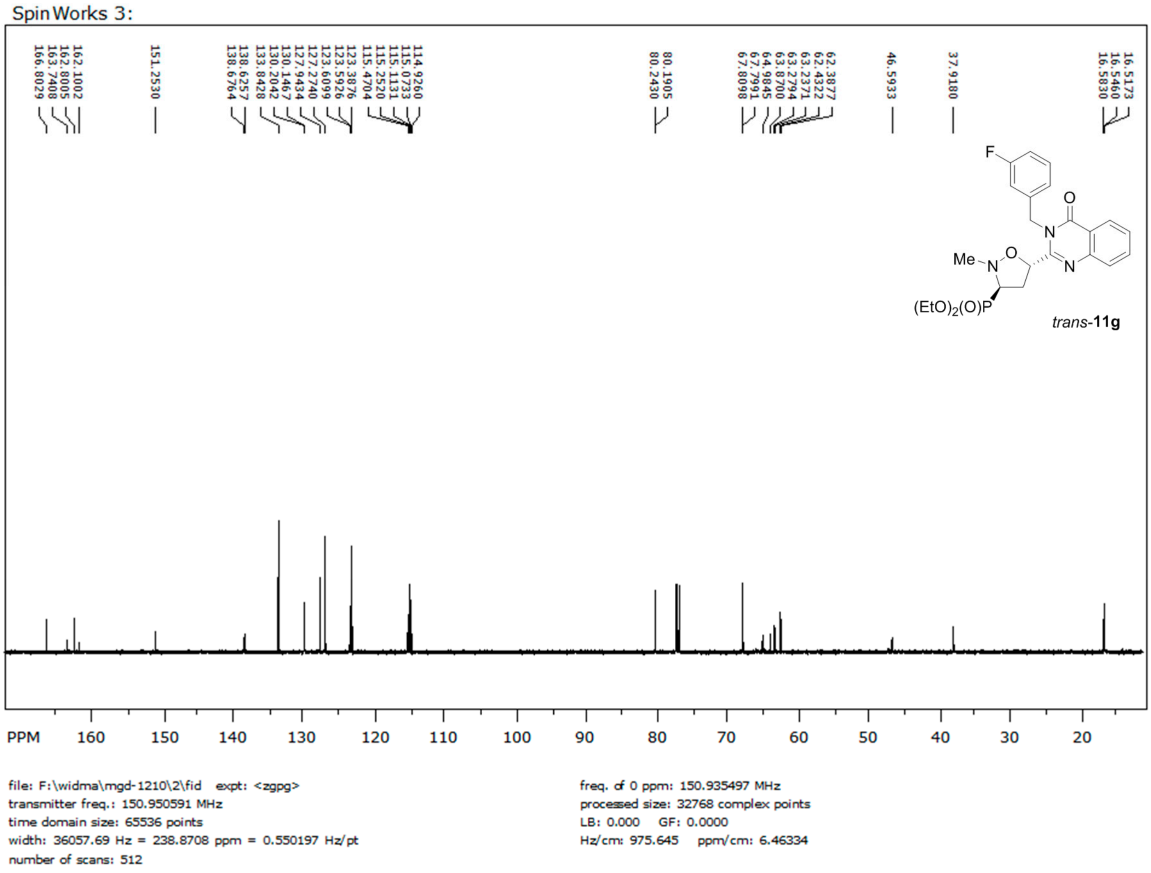

Figure S24. The ^{13}C-NMR spectrum of isoxazolidine *trans*-**11g**.

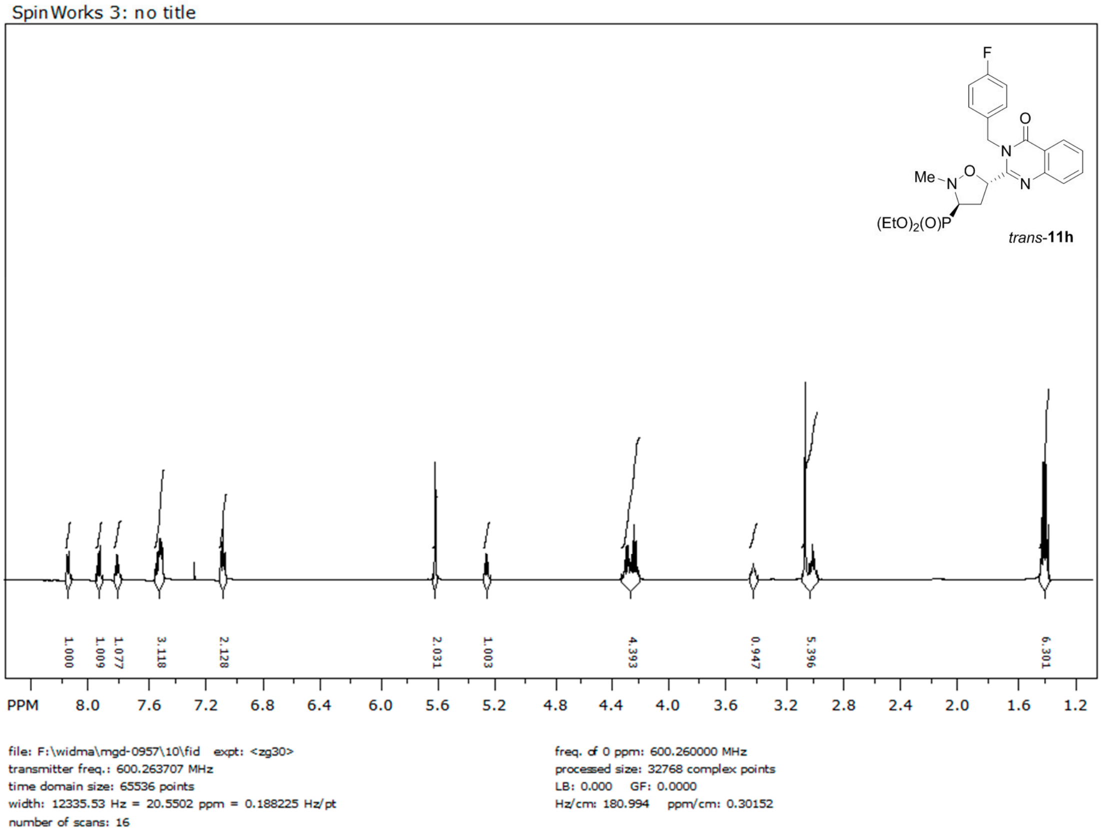

Figure S25. The ^{1}H-NMR spectrum of isoxazolidine *trans*-**11h**.

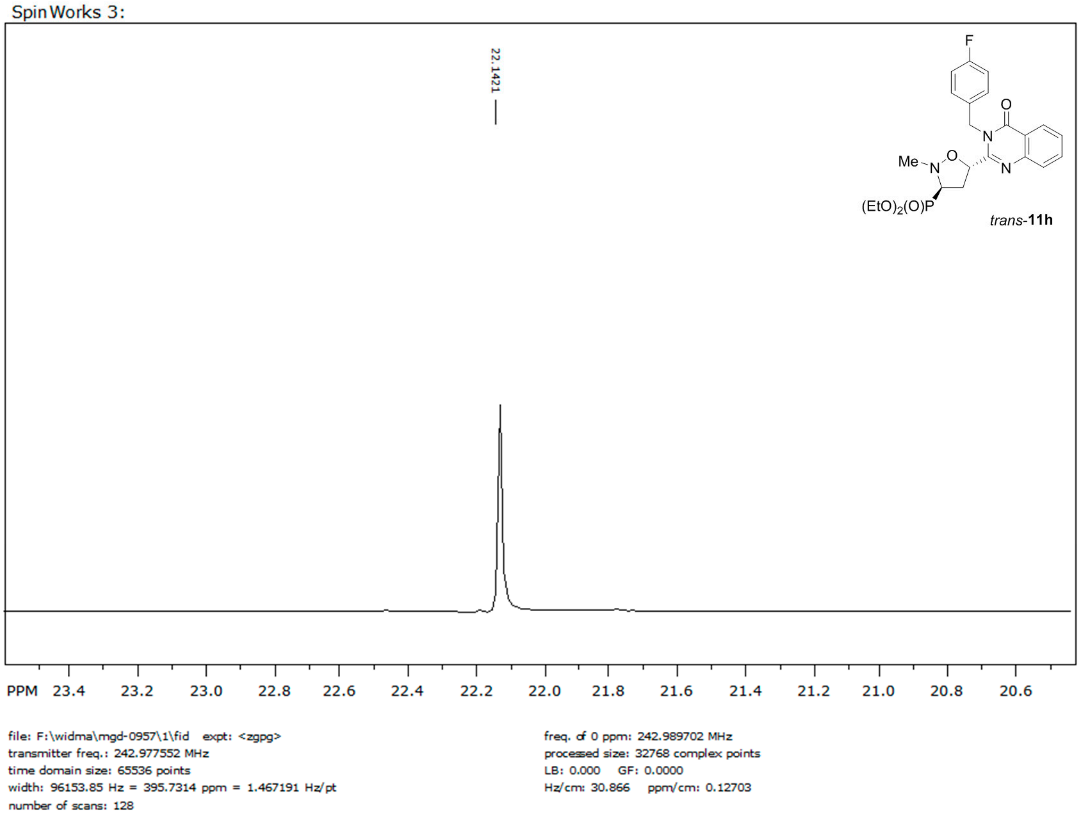

Figure S26. The ^{31}P-NMR spectrum of isoxazolidine *trans*-**11h**.

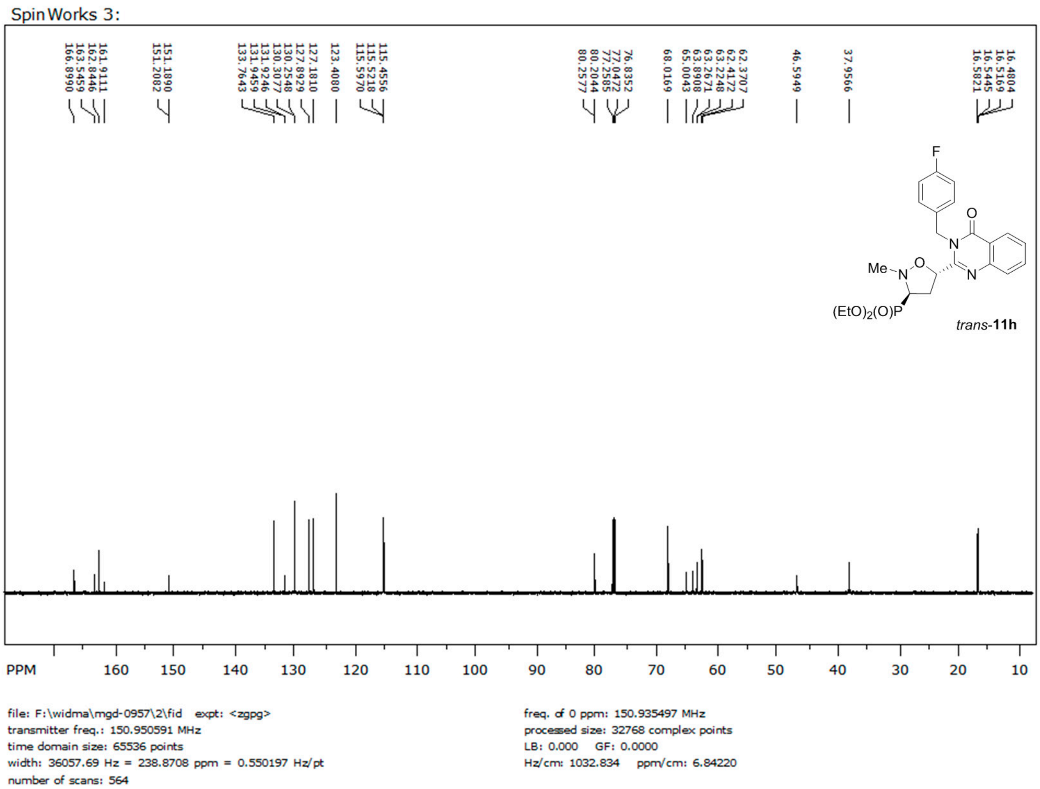

Figure S27. The ^{13}C-NMR spectrum of isoxazolidine *trans*-**11h**.

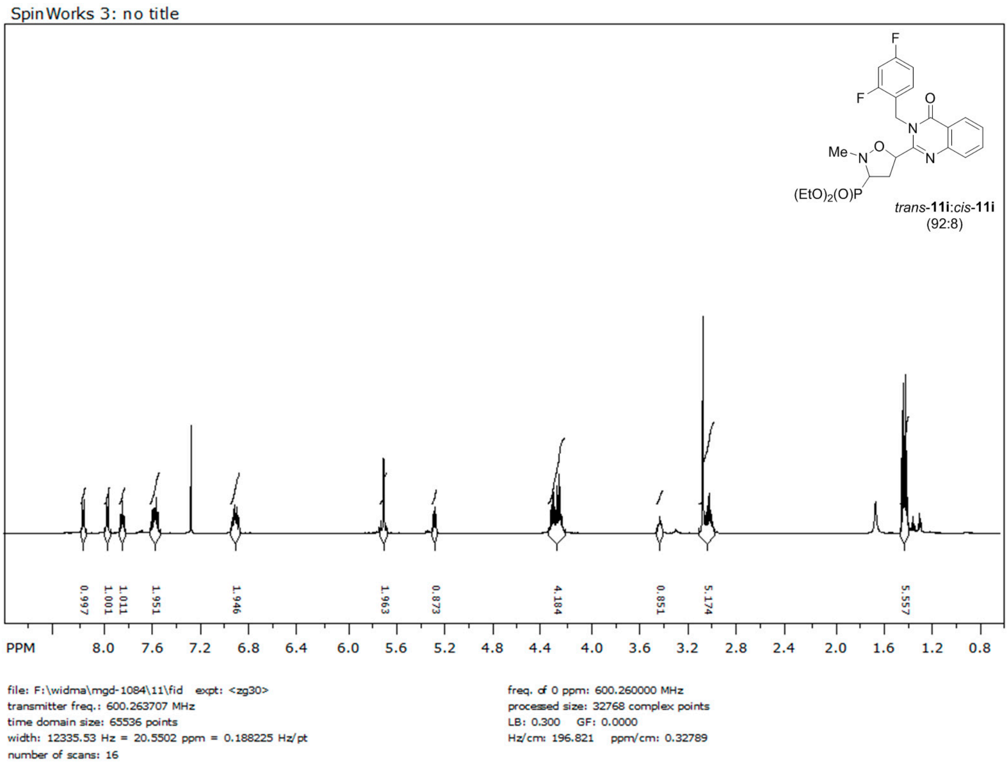

Figure S28. The ^{1}H-NMR spectrum of a 92:8 mixture of isoxazolidines *trans*-**11i** and *cis*-**11i**.

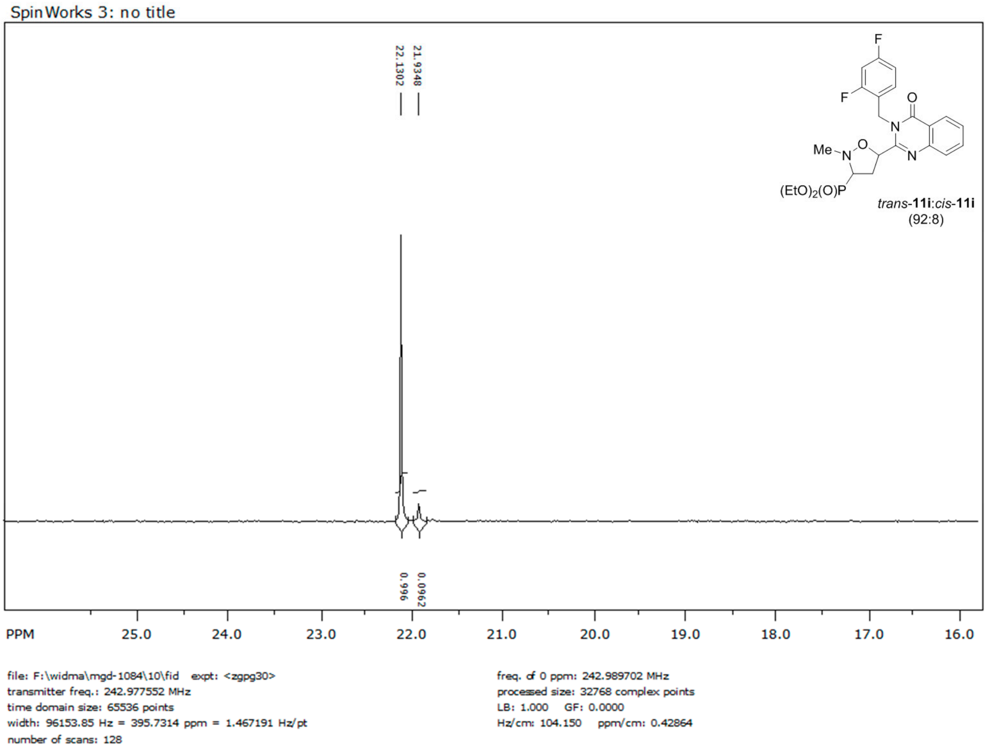

Figure S29. The ^{31}P-NMR spectrum of a 92:8 mixture of isoxazolidines *trans*-**11i** and *cis*-**11i**.

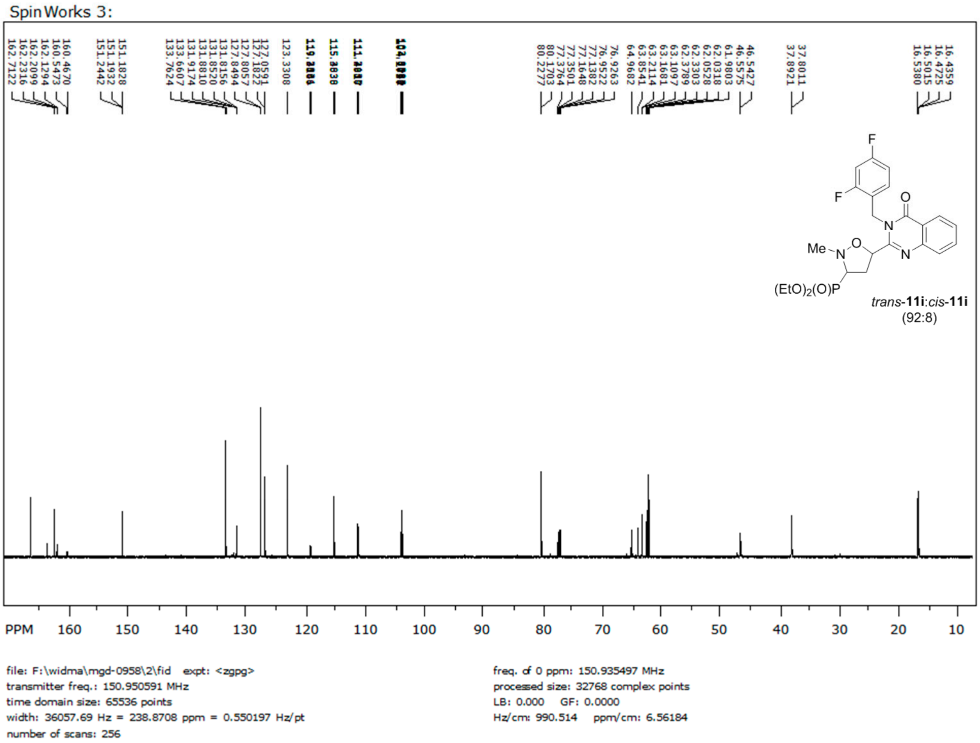

Figure S30. The ^{13}C-NMR spectrum of a 92:8 mixture of isoxazolidines *trans*-**11i** and *cis*-**11i**.

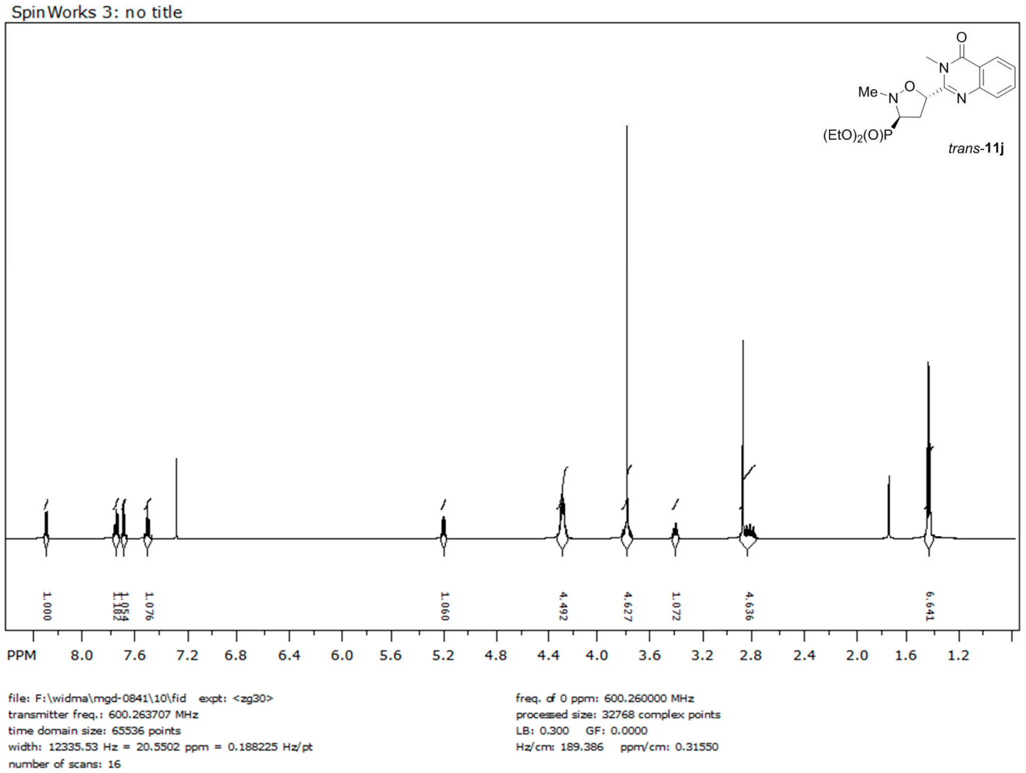

Figure S31. The ^{1}H-NMR spectrum of isoxazolidine *trans*-**11j**.

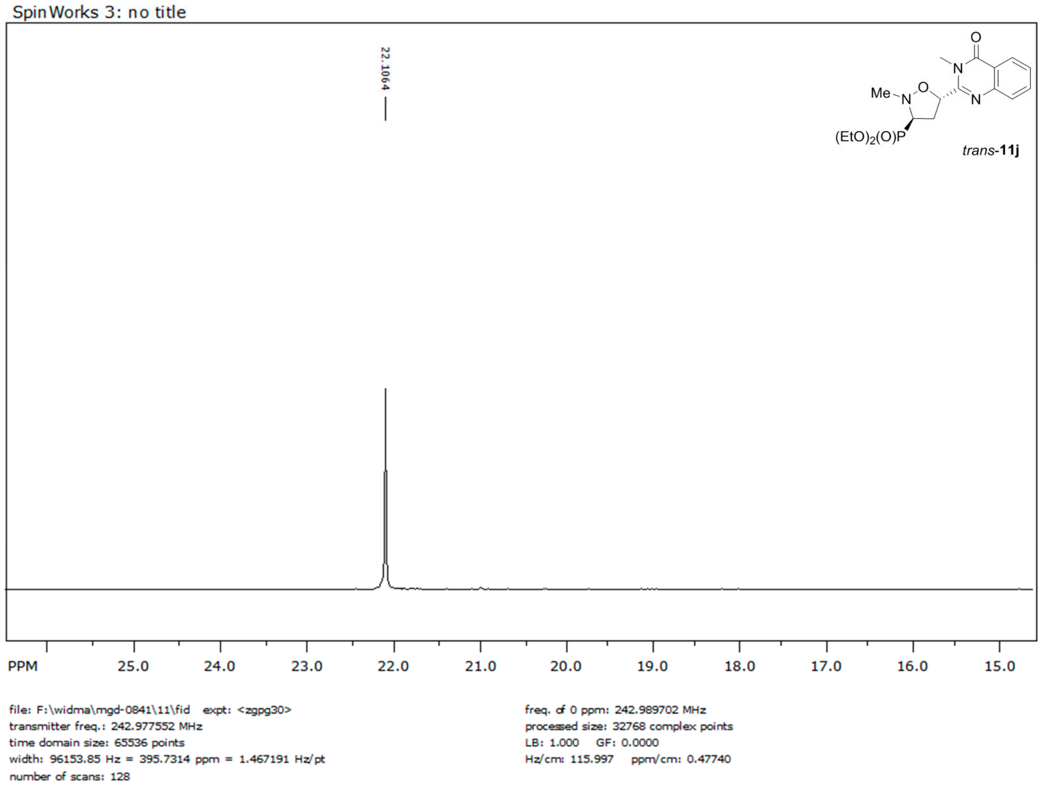

Figure S32. The ^{31}P-NMR spectrum of isoxazolidine *trans*-**11j**.

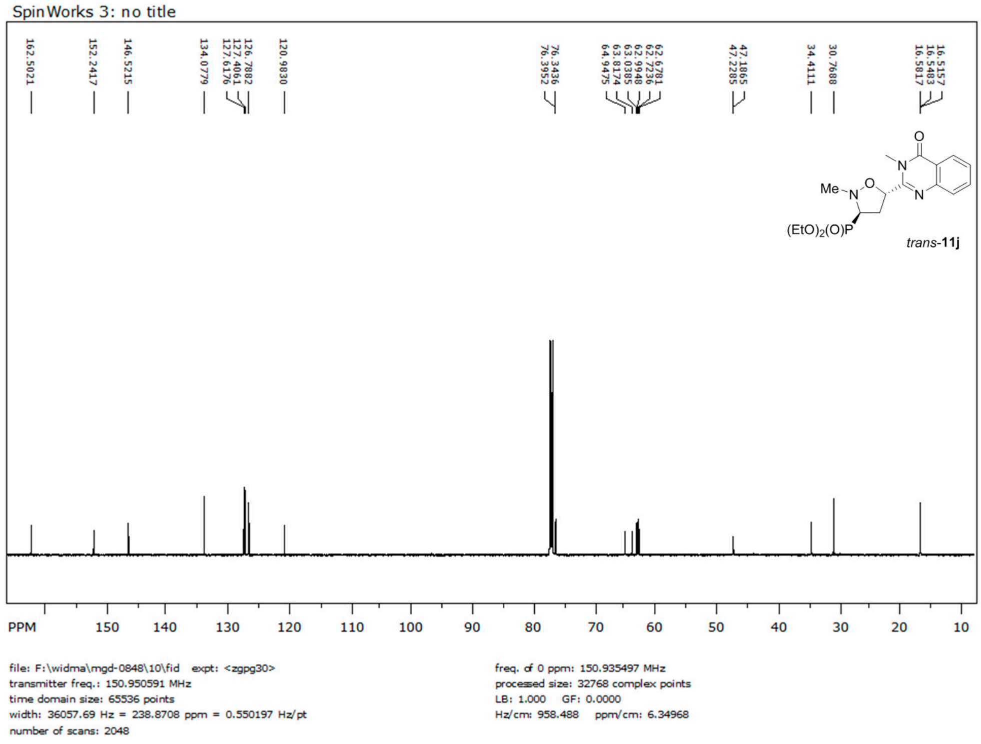

Figure S33. The ^{13}C-NMR spectrum of isoxazolidine *trans*-**11j**.

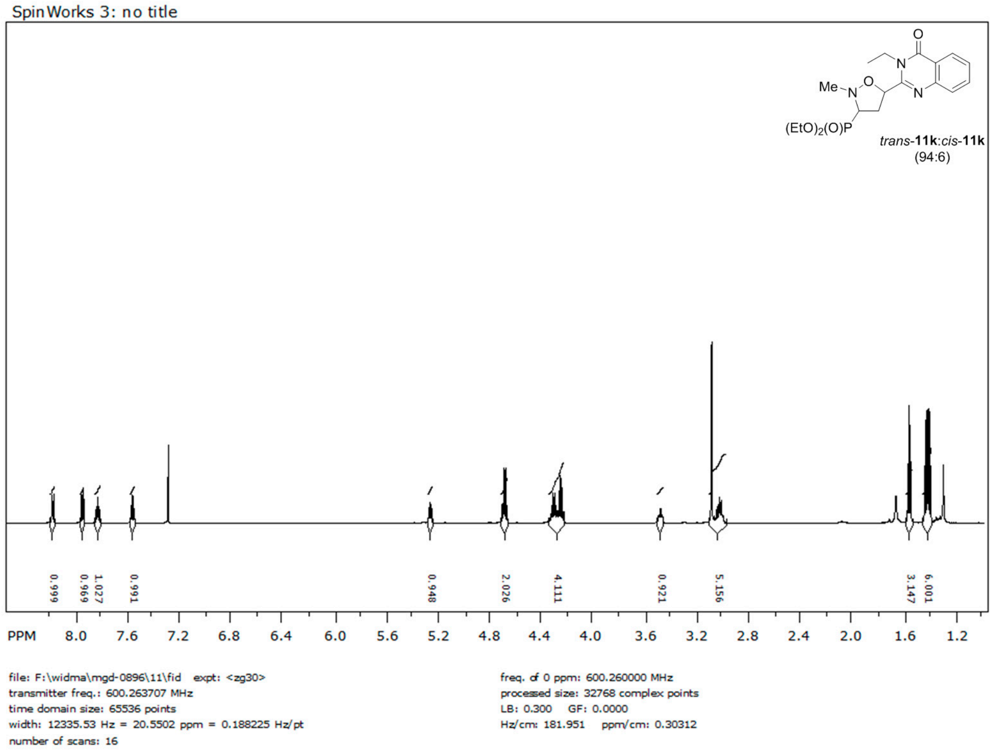

Figure S34. The ^{1}H-NMR spectrum of a 94:6 mixture of isoxazolidines *trans*-**11k** and *cis*-**11k**.

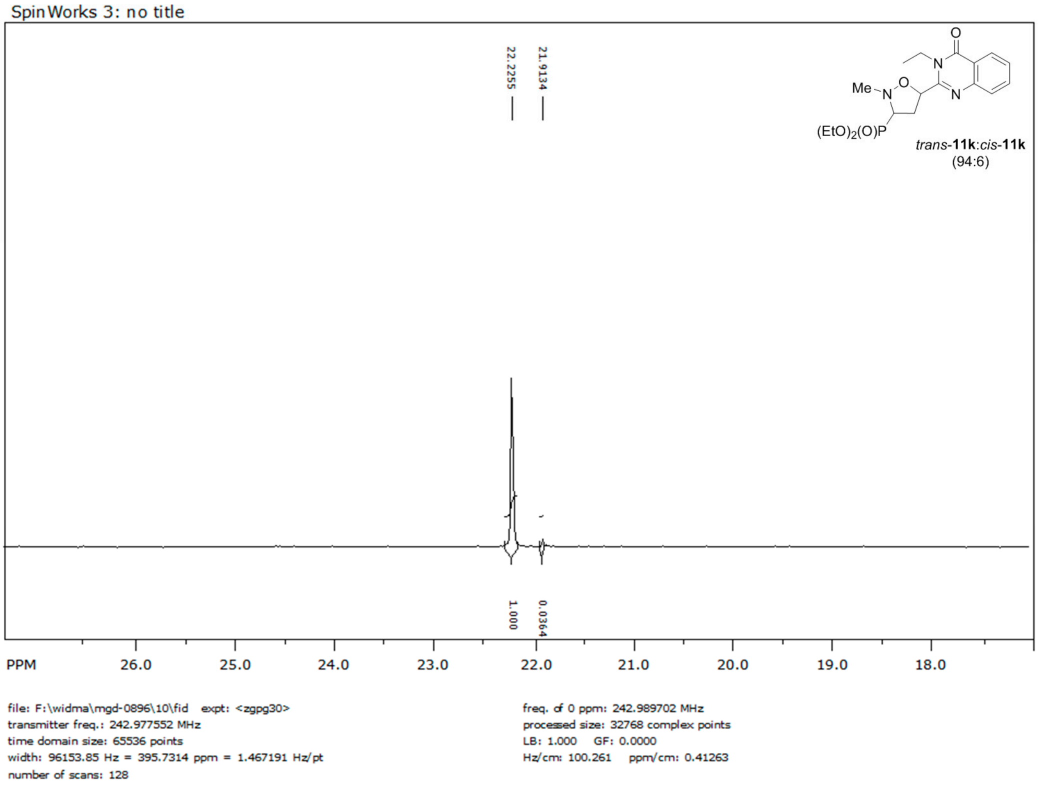

Figure S35. The ^{31}P-NMR spectrum of a 94:6 mixture of isoxazolidines *trans*-**11k** and *cis*-**11k**.

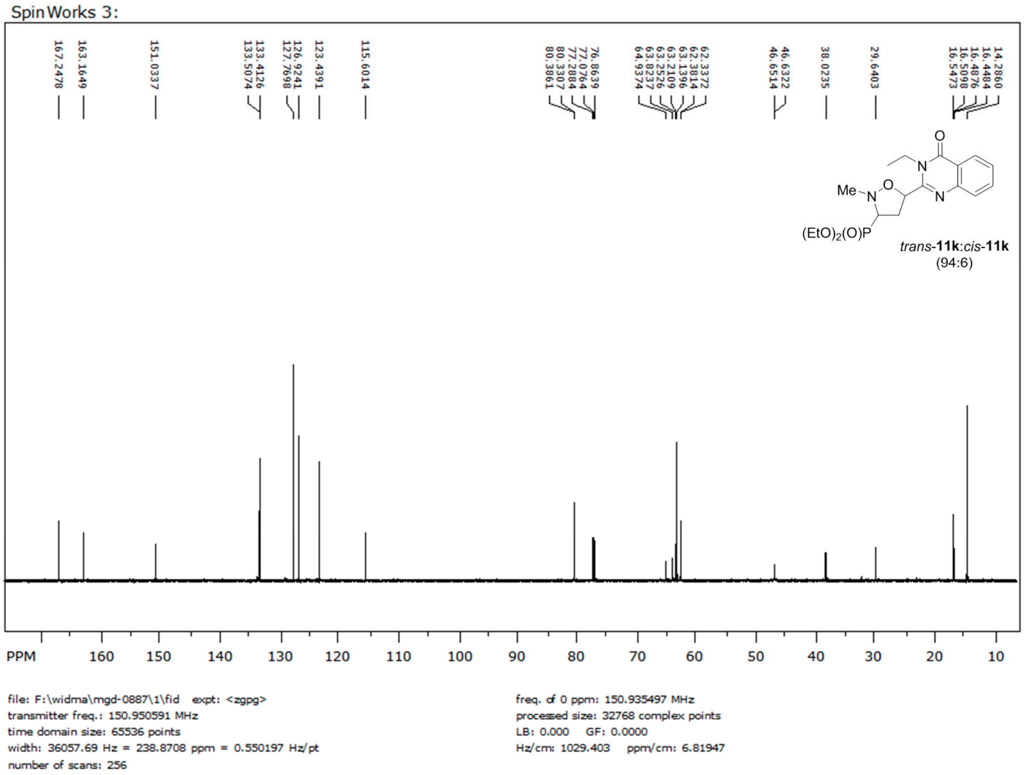

Figure S36. The ^{13}C-NMR spectrum of a 94:6 mixture of isoxazolidines *trans*-**11k** and *cis*-**11k**.

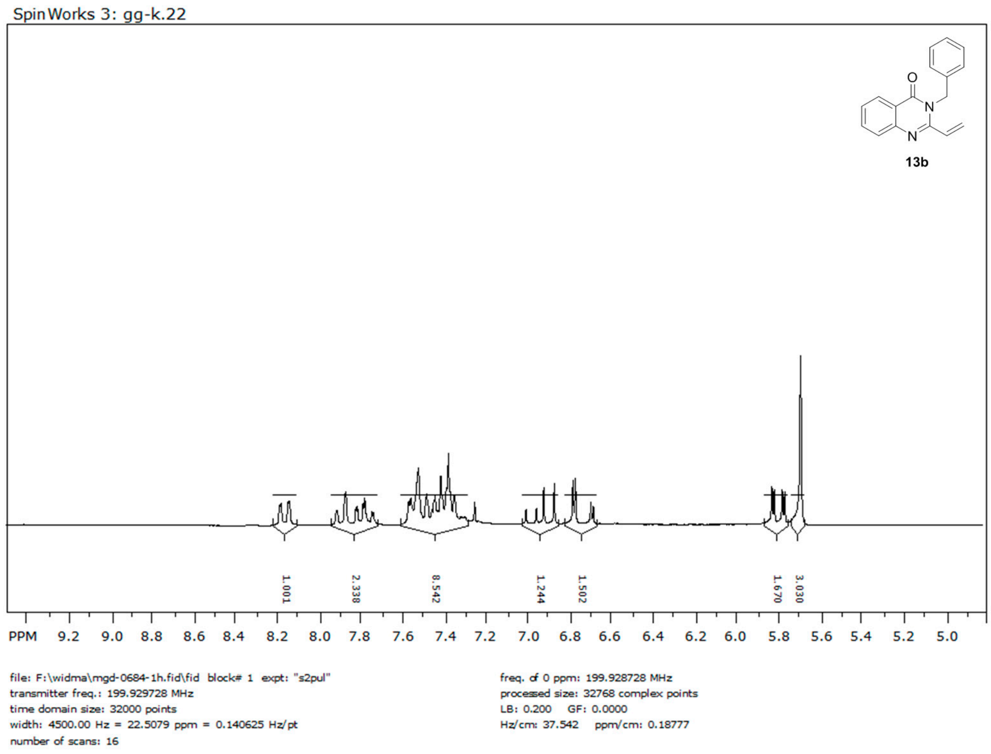

Figure S37. The ^{1}H-NMR spectrum of compound **13b**.

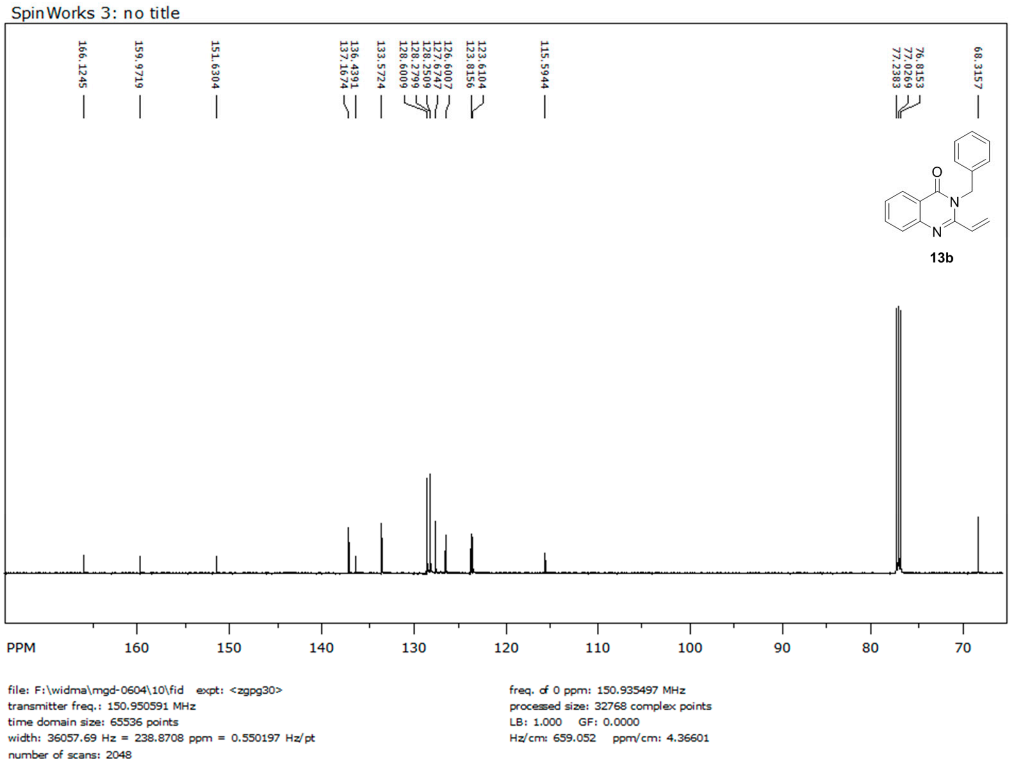

Figure S38. The ^{13}C-NMR spectrum of compound **13b**.

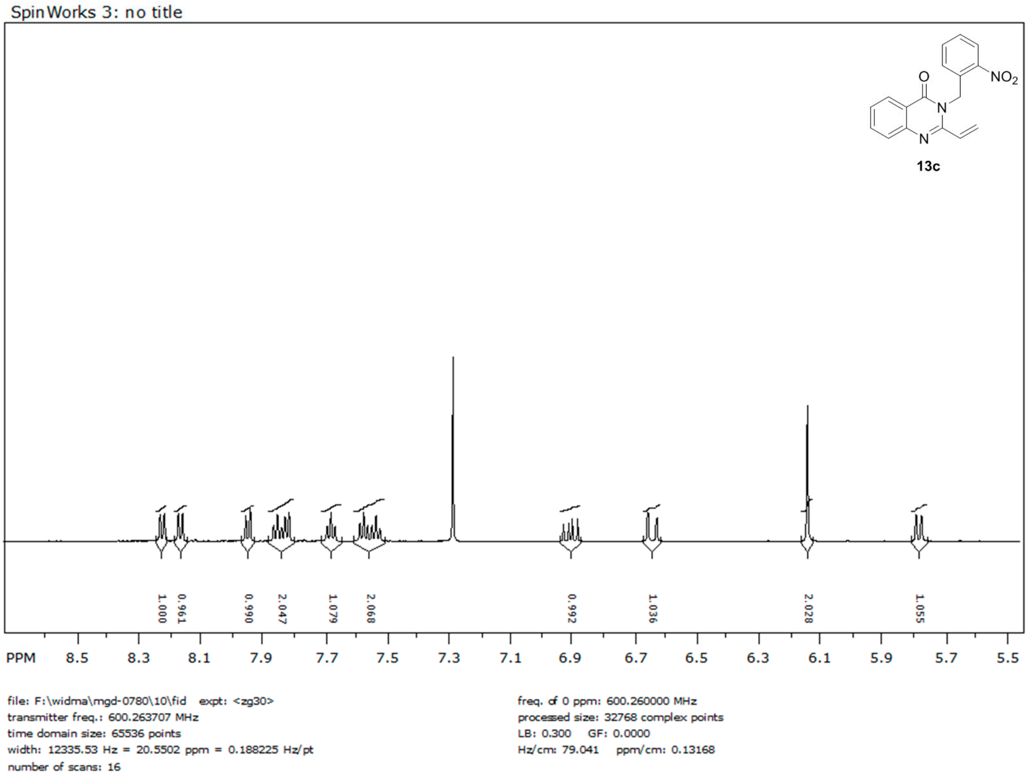

Figure S39. The ^{1}H-NMR spectrum of compound **13c**.

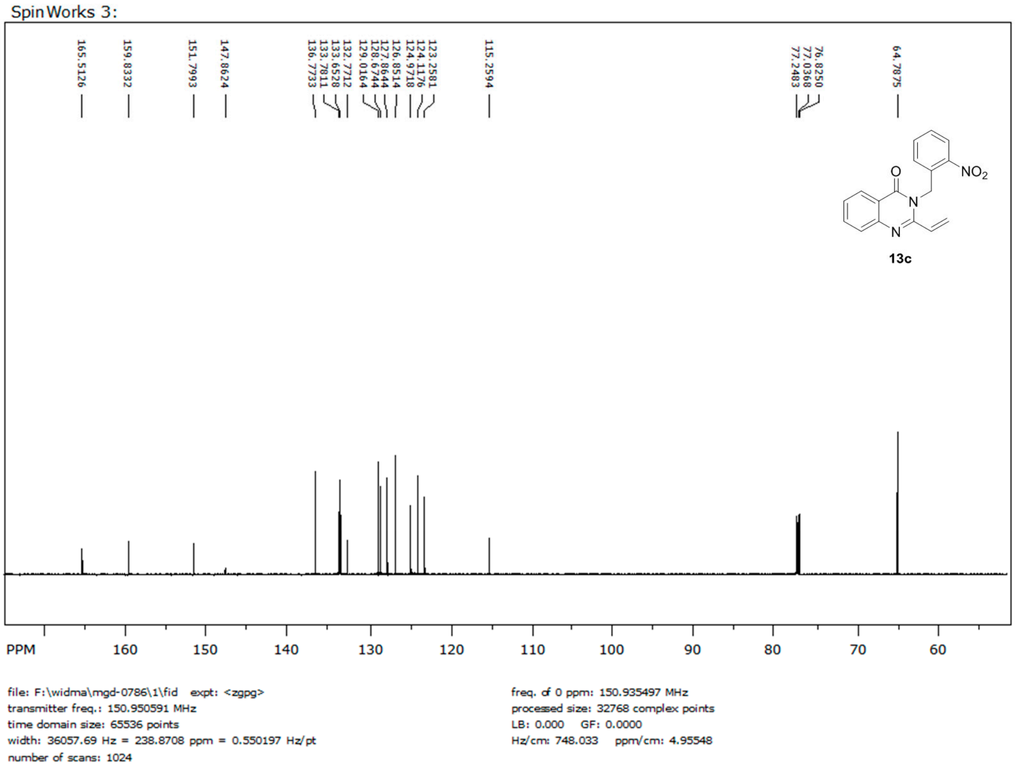

Figure S40. The ^{13}C-NMR spectrum of compound **13c**.

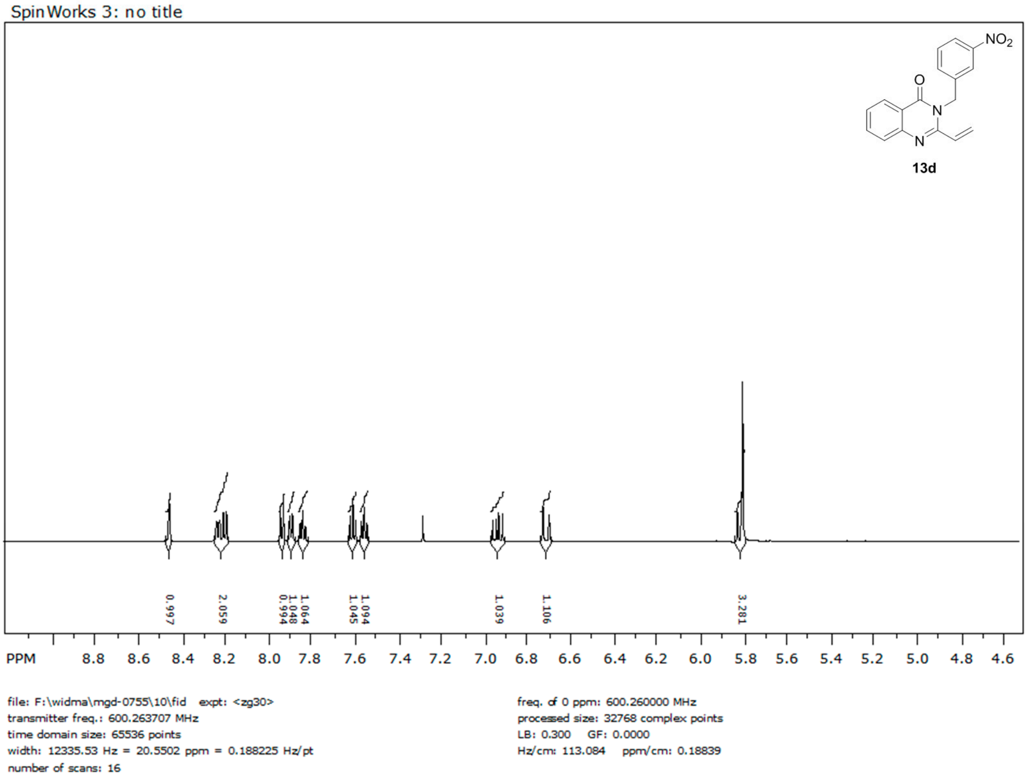

Figure S41. The ^{1}H-NMR spectrum of compound **13d**.

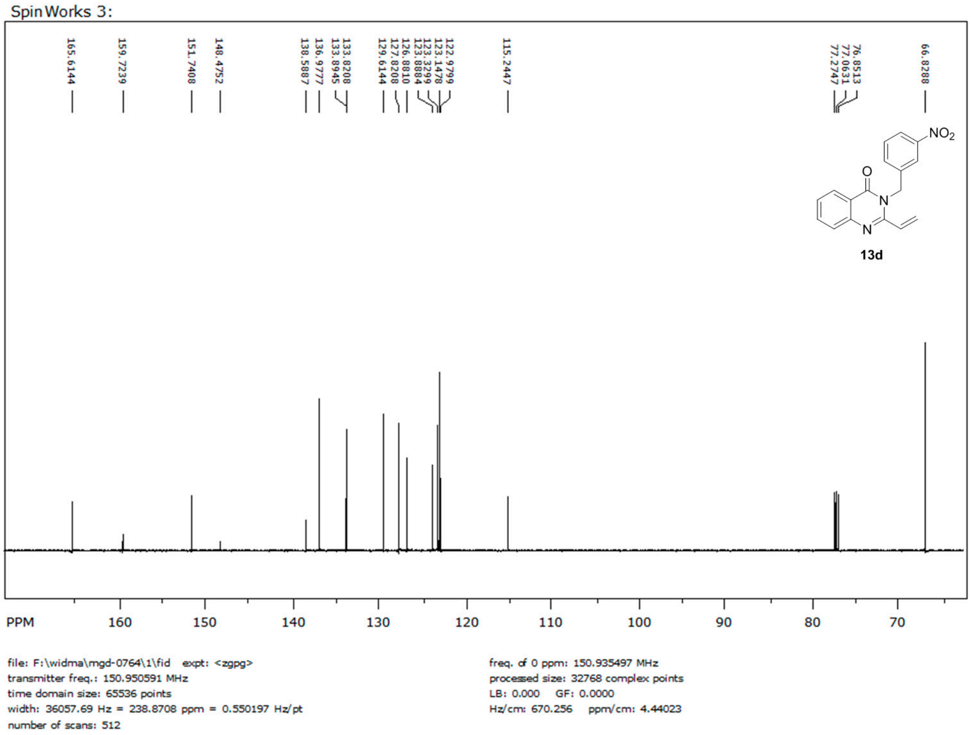

Figure S42. The ^{13}C-NMR spectrum of compound **13d**.

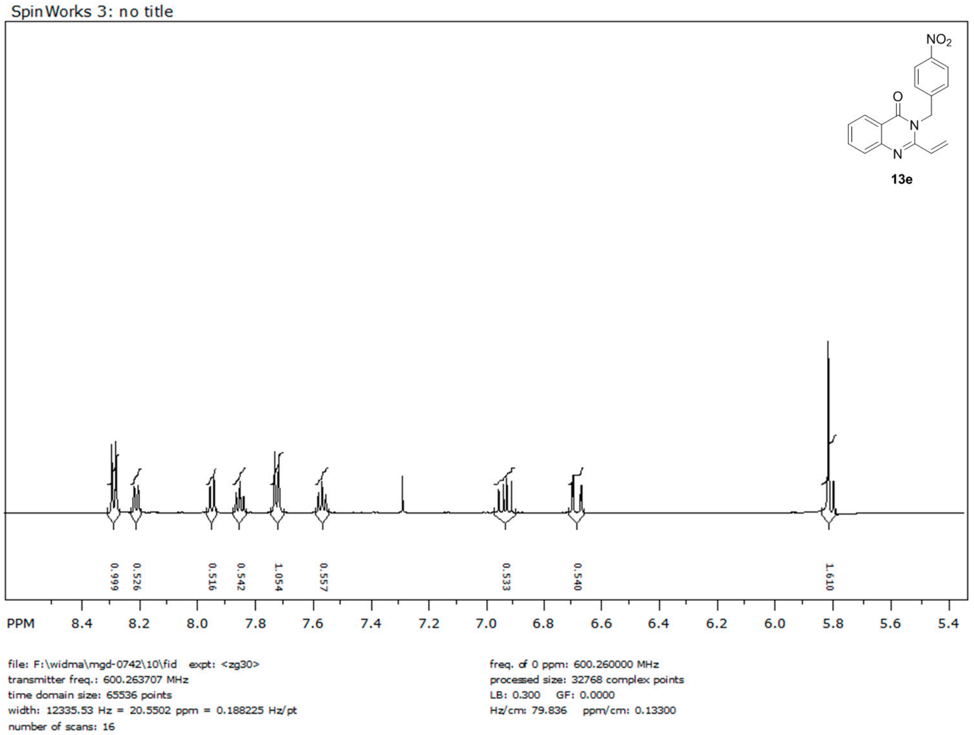

Figure S43. The ^{1}H-NMR spectrum of compound **13e**.

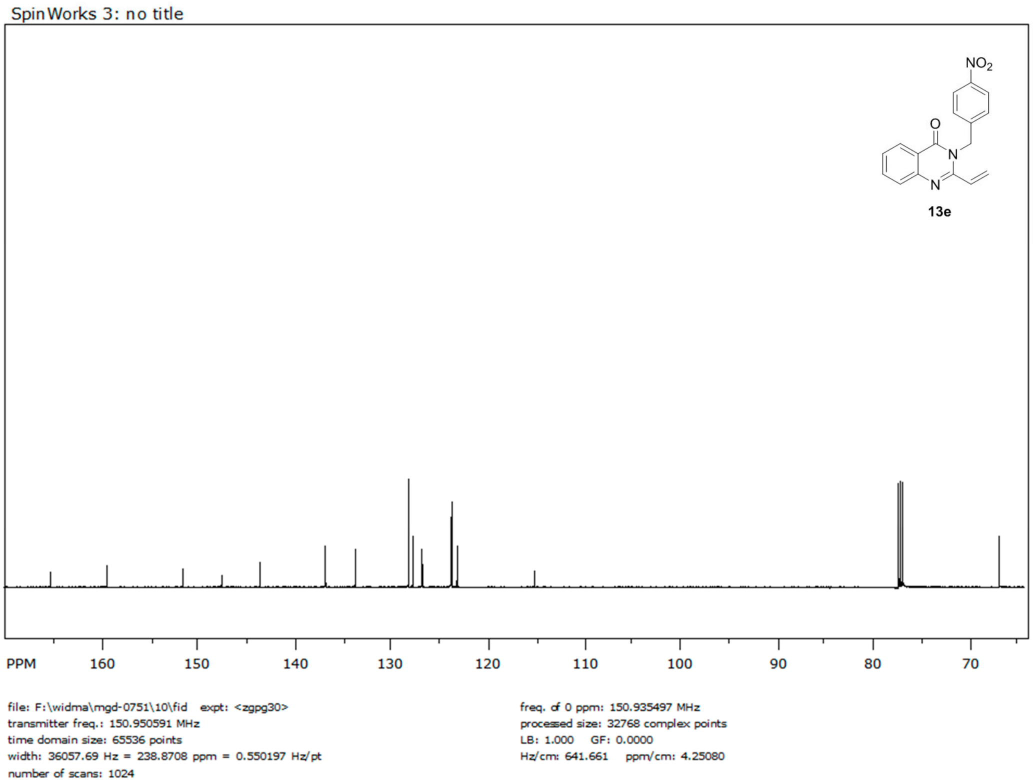

Figure S44. The ^{13}C-NMR spectrum of compound **13e**.

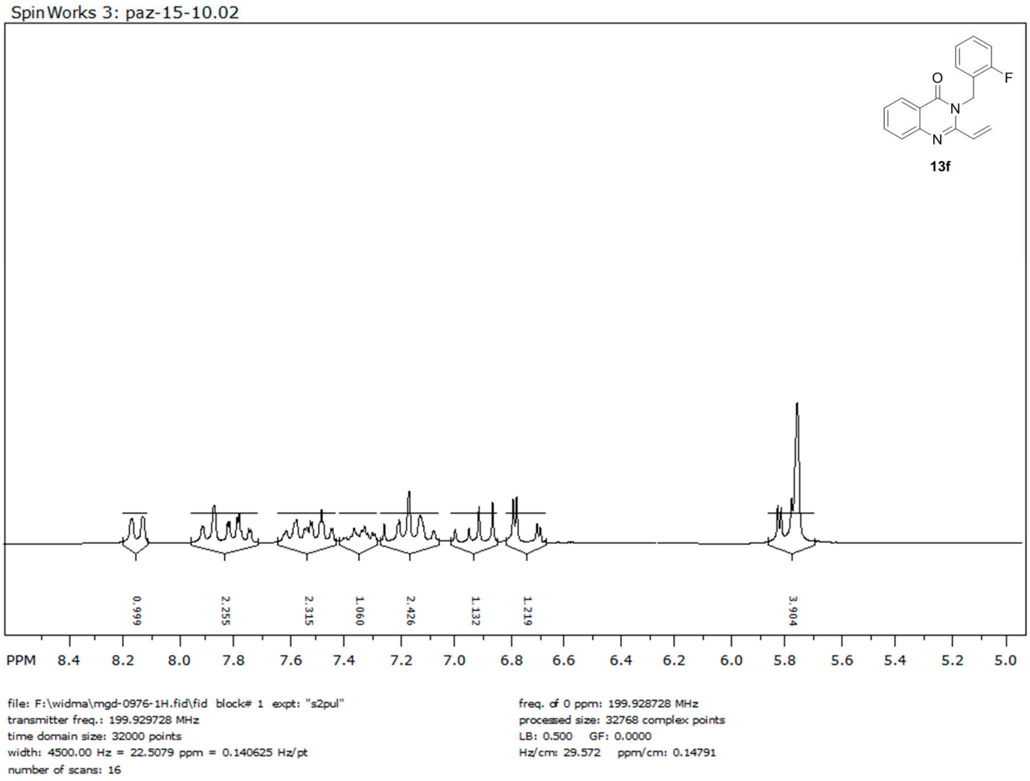

Figure S45. The ^{1}H-NMR spectrum of compound **13f**.

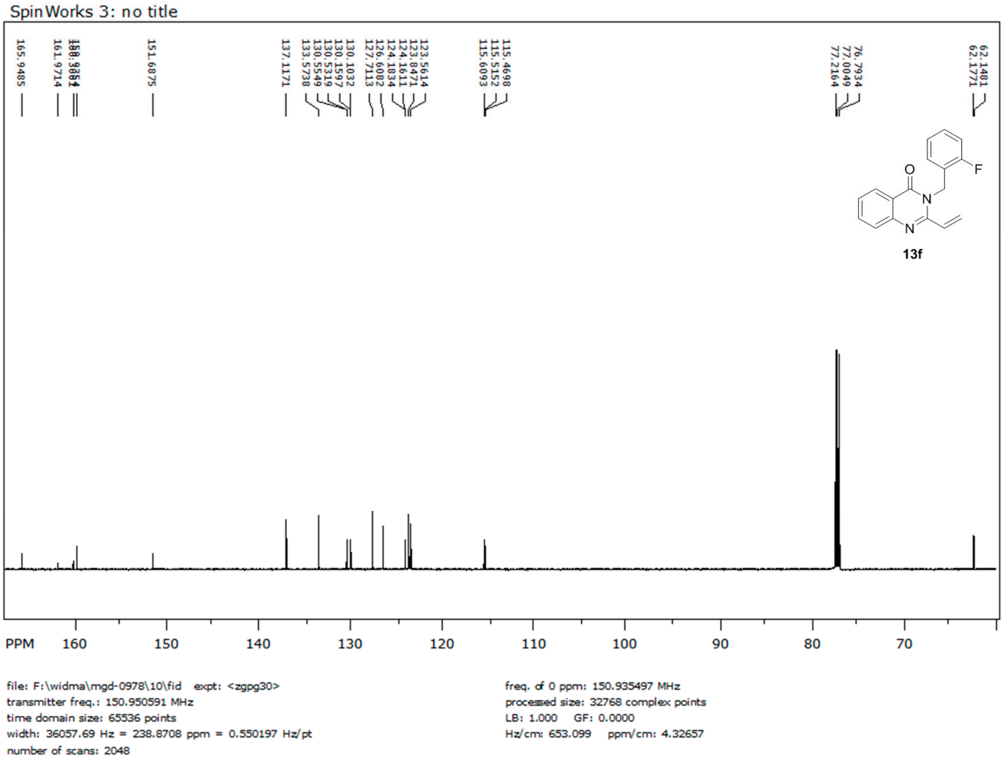

Figure S46. The ^{13}C-NMR spectrum of compound **13f**.

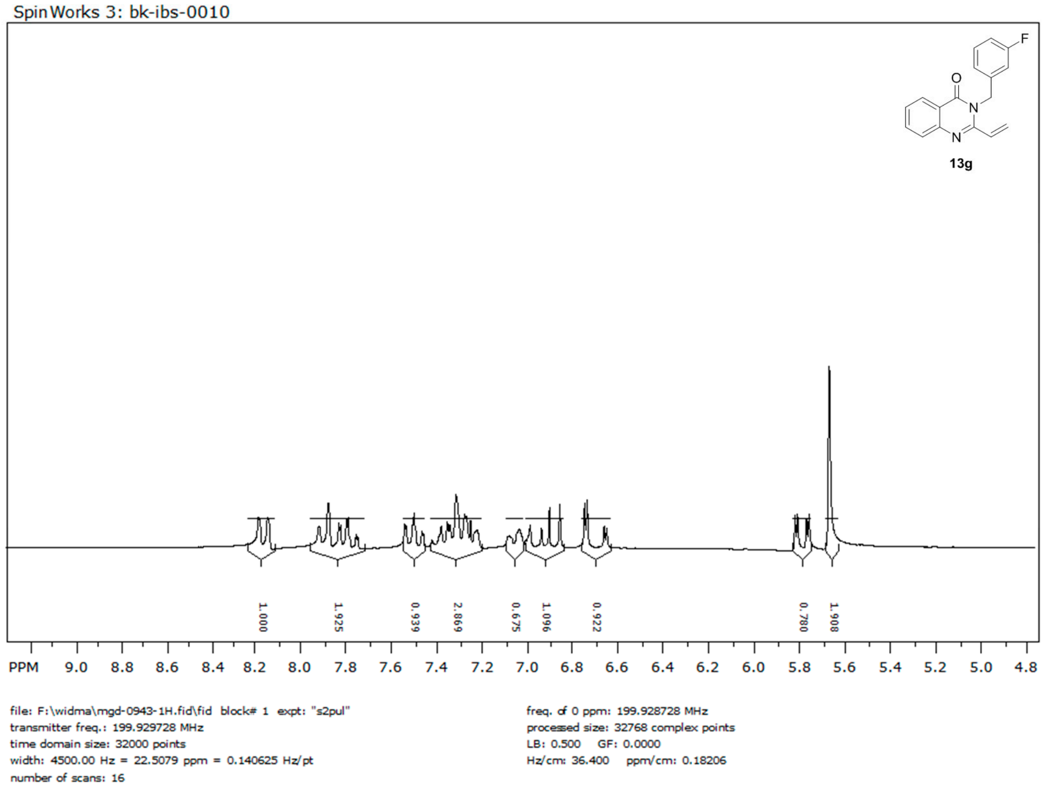

Figure S47. The ^{1}H-NMR spectrum of compound **13g**.

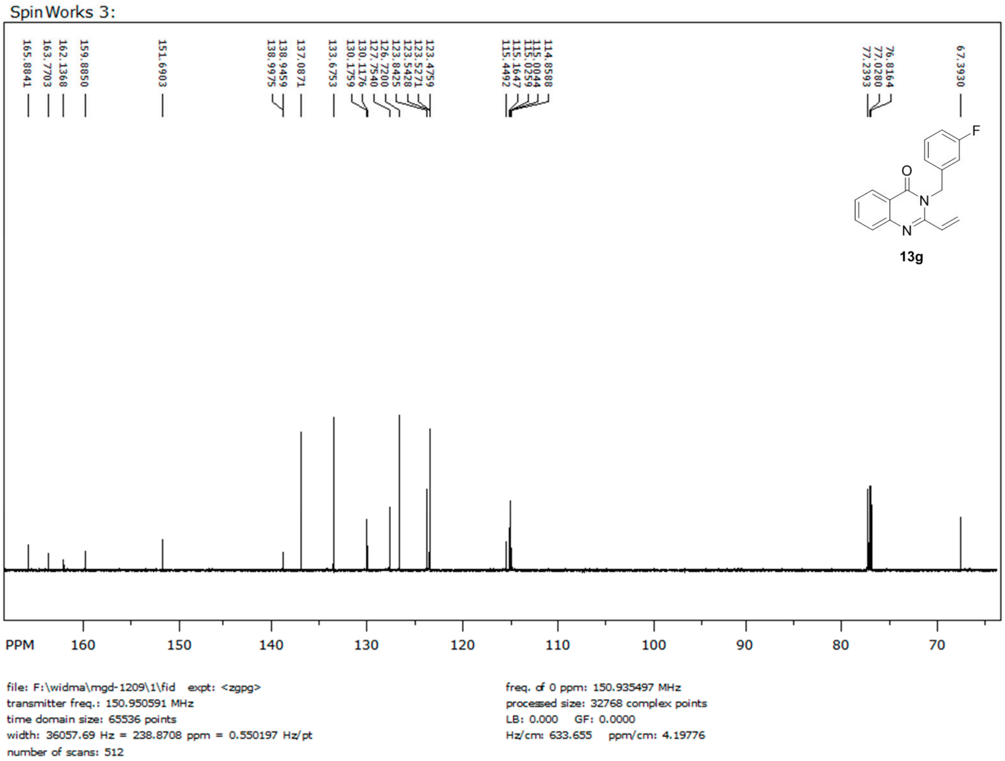

Figure S48. The ^{13}C-NMR spectrum of compound **13g**.

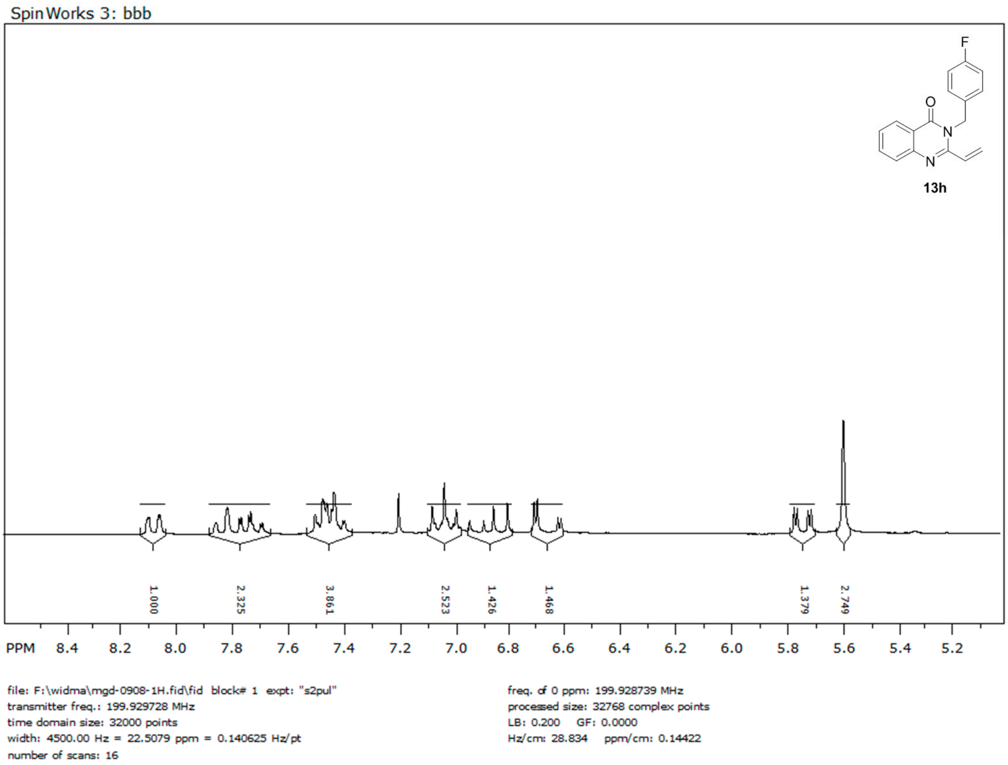

Figure S49. The ^{1}H-NMR spectrum of compound **13h**.

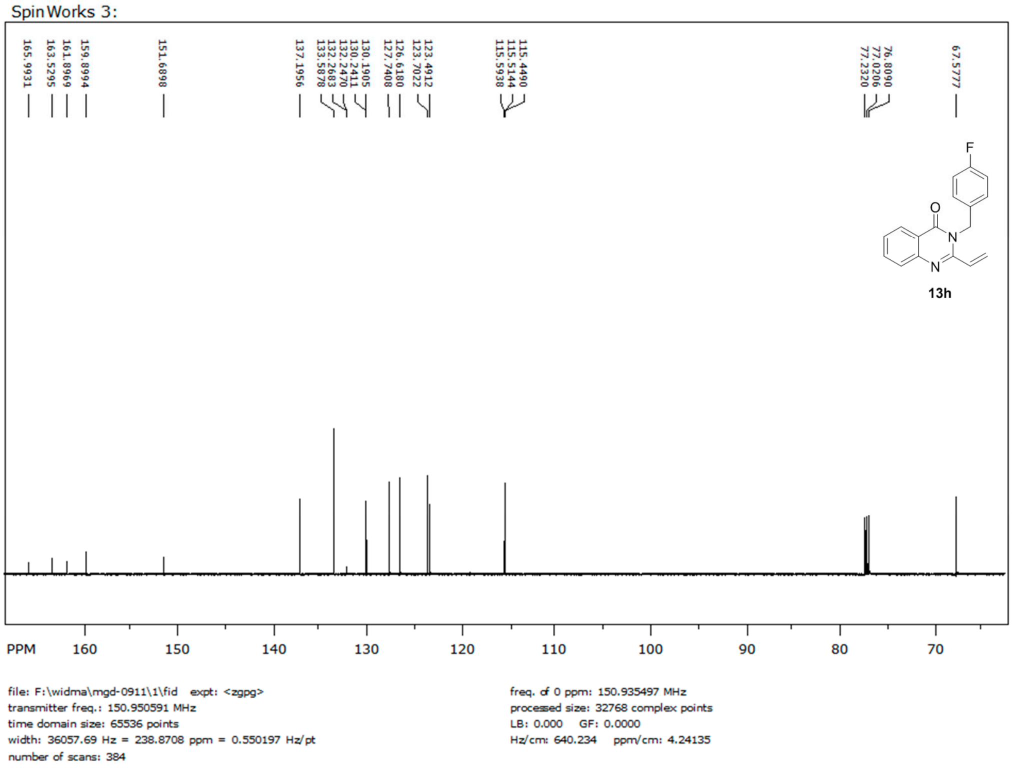

Figure S50. The ^{13}C-NMR spectrum of compound **13h**.

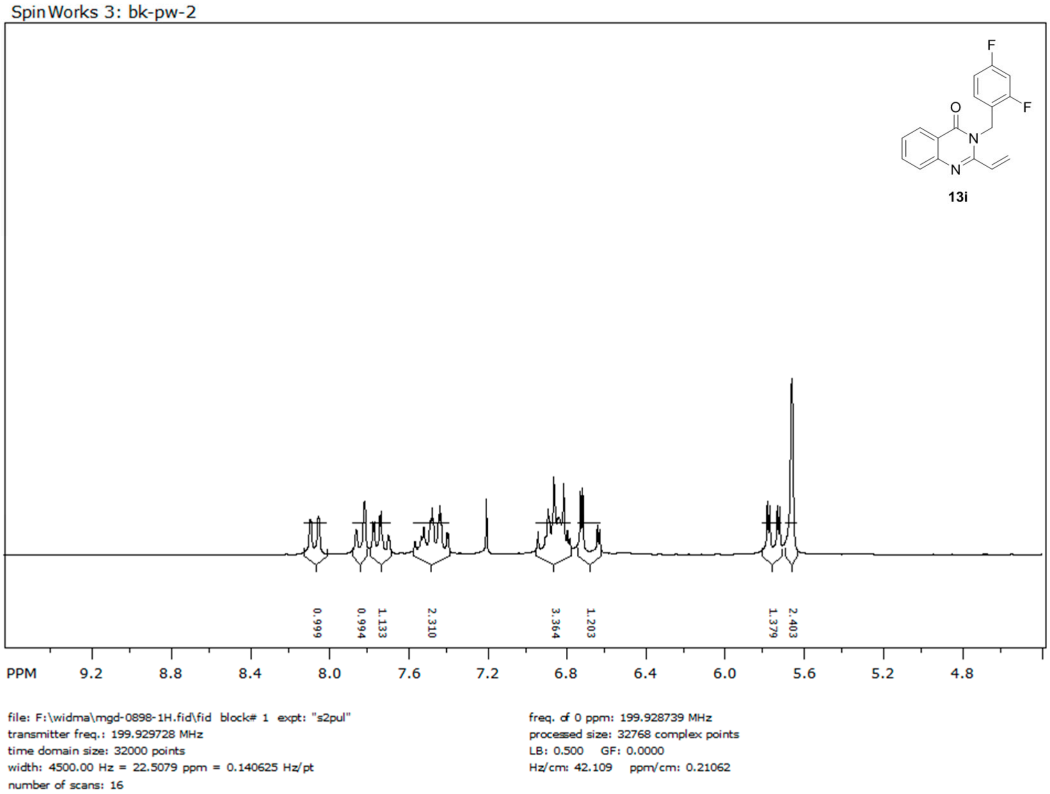

Figure S51. The ^{1}H-NMR spectrum of compound **13i**.

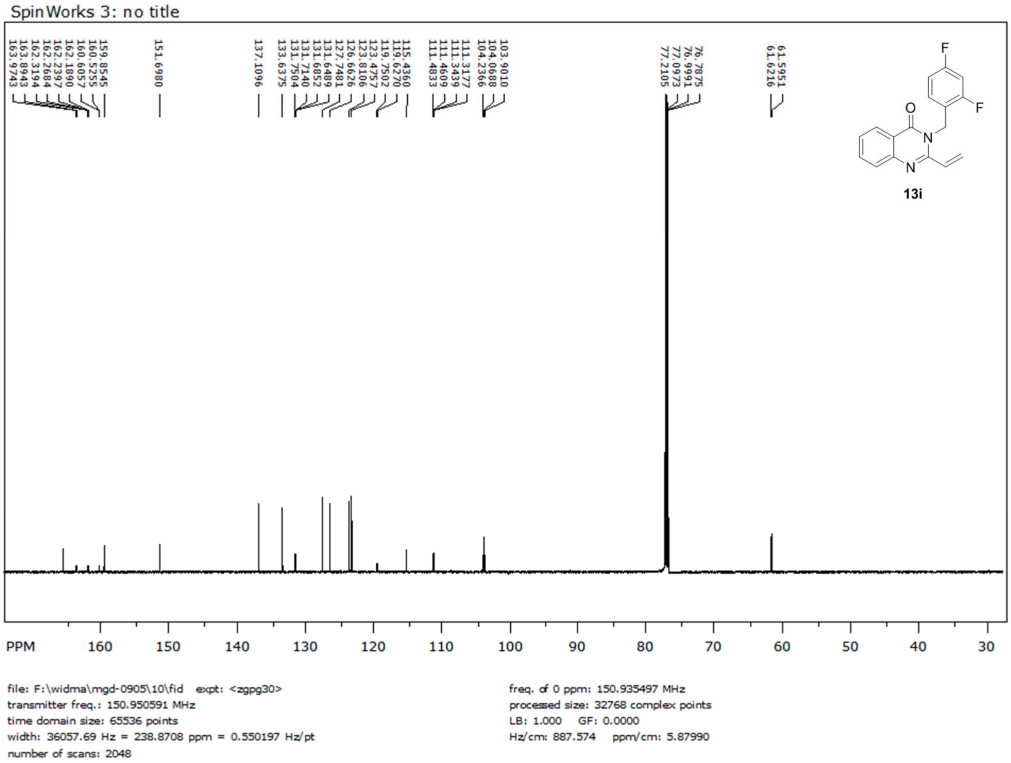

Figure S52. The ^{13}C-NMR spectrum of compound **13i**.

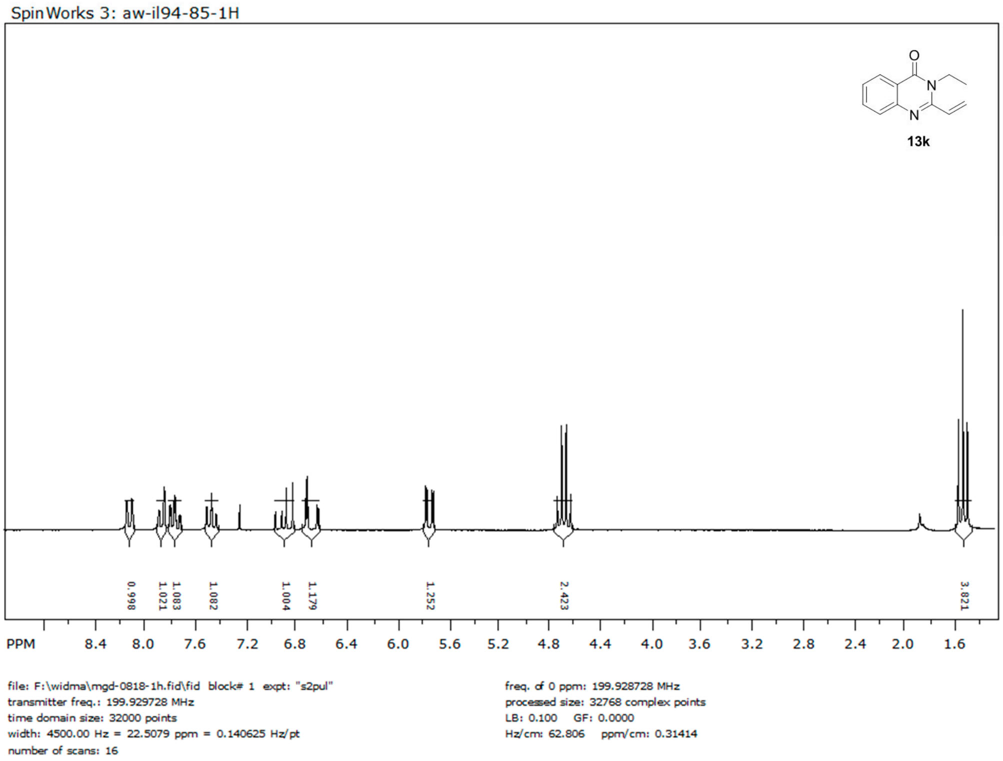

Figure S53. The ^{1}H-NMR spectrum of compound **13k**.

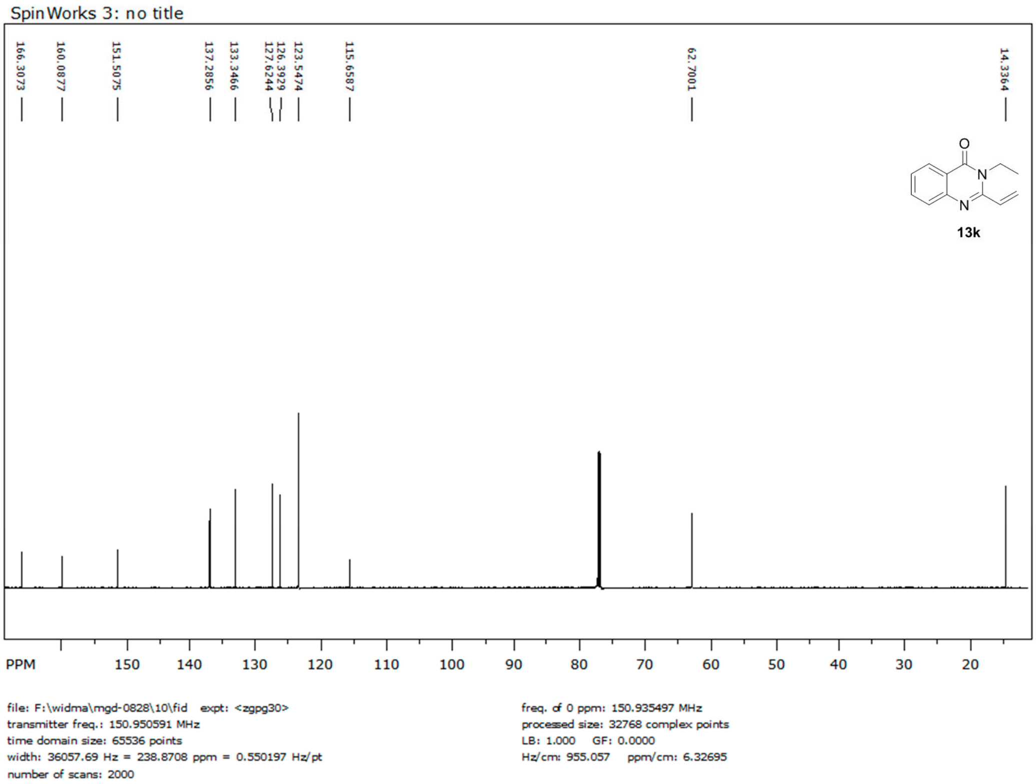

Figure S54. The ^{13}C-NMR spectrum of compound **13k**.
